# CCDC138 overexpression predicts poor prognosis and highlights ciliopathy-linked mechanisms in uterine corpus endometrial carcinoma

**DOI:** 10.3389/fmolb.2025.1622496

**Published:** 2025-08-08

**Authors:** Aiping Wang, Fang Yang, Chunhua Zhang, Shi Li, Han Fu

**Affiliations:** ^1^ Shaoxing Maternity and Child Health Care Hospital, Maternity and Child Health Care Affiliated Hospital, Shaoxing University, Shaoxing, China; ^2^ Department of Urology, The First Affiliated Hospital of Wenzhou Medical University, Wenzhou, China; ^3^ The First Affiliated Hospital of Wenzhou Medical University, Wenzhou Medical University, Wenzhou, China

**Keywords:** uterine corpus endometrial carcinoma, CCDC138, ciliopathy, biomarker, prognosis

## Abstract

**Introduction:**

Uterine corpus endometrial carcinoma (UCEC) is a prevalent malignancy of the female reproductive system with increasing incidence, necessitating the identification of molecular mechanisms and biomarkers. While coiled-coil domain-containing protein 138 (CCDC138) is implicated in ciliopathies and cancer, its role in UCEC remains underexplored.

**Methods:**

We integrated transcriptomic and proteomic data from the Cancer Genome Atlas (TCGA), Clinical Proteomic Tumor Analysis Consortium (CPTAC), and Genotype-Tissue Expression (GTEx). Bioinformatics approaches, including weighted gene co-expression network analysis (WGCNA), singlesample gene set enrichment analysis (ssGSEA), machine learning, and survival analysis, were employed to assess CCDC138 expression and its functional relevance in UCEC. *In vitro* experiments involved CCDC138 knockdown, followed by CCK8 and EdU assays and qPCR for mTOR, S6K1, and p21 expression.

**Results:**

CCDC138 was significantly overexpressed at mRNA and protein levels in UCEC and correlated with poor overall survival. ssGSEA revealed associations with oncogenic pathways, including mTOR, p53/Rb, and MYC/MYCN. High CCDC138 expression was linked to reduced stromal and immune scores, indicating altered immune cell infiltration and tumor microenvironment. Drug sensitivity analysis showed increased responsiveness to chemotherapeutic agents like 5-fluorouracil and alpelisib in high-CCDC138 tumors. Protein-protein interaction analysis identified interactions with DCTN2 and CEP72. *In vitro*, CCDC138 knockdown reduced cell proliferation and downregulated mTOR, S6K1, and p21 mRNA expression.

**Discussion:**

These findings underscore CCDC138’s role in UCEC progression, immune modulation, and therapeutic responsiveness, highlighting its potential as a prognostic biomarker and therapeutic target. Its shared relevance in UCEC and ciliopathies suggests broader implications for targeted therapies.

## 1 Introduction

Uterine corpus endometrial carcinoma (UCEC) is one of the most prevalent gynecologic malignancies, with a steadily increasing incidence worldwide, particularly in developed countries (Lortet-Tieulent et al., 2018; Huang et al., 2022). While early-stage UCEC is often curable through surgery and adjuvant therapy, treatment options for advanced or recurrent cases remain limited, leading to poor overall survival. Understanding the molecular mechanisms of UCEC and identifying robust biomarkers and therapeutic targets are essential for improving outcomes and advancing precision medicine.

Advancements in high-throughput omics technologies and bioinformatics have provided new insights into cancer biology. Integrative tools, such as weighted gene co-expression network analysis (WGCNA), single-sample gene set enrichment analysis (ssGSEA), and machine learning algorithms, have facilitated the discovery of key regulatory genes and oncogenic pathways. In UCEC, increasing evidence links various forms of programmed cell death—including disulfidptosis, cuproptosis, and ferroptosis—with tumorigenesis, immune modulation, and drug sensitivity (Shi et al., 2018).

Cilia-related genes play a vital role in cancer development and progression. Coiled-coil domain-containing protein 138 (CCDC138), known for its role in ciliogenesis, has recently gained attention due to its involvement in cancer (Drew et al., 2017; Anurag et al., 2024). Ciliopathies, inherited disorders caused by structural or functional defects in cilia—organelles critical for cell signaling, differentiation, and homeostasis—have been associated with disrupted cellular sensing, signal transduction, and tissue homeostasis. Although aberrant ciliary signaling has been implicated in oncogenesis, the role of CCDC138 in UCEC and its link to ciliopathy-related mechanisms remain poorly understood (Pontén et al., 2009).

To address this, we conducted a comprehensive analysis integrating transcriptomic and proteomic data from The Cancer Genome Atlas (TCGA) and Clinical Proteomic Tumor Analysis Consortium (CPTAC) databases. Using WGCNA and machine learning, we identified UCEC-associated gene modules and prioritized candidate genes. We focused on CCDC138 to examine its expression patterns, prognostic value, association with oncogenic and cilia-related pathways, immune cell infiltration, tumor microenvironment (TME) regulation, and drug sensitivity. To validate these findings, we conducted *in vitro* experiments in Ishikawa cells, assessing the effects of CCDC138 knockdown on cell proliferation and oncogenic pathway gene expression (mTOR, S6K1, p21). These findings position CCDC138 as a potential shared target in UCEC and ciliopathies, offering novel insights into its contribution to tumor progression and opportunities for precision oncology based on ciliopathy-related mechanisms.

## 2 Materials and methods

### 2.1 Data acquisition and preprocessing

RNA sequencing data for uterine UCEC were obtained from the TCGA-UCEC cohort within TCGA database (https://portal.gdc.cancer.gov/). Raw count data were converted into transcripts per million (TPM) to construct the expression matrix. Following quality control and filtering, 584 samples (including tumor and adjacent normal tissues) and 60,616 genes were retained for downstream analyses. To normalize expression levels and reduce data skewness, the TPM matrix was log-transformed using the formula log2 (TPM +1) (Zhang et al., 2020). Genes with low-expression (mean TPM <1 across all samples) were excluded, yielding a final dataset suitable for subsequent analyses. To validate transcriptomic findings at the protein level, clinical and proteomic data for UCEC were retrieved from the CPTAC via the UALCAN portal (http://ualcan.path.uab.edu/) (Chandrashekar et al., 2017; Chandrashekar et al., 2022). In addition, transcriptomic data from the Genotype-Tissue Expression (GTEx) database were integrated to compare CCDC138 expression across 33 cancer types (Coorens et al., 2025).

### 2.2 ssGSEA

ssGSEA was performed using the GSVA package (version 1.38.2) in R (version 4.2.1) to quantify the enrichment levels of specific biological pathways (Hänzelmann et al., 2013). Gene sets related to disulfidptosis, cuproptosis, and ferroptosis were curated from the Molecular Signature Database (MSigDB, version 7.5.1). Enrichment scores were computed for each sample, and hierarchical clustering was conducted to identify differential enrichment patterns between tumor and normal tissues. Heatmaps were generated using the pheatmap package (version 1.0.12) for visualization. Statistical significance was assessed using the Wilcoxon rank-sum test, with p < 0.05 considered significant.

### 2.3 WGCNA

To investigate gene co-expression patterns in TCGA-UCEC data, WGCNA was performed using the WGCNA package in R (Langfelder and Horvath, 2008). The log-transformed TPM expression matrix was used as input after filtering for genes with a mean TPM >1. Sample outliers were removed based on a WGCNA of 137,000. An appropriate soft-thresholding power (β) was determined using the pickSoftThreshold function to approximate a scale-free network topology. Gene modules were identified using the dynamic tree cut algorithm (cutreeDynamic), with the minimum module size set to 100. Module similarity was assessed through eigengene clustering, and correlations between gene modules and clinical traits were evaluated via correlation analysis. The blue module, which showed the strongest correlation with tumor traits, was selected for further analysis. Heatmaps and scatter plots were generated for data visualization.

### 2.4 Differentially expressed gene (DEG) analysis

DEGs between tumor and normal samples were identified based on the gene expression data derived from WGCNA. Gene expression values were normalized using the trimmed mean of M-values method and converted to counts per million. Genes with low expression were filtered out before analysis (Robinson and Oshlack, 2010). For each gene, fold change and false discovery rate (FDR) were computed, and those meeting the criteria of |log_2_ fold change (log_2_FC)| > 1 and FDR <0.05 were considered significantly differentially expressed (Zhao et al., 2021). Volcano plots were generated to visualize differential expression patterns, with significantly upregulated and downregulated genes annotated. Boxplots were used to compare the expression levels of selected DEGs between tumor and normal groups. The resulting DEG expression matrix was retained for subsequent analyses. DEG results were saved in text format, and the resulting DEG expression matrix was retained for downstream analyses.

### 2.5 Feature gene selection using machine learning

To identify key feature genes among the 46 DEGs, five machine learning algorithms were employed: gradient boosting machine (GBM), random forest (RF), support vector machine-recursive feature elimination (SVM-RFE), least absolute shrinkage and selection operator (Lasso), and extreme gradient boosting (XGBoost) (Sanz et al., 2018; Tibshirani, 1996) (Becker et al., 2023; Chen and Guestrin, 2016). These analyses were conducted using the caret (version 6.0–90), randomForest (version 4.6–14), e1071 (version 1.7–9), glmnet (version 4.1–2), and XGBoost (version 1.5.0.2) packages in R. Each algorithm ranked genes based on their contribution to classification accuracy. Venn diagrams were generated using the VennDiagram package (version 1.7.1) to identify overlapping feature genes across all methods. The expression profiles of the consensus genes were retained for downstream analyses.

### 2.6 Survival analysis

Overall survival (OS) analysis was performed using the survival (version 3.2–13) and survminer (version 0.4.9) packages in R. The prognostic relevance of candidate genes was assessed via Kaplan–Meier survival curves, with statistical significance determined by the log-rank test (p < 0.05). For CCDC138, patients were divided into high and low-expression groups based on the median expression level. Differential expression of CCDC138 between tumor and normal tissues was validated using boxplots generated with the ggplot2 package.

### 2.7 CCDC138 expression and subcellular localization

The transcriptional expression of CCDC138 across 33 cancer types was analyzed by integrating data from TCGA and GTEx (Tang et al., 2017). Boxplots were generated using the ggplot2 package in R to visualize differential expression. Protein level expression in UCEC was evaluated using CPTAC data assessed via the UALCAN portal. Expression differences across tumor stages, histological grades, and molecular subtypes were compared, with statistical significance determined using the Wilcoxon rank-sum test (p < 0.05). Subcellular localization of CCDC138 was assessed using immunofluorescence data from The Human Protein Atlas (https://www.proteinatlas.org/). Staining patterns in A-431 (epidermoid carcinoma), U-251MG (glioma), and U-2 OS (osteosarcoma) cell lines were examined to evaluate co-localization with cellular components, including nuclei, endoplasmic reticulum, and microtubules.

### 2.8 Signaling pathway analysis

To elucidate signaling pathways associated with CCDC138, proteomic data from the CPTAC-UCEC cohort (99 tumor samples and 31 adjacent normal samples) were analyzed. Samples were grouped based on pathway activity status (“pathway-altered” vs. “other”), and differences in CDKN2A protein expression were assessed using the Wilcoxon rank-sum test. Key pathways analyzed included Hippo, NRF2, RTK, WNT, chromatin remodeling, mTOR, p53/Rb, and MYC/MYCN (Sanchez-Vega et al., 2018; Dou et al., 2020). Boxplots were generated using the ggplot2 package in R to visualize pathway-specific alterations.

### 2.9 Immune cell infiltration and TME analysis

The association between CCDC138 expression and immune cell infiltration was assessed using the CIBERSORT algorithm (version 1.03) and immune profiling data from the TIMER database (https://cistrome.shinyapps.io/timer/) (Newman et al., 2015; Li et al., 2016). Pearson correlation coefficients were computed for 68 immune cell types, with significance set at p < 0.05. Correlation patterns were visualized using bubble plots generated with ggplot2. TME components, including stromal and immune scores, were estimated using the ESTIMATE algorithm in R (version 1.0.13). Tumor purity was also calculated. Differences in TME metrics between high and low CCDC138 expression groups were evaluated using the Wilcoxon rank-sum test (p < 0.05).

### 2.10 Drug sensitivity analysis

To assess potential drug responses in TCGA-UCEC samples, TPM expression data were integrated with drug sensitivity profiles from the Genomics of Drug Sensitivity in Cancer database (Yang et al., 2013). Normal tissue samples and genes with low expression were excluded. The half-maximal inhibitory concentration (IC50) values for various therapeutic agents were predicted using a drug response modeling approach (Geeleher et al., 2014). Samples were categorized into high and low CCDC138 expression groups based on the median expression level. IC50 values were then compared between the two groups. Drugs showing significantly increased sensitivity in the high-expression groups (p < 0.001 and logFC <0) were visualized using boxplots.

### 2.11 Protein-protein interaction (PPI) network analysis

The PPI network of CCDC138 was constructed using the GeneMANIA database (https://genemania.org/). Interacting proteins were identified along with their interaction types (e.g., physical, genetic) and interaction strengths. The network was visualized in Cytoscape software (version 3.9.1), with nodes representing proteins and edges denoting the type and strength of the interactions (Shannon et al., 2003).

### 2.12 Cell culture and siRNA transfection

Ishikawa cells were cultured in RPMI-1640 medium (Hyclone, Cat.No.SH30809.01B) supplemented with 10% fetal bovine serum (FBS, Hyclone, Cat.No.SH30087.01) and penicillin-streptomycin (Hyclone, Cat.No.SH30010) at 37°C with 5% CO_2_. Cells were seeded at 5 × 10^4^ cells/well in 6-well plates. At 40% confluence, cells were transfected with siRNA targeting CCDC138 (si-CCDC138: ctcgactatgacatcaacattgadTdT) or negative control (si-NC: tcccgcgagacaacaccacctcadTdT) using Lipofectamine™ RNAiMAX (Invitrogen) in Opti-MEM. After 4–6 h, the transfection medium was replaced with complete medium. Transfection efficiency was assessed via RT-PCR after 24 h.

### 2.13 Cell proliferation assays

For CCK-8 assay, cells (1 × 10^4^/well) were seeded in 96-well plates. At 0, 24, 48, and 72 h, CellTiter96 AQueous One Solution (Promega, Cat.No.G3582) was added (10 µL/100 µL medium), incubated for 4 h, and absorbance measured at 490 nm using a microplate reader (Thermo Fisher Scientific, Multiscan MK3). Proliferation and inhibition rates were calculated.

For EdU assay, log-phase cells were seeded in 96-well plates, incubated with EdU (10 µM) for 2 h, fixed with 4% paraformaldehyde, and stained using an EdU kit. Nuclei were counterstained with DAPI. Images were captured using a Leica DMI6000B fluorescence microscope, and EdU-positive cells were quantified.

### 2.14 RT-PCR analysis

Total RNA was extracted using TRIzol (Invitrogen), treated with DNase I (Promega), and reverse-transcribed. RT-PCR was performed using SYBR Green qPCR SuperMix (Invitrogen) on an ABI PRISM® 7500 system with primers for CCDC138, S6K1, mTOR, p21, and GAPDH (internal control). Relative expression was calculated using the 2^−ΔΔCT^ method.

### 2.15 Statistical analysis

All statistical analyses were performed in R (version 4.2.1), with parallel processing enabled via the future.apply and doParallel packages. Continuous variables were analyzed using the Wilcoxon rank-sum test or t-test, as appropriate. Survival analysis was conducted using the log-rank test. For multiple comparisons, p-values were adjusted using the FDR method. Unless otherwise specified, p-values <0.05 were considered statistically significant.

## 3 Results

### 3.1 Data preprocessing and expression matrix construction

RNA sequencing data from the TCGA-UCEC cohort were preprocessed to generate normalized expression matrices. After quality control and filtering, 584 samples and 60,616 genes were retained. Expression values were converted to the TPM matrix and subsequently log-transformed using the formula log2 (TPM +1). Genes with low-expression (mean TPM <1) were excluded, establishing a high-quality dataset for downstream analyses.

### 3.2 ssGSEA and clustering

**FIGURE 1:**
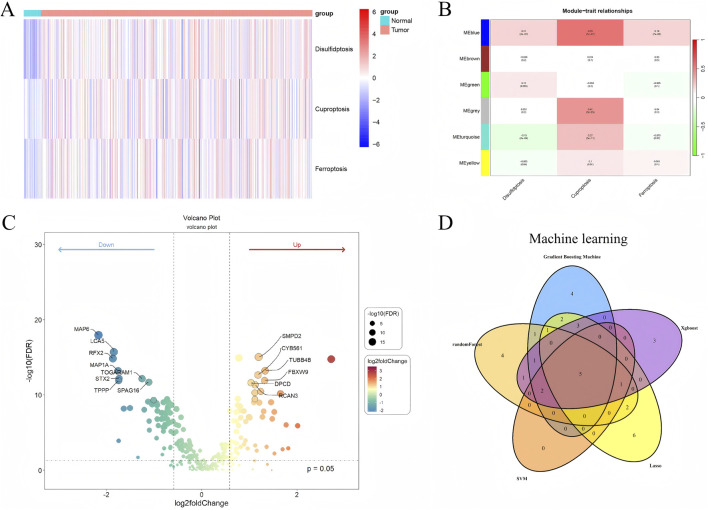
Molecular and genomic analyses of UCEC samples **(A)** Heatmap of ssGSEA enrichment scores for disulfidptosis, cuproptosis, and ferroptosis pathways, illustrating distinct separation between tumor and normal samples, red indicates higher enrichment scores, blue indicates lower scores. **(B)** WGCNA module-trait correlation heatmap, highlighting the blue module’s significant association with tumor traits. **(C)** Volcano plot of differentially expressed genes (DEGs) in the blue module, with significant DEGs (|log2FC| > 1, FDR <0.05) annotated. **(D)** Venn diagram depicting five overlapping feature genes (*MAP6*, *CCDC138*, *DNAAF3*, *STX2*, *FABP6*) identified by five machine learning algorithms (GBM, RF, SVM-RFE, Lasso, XGBoost).

ssGSEA was performed to calculate enrichment scores for disulfidptosis, cuproptosis, and ferroptosis pathways. The resulting heatmap (Figure 1A) demonstrated distinct separation between tumor and normal samples, indicating pronounced molecular differences.

### 3.3 WGCNA

WGCNA identified six distinct co-expression modules using a soft-thresholding power of β = 16 (*R* ^2^ > 0.9). After removing outliers, 584 samples were retained for analysis. The blue module, containing 1,800 genes, showed the strongest correlation with tumor traits and was selected for subsequent analysis (Figure 1B).

### 3.4 Differential expression analysis

A total of 46 DEGs were identified within the blue module (|log2FC| > 1, FDR <0.05), comprising 28 upregulated and 18 downregulated genes. Significant DEGs were visualized using a volcano plot (Figure 1C), and expression differences were further validated through boxplots (Supplementary Material, containing boxplots of DEG expression). The resulting DEG expression matrix was retained for downstream analyses.

### 3.5 Machine learning-based feature selection

Five machine learning algorithms were applied to identify feature genes, yielding 19 from GBM, 18 from RF, eight from SVM-RFE, 20 from Lasso, and 16 from XGBoost. A Venn diagram revealed five overlapping genes—*MAP6*, *CCDC138*, *DNAAF3*, *STX2*, and *FABP6* —which were selected for downstream analyses based on their consensus across models expression profiles (Figure 1D).

### 3.6 Survival analysis

**FIGURE 2:**
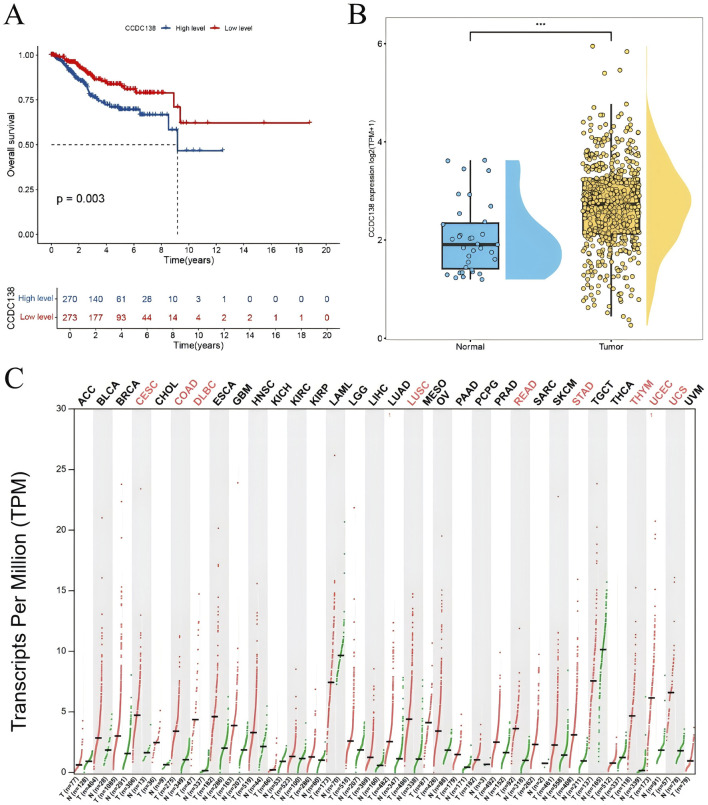
Survival and expression analysis of CCDC138 in UCEC **(A)** Kaplan–Meier survival curve showing poorer overall survival (OS) in the CCDC138 high-expression group (p = 0.003). **(B)** Boxplot validates significantly higher CCDC138 expression in UCEC tumor tissues than in normal tissues. **(C)** Bar plot of CCDC138 transcriptional levels across 33 cancer types from TCGA and GTEx databases, with significant upregulation in UCEC.

Survival analysis identified four genes significantly associated with OS (p < 0.05). Among them, high expression of CCDC138 was significantly correlated with poorer OS (p = 0.003, Figure 2A). Differential expression analysis further revealed that CCDC138 expression was significantly elevated in UCEC tumor tissues compared to that in normal tissues (Figure 2B), highlighting its potential as a prognostic biomarker.

### 3.7 CCDC138 expression analysis

**FIGURE 3:**
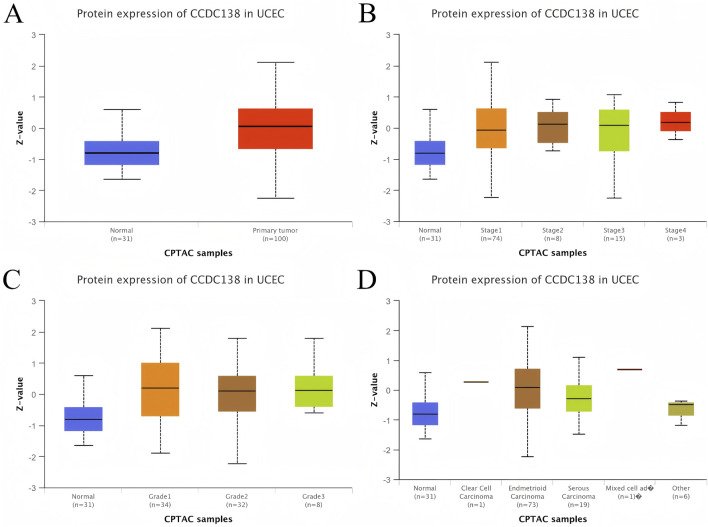
CCDC138 protein expression in UCEC **(A)** Boxplot of CCDC138 protein expression in UCEC (100 samples) versus normal samples (31 samples) from the CPTAC dataset, showing significant upregulation in UCEC. **(B)** Boxplot of CCDC138 protein expression across different UCEC stages, indicating significant upregulation in early stages. **(C)** Boxplot of CCDC138 protein expression across UCEC grades, showing significant differences. **(D)** Boxplot of CCDC138 protein expression across UCEC histological subtypes, highlighting subtype-specific upregulation.

To assess the biomarker potential of CCDC138 in UCEC, its transcriptional expression was analyzed across 33 cancer types using data from TCGA and GTEx databases (Figure 2C). CCDC138 was significantly upregulated in several cancers, including UCEC. Protein-level analysis using the CPTAC dataset via the UALCAN portal further confirmed elevated CCDC138 expression in UCEC tumor tissues compared to that in normal controls (31 normal vs. 100 tumor samples, Figure 3A). Stratified analysis revealed significantly higher protein expression across different UCEC stages (Figure 3B), histological grades (Figure 3C), and subtypes (Figure 3D), with marked elevation in early-stage disease and specific subtypes.

### 3.8 Subcellular localization of CCDC138

**FIGURE 4:**
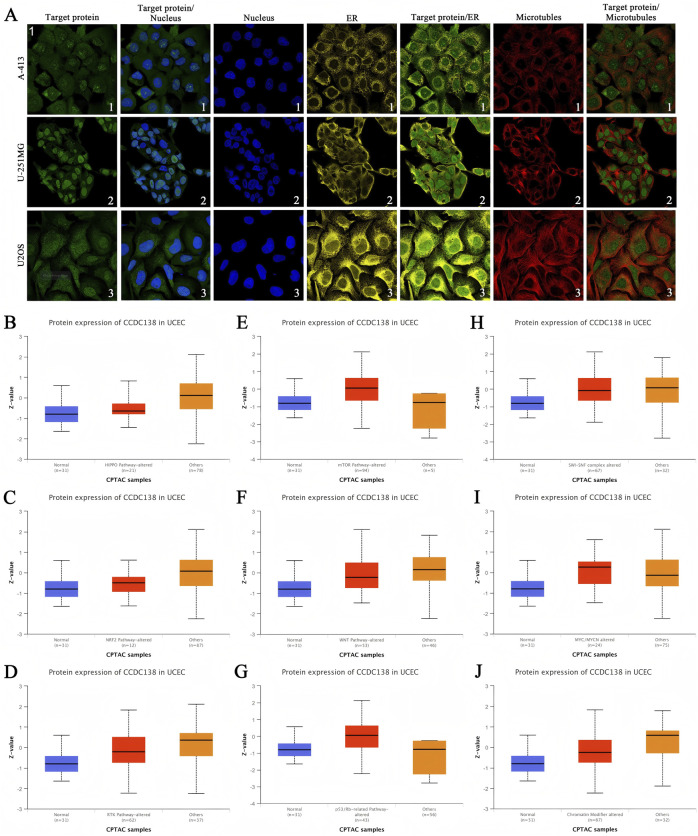
Subcellular localization and signaling pathway analysis of CCDC138 **(A)** Immunofluorescence images showing CCDC138 subcellular distribution in A-431, U-251MG, and U-2 OS cell lines, co-localized with DAPI-stained nuclei, endoplasmic reticulum, and microtubules. **(B–J)** Boxplots comparing CCDC138 protein expression in CPTAC-UCEC samples (99 cases) with pathway-altered versus other groups, relative to 31 normal adjacent samples, revealing associations with Hippo, NRF2, RTK, WNT, chromatin modifiers, mTOR, p53/Rb, and MYC/MYCN pathways.

Immunofluorescence analysis of A-431 (epidermoid carcinoma), U-251MG (glioma), and U-2 OS (osteosarcoma) cell lines, as reported in the THPA, was used to investigate the subcellular distribution of CCDC138 (Figure 4A). CCDC138 exhibited co-localization with DAPI-stained nuclei and was detected in both the nuclear and cytoplasmic compartments in all examined cell lines.

### 3.9 Signaling pathway analysis

To investigate the involvement of CCDC138 in UCEC pathogenesis, proteomic data from the CPTAC-UCEC cohort (99 tumor samples and 31 adjacent normal tissues) were analyzed. Samples were categorized into “pathway-altered” and “other” groups for key oncogenic pathways. Comparative analysis revealed significantly elevated CCDC138 protein expression in samples with alterations in the Hippo, NRF2, RTK, WNT, and chromatin remodeling pathways. In addition, CCDC138 expression was strongly associated with dysregulations of the mTOR, p53/Rb, and MYC/MYCN pathways (Figures 4B–J).

### 3.10 Immune cell infiltration correlation

**FIGURE 5:**
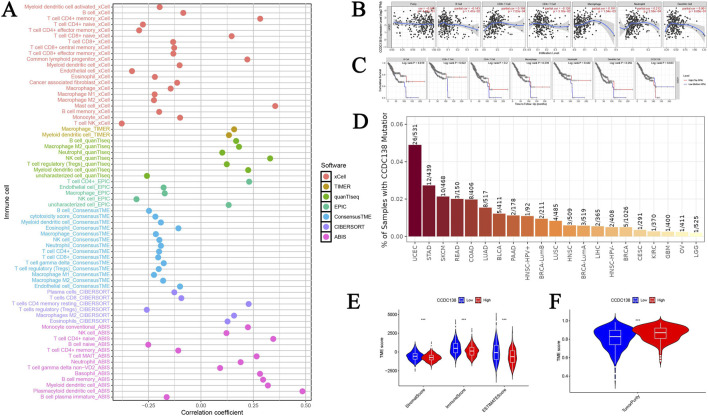
Immune microenvironment and TME analysis of CCDC138 **(A)** Bubble plot illustrating correlations between CCDC138 expression and 68 immune cell types, highlighting significant positive (e.g., plasmacytoid dendritic cells) and negative (e.g., NKT cells) correlations. **(B)** A scatter plot from the TIMER database shows negative correlations between CCDC138 expression and immune cell activity. **(C)** Kaplan–Meier curve demonstrating significant associations between CCDC138 expression and UCEC patient survival. **(D)** Bar plot of CCDC138 mutation frequency across TCGA cancers, with the highest frequency in UCEC. **(E)** Boxplot showing significantly lower stromal and immune scores in the CCDC138 high-expression group. **(F)** Boxplot indicating higher tumor purity in the CCDC138 high-expression group.

CCDC138 expression was significantly correlated with 68 immune cell types (p < 0.05), including a strong positive correlation with plasmacytoid dendritic cells (r = 0.484, p = 1.20e-33) and a negative correlation with natural killer T (NKT) cells (r = −0.378, p = 4.43e-20). A bubble plot illustrated the overall correlation patterns (Figure 5A), suggesting a potential role for CCDC138 in modulating the immune microenvironment. Further analysis using the TIMER database confirmed negative associations between CCDC138 expression and immune cell infiltration (Figure 5B), significant links with OS in patients with UCEC (Figure 5C), and the highest CCDC138 mutation frequency among TCGA cancer types (Figure 5D).

### 3.11 TME analysis

The high CCDC138 expression group exhibited significantly lower stromal scores (p = 3.92e-07) and immune scores (p = 1.41e-08; Figure 5E), with higher tumor purity (p = 1.88e-10; Figure 5F), indicating that CCDC138 may influence the TME and contribute to tumor progression.

### 3.12 Drug sensitivity prediction

Drug sensitivity analysis identified 81 compounds with significantly lower IC50 values in the high CCDC138 expression group (p < 0.001, logFC <0), indicating enhanced drug sensitivity. Boxplots for agents, such as 5-fluorouracil, acetalax, alpelisib, AZ6102, and AZD3759 (Supplementary Material, containing drug sensitivity boxplots) highlighted the potential of CCDC138 as a predictive biomarker for treatment response.

### 3.13 PPI network

**FIGURE 6:**
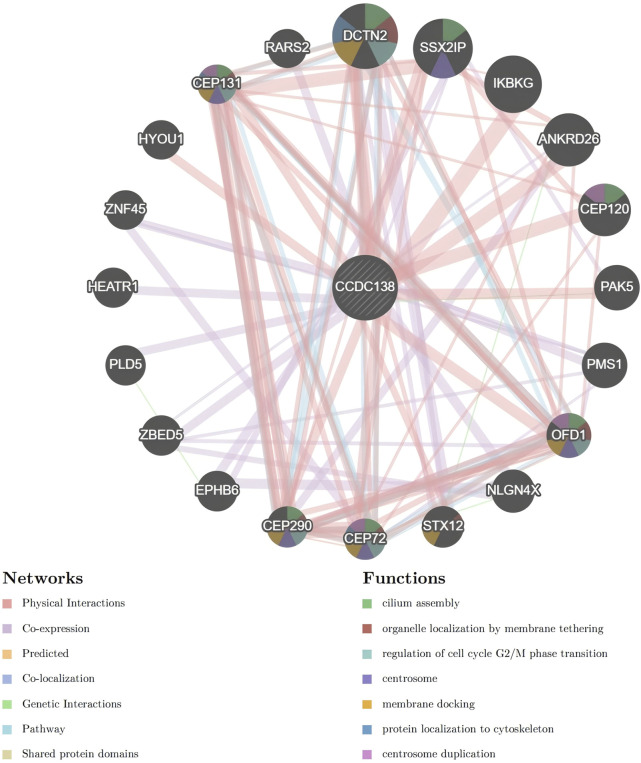
Protein-protein interaction network of CCDC138 Network diagram generated from the GeneMania database, illustrating interactions between CCDC138 and proteins, including DCTN2, SSX2IP, CEP72, CEP290, CEP131, CEP120, OFD1, STX12, PMS1, PAK5, IKBKG, ANKRD26, RARS2, HYOU1, ZNF45, HEATR1, PLD5, ZBED5, EPHB6, and NLGN4X, with interaction types and strengths detailed in Table 1.

PPI analysis using the GeneMania database identified multiple CCDC138-interacting partners, including DCTN2, SSX2IP, CEP72, CEP290, CEP131, CEP120, OFD1, STX12, PMS1, PAK5, IKBKG, ANKRD26, RARS2, HYOU1, ZNF45, HEATR1, PLD5, ZBED5, EPHB6, and NLGN4X. Interaction types and strengths are summarized in Table 1 and illustrated in a network diagram (Figure 6).

**TABLE 1:** Protein-protein interactions of CCDC138.

Entity 1	Entity 2	Weight	Network group
PMS1	CCDC138	0.025178	Co-expression
ZBED5	CCDC138	0.026149	Co-expression
HEATR1	CCDC138	0.025981	Co-expression
ZNF45	CCDC138	0.025609	Co-expression
RARS2	CCDC138	0.024698	Co-expression
NLGN4X	CCDC138	0.03203	Co-expression
EPHB6	CCDC138	0.029218	Co-expression
PLD5	CCDC138	0.028058	Co-expression
SSX2IP	CCDC138	0.006313	Co-expression
PMS1	CCDC138	0.013945	Co-expression
PAK5	CCDC138	0.002528	Genetic Interactions
ANKRD26	CCDC138	0.09482	Physical Interactions
CEP120	CCDC138	0.090043	Physical Interactions
OFD1	CCDC138	0.071572	Physical Interactions
CEP72	CCDC138	0.0588	Physical Interactions
CEP131	CCDC138	0.053746	Physical Interactions
IKBKG	CCDC138	0.133863	Physical Interactions
SSX2IP	CCDC138	0.125896	Physical Interactions
CEP290	CCDC138	0.071661	Physical Interactions
DCTN2	CCDC138	0.20366	Physical Interactions
PAK5	CCDC138	0.168674	Physical Interactions
STX12	CCDC138	0.092275	Physical Interactions
DCTN2	CCDC138	0.254645	Physical Interactions
STX12	CCDC138	0.104813	Physical Interactions
HYOU1	CCDC138	0.311397	Physical Interactions

### 3.14 *In Vitro* validation of CCDC138 function

To validate the bioinformatics findings, *in vitro* experiments were conducted using Ishikawa cells, a well-established UCEC model.

#### 3.14.1 siRNA knockdown efficiency

**FIGURE 7:**
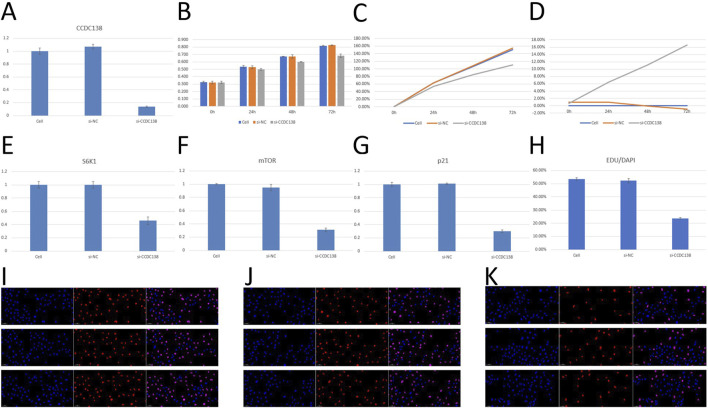
*In vitro* validation of CCDC138 function in Ishikawa cells **(A)** RT-PCR analysis showing significant reduction in CCDC138 mRNA expression in si-CCDC138 cells (p < 0.05). **(B)** CCK-8 assay results showing reduced proliferation in si-CCDC138 cells compared to that in the control and si-NC groups. **(C)** Proliferation rates calculated from CCK-8 assay data, highlighting lower proliferation in si-CCDC138 cells at 24 h, 48 h, and 72 h (p < 0.05 at 48 h and 72 h). **(D)** Inhibition rates from CCK-8 assay, showing increasing inhibition over time in si-CCDC138 cells (p < 0.05 at 48 h and 72 h). **(E–G)** RT-PCR analysis of S6K1, mTOR, and p21 mRNA expression, showing significant downregulation post-CCDC138 knockdown (p < 0.05). **(H)** EdU assay quantification showing reduced proliferation in si-CCDC138 cells (p < 0.05). **(I–K)** Fluorescence microscopy images of EdU assays for control, si-NC, and si-CCDC138 groups, respectively, visually confirming fewer proliferating cells in the si-CCDC138 group.

RT-PCR confirmed effective CCDC138 knockdown in Ishikawa cells transfected with si-CCDC138 (2^−ΔΔCT^ = 0.14 ± 0.01) compared to control (2^−ΔΔCT^ = 1.00 ± 0.05) and si-NC (2^−ΔΔCT^ = 1.07 ± 0.04; p < 0.05; Table 2; Figure 7A). The significant reduction (86% decrease) in CCDC138 mRNA expression validated the efficacy of the siRNA construct.

**TABLE 2:** RT-PCR detection of siRNA knockdown efficiency.

Sample-gene	Repeat 1 Ct value	Repeat 2 Ct value	Repeat 2 Ct value	Ct average	ΔCT	ΔΔCT	2^−ΔΔCT^
Cell-CCDC138	25.19	25.06	25.11	25.12 ± 0.07	14.95 ± 0.07	0.00 ± 0.07	1.00 ± 0.05
si-NC-CCDC138	25.04	25.05	25.13	25.07 ± 0.05	14.84 ± 0.05	−0.10 ± 0.05	1.07 ± 0.04
si-CCDC138-CCDC138	28.01	28.06	27.86	27.98 ± 0.10	17.80 ± 0.10	2.85 ± 0.10	0.14 ± 0.01
Cell-GAPDH	10.21	10.17	10.14	10.17 ± 0.04	——	——	——
si-NC-GAPDH	10.3	10.27	10.11	10.23 ± 0.10	——	——	——
si-CCDC138-GAPDH	10.24	10.21	10.1	10.18 ± 0.07	——	——	——

#### 3.14.2 Cell proliferation assays

CCK-8 assay was used to assess cell proliferation at 0, 24, 48, and 72 h post-transfection (Table 3; Figure 7B). The si-CCDC138 group showed significantly reduced proliferation compared to those of the control and si-NC groups. Proliferation rates were 53.44% (si-CCDC138) vs. 62.90% (control) at 24 h, 84.51% vs. 106.32% at 48 h, and 110.26% vs. 150.36% at 72 h (Table 4; Figure 7C). Inhibition rates increased over time, reaching 6.38% at 24 h, 11.12% at 48 h, and 16.53% at 72 h (Table 5; Figure 7D). These results indicate that CCDC138 promotes cell proliferation in UCEC.

**TABLE 3:** CCK-8 assay for cell proliferation.

Time	Sample	OD1	OD2	OD3	Mean	Std
0 h	Cell	0.313	0.346	0.322	0.327	0.014
	si-NC	0.328	0.301	0.343	0.324	0.017
	si-CCDC138	0.346	0.317	0.312	0.325	0.015
24 h	Cell	0.531	0.513	0.554	0.533	0.017
	si-NC	0.529	0.502	0.551	0.527	0.020
	si-CCDC138	0.506	0.479	0.511	0.499	0.014
48 h	Cell	0.677	0.676	0.671	0.675	0.003
	si-NC	0.643	0.695	0.687	0.675	0.023
	si-CCDC138	0.601	0.603	0.595	0.600	0.003
72 h	Cell	0.816	0.813	0.827	0.819	0.006
	si-NC	0.826	0.833	0.820	0.826	0.005
	si-CCDC138	0.651	0.691	0.708	0.683	0.024

**TABLE 4:** Proliferation rate calculation.

Sample	0 h	24 h	48 h	72 h
Cell	0.00%	62.90%	106.32%	150.36%
si-NC	0.00%	62.76%	108.33%	155.04%
si-CCDC138	0.00%	53.44%	84.51%	110.26%

**TABLE 5:** Inhibition rate calculation.

Sample	0 h	24 h	48 h	72 h
Cell	0.00%	0.00%	0.00%	0.00%
si-NC	0.92%	1.00%	−0.05%	−0.94%
si-CCDC138	0.61%	6.38%	11.12%	16.53%

The EdU incorporation assay further confirmed reduced proliferation in the si-CCDC138 group. The percentage of EdU-positive cells (EdU/DAPI) was significantly lower in si-CCDC138 (23.86% ± 0.63%) than in the control (53.43% ± 1.01%) and si-NC (52.26% ± 1.68%; p < 0.05; Table 6; Figure 7H). Fluorescence microscopy images (Figures 7I–K) visually demonstrated fewer proliferating cells in the si-CCDC138 group, corroborating CCK-8 findings and highlighting CCDC138’s role in driving UCEC cell growth.

**TABLE 6:** EdU assay cell counts.

Sample	DAPI			EdU			EDU/DAPI			Mean	Std
Cell	80	85	93	42	45	51	52.50%	52.94%	54.84%	53.43%	1.01%
si-NC	86	87	91	43	47	48	50.00%	54.02%	52.75%	52.26%	1.68%
si-CCDC138	87	87	90	21	20	22	24.14%	22.99%	24.44%	23.86%	0.63%

#### 3.14.3 RT-PCR analysis of oncogenic pathways

RT-PCR was used to assess the impact of CCDC138 knockdown on mTOR, S6K1, and p21 mRNA expression, key components of oncogenic pathways identified by bioinformatics analyses (Table 7). In the si-CCDC138 group, mRNA expression was significantly reduced: S6K1 (2^−ΔΔCT^ = 0.46 ± 0.06 vs. 1.00 ± 0.05 in control, p < 0.05; Figure 7E), mTOR (2^−ΔΔCT^ = 0.31 ± 0.03 vs. 1.00 ± 0.01, p < 0.05; Figure 7F), and p21 (2^−ΔΔCT^ = 0.30 ± 0.02 vs. 1.00 ± 0.03, p < 0.05; Figure 7G). The si-NC group showed no significant changes compared to those in the controls (p > 0.05). These findings confirm that CCDC138 regulates key oncogenic signaling pathways, consistent with bioinformatics predictions.

**TABLE 7:** RT-PCR analysis of gene expression.

Sample-gene	Repeat 1 Ct value	Repeat 2 Ct value	Repeat 3 Ct value	Ct average	DCT	DDCT	2^−DDCT^
Cell-S6K1	25.73	25.62	25.73	25.69 ± 0.06	14.39 ± 0.06	0.00 ± 0.06	1.00 ± 0.05
si-NC-S6K1	25.78	25.67	25.63	25.69 ± 0.08	14.40 ± 0.08	0.00 ± 0.08	1.00 ± 0.05
si-CCDC138-S6K1	26.81	26.97	26.62	26.80 ± 0.18	15.50 ± 0.18	1.11 ± 0.18	0.46 ± 0.06
Cell-mTOR	26.17	26.19	26.15	26.17 ± 0.02	14.87 ± 0.02	0.00 ± 0.02	1.00 ± 0.01
si-NC-mTOR	26.17	26.28	26.28	26.24 ± 0.06	14.95 ± 0.06	0.08 ± 0.06	0.95 ± 0.05
si-CCDC138-mTOR	27.81	27.99	27.76	27.85 ± 0.12	16.55 ± 0.12	1.68 ± 0.12	0.31 ± 0.03
Cell-p21	26.54	26.47	26.49	26.50 ± 0.04	15.20 ± 0.04	0.00 ± 0.04	1.00 ± 0.03
si-NC-p21	26.47	26.46	26.5	26.48 ± 0.02	15.19 ± 0.02	−0.01 ± 0.02	1.01 ± 0.01
si-CCDC138-p21	28.16	28.27	28.33	28.25 ± 0.09	16.95 ± 0.09	1.75 ± 0.09	0.30 ± 0.02
Cell-GAPDH	11.26	11.31	11.33	11.30 ± 0.04	——	——	——
si-NC-GAPDH	11.27	11.27	11.34	11.29 ± 0.04	——	——	——
si-CCDC138-GAPDH	11.31	11.32	11.26	11.30 ± 0.03	——	——	——

## 4 Discussion

In this study, we comprehensively examined the role of CCDC138 in UCEC using integrative bioinformatics and multi-omics data from public repositories, including TCGA and CPTAC, complemented by *in vitro* experiments in Ishikawa cells. Our key findings revealed that CCDC138 is significantly upregulated at the mRNA and protein levels in UCEC and is associated with poor OS. Moreover, its expression correlates with major cancer-related pathways—such as mTOR, p53/Rb, and MYC/MYCN—and is significantly linked to immune infiltration and TME characteristics. High CCDC138 expression was associated with increased sensitivity to various chemotherapeutic agents. *In vitro* experiments validated these findings, demonstrating that CCDC138 knockdown in Ishikawa cells achieved an 86% reduction in mRNA expression (2^−ΔΔCT^ = 0.14 ± 0.01 vs. 1.00 ± 0.05 in control, p < 0.05), significantly reduced cell proliferation (CCK-8 assay: 16.53% inhibition at 72 h; EdU assay: 23.86% ± 0.63% EdU/DAPI vs. 53.43% ± 1.01% in control, p < 0.05,), and downregulated mTOR (2^−ΔΔCT^ = 0.31 ± 0.03), S6K1 (2^−ΔΔCT^ = 0.46 ± 0.06), and p21 (2^−ΔΔCT^ = 0.30 ± 0.02) mRNA expression (p < 0.05), key components of the mTOR and p53/Rb pathways, confirming its oncogenic role. These findings suggest that CCDC138 may serve as a promising biomarker and therapeutic target in UCEC, highlighting its unique dual relevance in ciliopathies and cancer.

The overexpression of CCDC138 across UCEC subtypes, stages, and grades suggests its involvement in tumor development and progression. The upregulation was observed at both transcriptional and protein levels and was significantly associated with adverse survival outcomes, underscoring its potential as a prognostic biomarker. *In vitro* validation further supported this, as CCDC138 knockdown significantly reduced proliferation rates (53.44% vs. 62.90% at 24 h, 84.51% vs. 106.32% at 48 h, 110.26% vs. 150.36% at 72 h; Figure 7C), indicating a direct role in driving tumor cell growth. While CCDC138 has been reported as overexpressed in other malignancies per THPA, its functional relevance in UCEC has not been previously characterized (Uhlén et al., 2015). This study is the first to delineate the expression profile of CCDC138 in UCEC and to establish its clinical relevance, laying a groundwork for future diagnostic and therapeutic strategies.

As a ciliopathy-associated gene, CCDC138 may influence the structure and function of primary cilia—organelles essential for signal transduction, cellular differentiation, proliferation, and tissue homeostasis (Anvarian et al., 2019). While cilia sustain tumorigenic signaling in cancers, such as medulloblastoma and basal cell carcinoma via hedgehog signaling, UCEC is not typically driven by hedgehog signaling (Hassounah et al., 2012). Instead, CCDC138 may exert its effects through alternative cilia-dependent pathways (Wnt or PDGF) or non-ciliary mechanisms. Subcellular localization analysis revealed that CCDC138 is present in nuclear and cytoplasmic compartments, suggesting potential roles in transcriptional regulation or chromatin organization (Liu et al., 2023). The *in vitro* findings, showing reduced proliferation and altered gene expression post-knockdown, support the hypothesis that CCDC138 may regulate cellular processes through both ciliary and non-ciliary mechanisms, warranting further mechanistic exploration. Pathway analysis showed that CCDC138 is associated with the mTOR, p53/Rb, and MYC/MYCN signaling pathways, all of which are crucial in UCEC pathogenesis. The mTOR pathway is a known therapeutic target due to its role in cellular growth, metabolism, and survival (Saxton and Sabatini, 2017). *In vitro* experiments confirmed that CCDC138 knockdown significantly reduced mTOR (69% reduction) and S6K1 (54% reduction) mRNA expression, validating the bioinformatics association with the mTOR pathway and suggesting that CCDC138 directly or indirectly modulates this pathway to promote tumor growth. p53 and Rb are tumor suppressors, and their inactivation drives uncontrolled proliferation (Zhou et al., 2023); the observed downregulation of p21 (70% reduction) post-knockdown indicates that CCDC138 may inhibit p53/Rb-mediated cell cycle arrest. MYC governs the expression of genes involved in cell cycle progression (Stine et al., 2015). The associations between CCDC138 and these signaling cascades suggest that CCDC138 may modulate oncogenic signaling either directly or indirectly. Its nuclear localization raises the possibility of transcriptional regulation, while potential cilia-dependent mechanisms might influence upstream signaling dynamics. The *in vitro* reduction in proliferation further supports these pathway associations, as it aligns with the expected outcomes of disrupted mTOR and p53/Rb signaling.

The results also showed that high CCDC138 expression was associated with lower immune and stromal scores, and higher tumor purity, indicative of an immunosuppressive TME. This “cold” TME phenotype often correlates with poor responses to immunotherapy and unfavorable prognosis (Wu et al., 2024). CCDC138 expression demonstrated both positive and negative correlations with specific immune cell populations, including a notable positive correlation with plasmacytoid dendritic cells and a negative correlation with NKT cells. These findings suggest that CCDC138 may modulate immune evasion mechanisms by altering immune cell recruitment or function. The *in vitro* data, while focused on proliferation and pathway regulation, indirectly support the idea of an altered TME, as reduced proliferation may reflect changes in tumor cell behavior that influence immune interactions. Further studies should examine whether CCDC138 regulates immune checkpoint expression or cytokine signaling.

Drug sensitivity analysis revealed that high CCDC138 expression conferred increased sensitivity to 81 therapeutic agents, including 5-fluorouracil and alpelisib. These findings suggest that CCDC138 may serve as a predictive biomarker for treatment response in UCEC. For example, alpelisib targets the PI3K pathway and has shown clinical efficacy in various cancers (Mohankumar et al., 2015; Mohankumar et al., 2014a; Mohankumar et al., 2014b); CCDC138 expression may help identify patients with UCEC likely to benefit from such targeted therapies (Chang et al., 2021). The *in vitro* downregulation of mTOR, a pathway closely linked to PI3K, supports the potential efficacy of alpelisib in high-CCDC138 tumors. However, these results are based on computational predictions and require validation in clinical trials.

To further investigate the molecular functions of CCDC138, we constructed a PPI network, identifying patterns involved in centrosome function, cilia formation, and cell cycle regulation. Notable interactors included DCTN2, SSX2IP, CEP72, CEP290, CEP131, and CEP120, proteins central to microtubule dynamics and ciliogenesis. For instance, DCTN2 plays a vital role in mitotic spindle assembly and has been implicated in hepatocellular carcinoma via the AKT pathway (Li et al., 2022; Ch et al., 2015; Chaudhuri et al., 2018). SSX2IP contributes to centrosome maturation (Hori et al., 2015) and is overexpressed in leukemia. CEP290 regulates the ciliary transition zone and has roles in both Joubert syndrome and oncogenic signaling (Shimada et al., 2017; Stowe et al., 2012; Lüddecke et al., 2016). CEP131 is involved in centriole duplication and genome integrity (Denu et al., 2019). CEP120 facilitates centriole elongation in coordination with SPICE1 (Comartin et al., 2013). Therefore, through its interactions with ciliary proteins, CCDC138 may regulate centrosome integrity, ciliary assembly, and signaling pathways, such as hedgehog and Wnt. The *in vitro* findings, particularly the reduced proliferation post-knockdown, suggest that these interactions may contribute to tumor cell growth, potentially through disrupted ciliary or centrosomal functions. In UCEC, these interactions may lead to cilia dysfunction, promoting tumorigenesis, while in ciliopathies, disrupted centriole replication or basal body formation may result in structural ciliary defects. As ciliary dysfunction is a common feature in both cancer and ciliopathies, CCDC138 may contribute to disease progression by modulating cilia-dependent pathways, including mTOR and p53/Rb, thereby influencing the TME and cell cycle. Through these interactions, CCDC138 may influence both cancer progression and ciliary dysfunction, reinforcing its relevance in both disease contexts.

Based on these findings, we propose that CCDC138 may exert its effects in UCEC through several mechanisms: (1) regulating ciliary assembly and signaling via interaction with transition zone proteins, such as CEP290; (2) modulating microtubule-mediated transport through interaction with DCTN2; and (3) affecting cell cycle progression and centrosome dynamics via interaction with centrosomal proteins. These mechanisms may contribute to both tumor proliferation and ciliary abnormalities observed in related disorders.

Despite these insights, this study has several limitations. First, the analyses were conducted using public datasets without experimental validation, limiting the ability to establish causality. Second, the drug sensitivity predictions have not been confirmed in clinical or laboratory models. Third, the study did not directly assess the role of CCDC138 in ciliary function, which would further clarify its role in ciliopathies and tumor biology. Future *in vitro* and *in vivo* studies are essential to validate these findings and fully elucidate the mechanistic roles of CCDC138 in UCEC.

## 5 Conclusion

This study is the first to systematically characterize the expression profile of CCDC138 in UCEC and to evaluate its prognostic relevance and associations with key oncogenic pathways, the immune microenvironment, and drug sensitivity. As a gene implicated in ciliopathies and UCEC, CCDC138 offers a novel perspective for exploring the molecular link between ciliary dysfunction and cancer. Its overexpression is associated with poor prognosis, activation of oncogenic pathways, and an immunosuppressive TME, highlighting its potential as a diagnostic, prognostic, and predictive biomarker. *In vitro* experiments confirmed that CCDC138 knockdown inhibits proliferation and downregulates mTOR, S6K1, and p21, validating its oncogenic role. As a ciliopathy-associated gene, CCDC138 bridges ciliary dysfunction and cancer, offering a novel biomarker and therapeutic target for precision oncology in UCEC.

## Data Availability

The datasets presented in this study can be found in online repositories. The names of the repository/repositories and accession number(s) can be found in the article/Supplementary Material.
