# Supplementary figures and images for: CCDC138 overexpression predicts poor prognosis and highlights ciliopathy-linked mechanisms in uterine corpus endometrial carcinoma (part 2 of 2)
Source: Front Mol Biosci. 2025 Aug 8;12:1622496. doi: 10.3389/fmolb.2025.1622496 (PMC12370488; doi:10.3389/fmolb.2025.1622496)

CCDC138 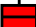 High 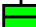 Low

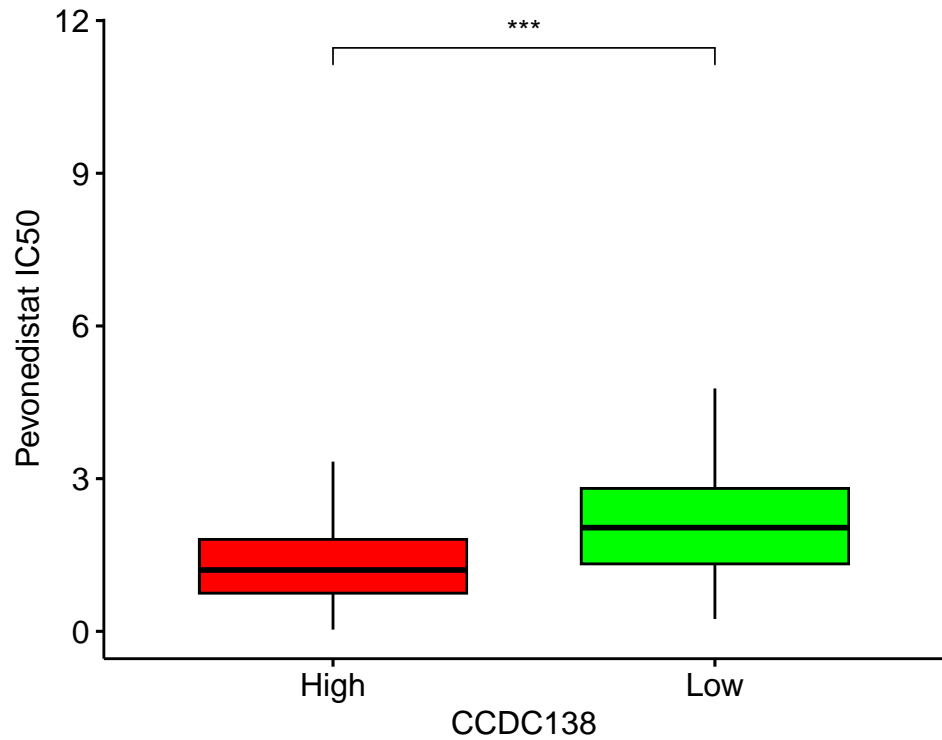

Supplement: Supplementary file 1 [file DataSheet1.zip › supplementary file/supplementary file 2/CCDC138_drugSenstivity.Pevonedistat.pdf]

CCDC138 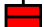 High 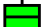 Low

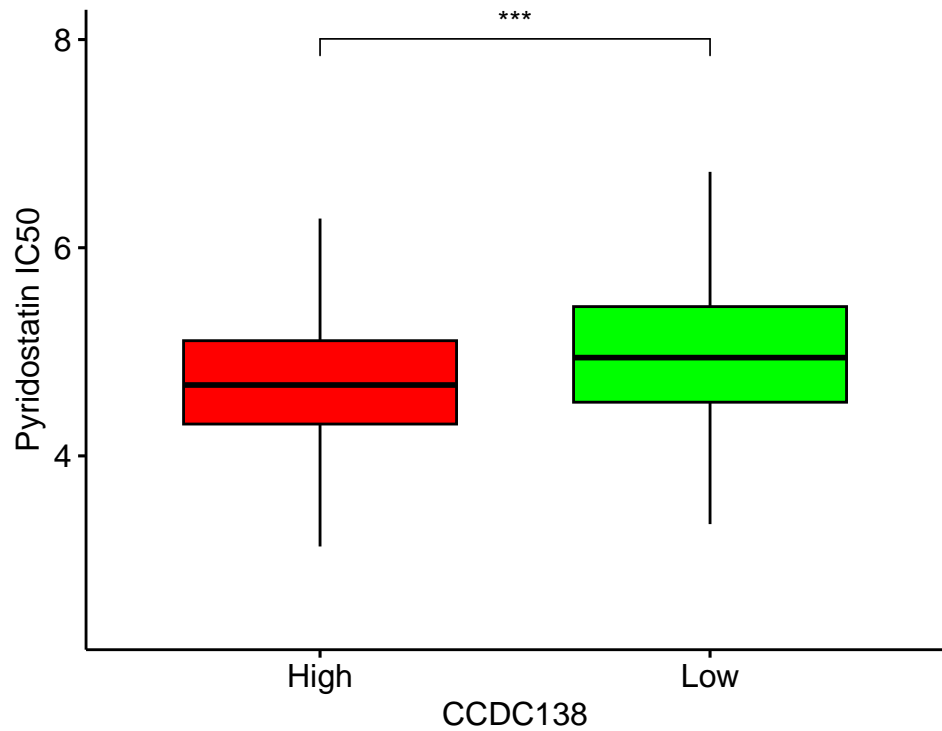

Supplement: Supplementary file 1 [file DataSheet1.zip › supplementary file/supplementary file 2/CCDC138_drugSenstivity.Pyridostatin.pdf]

CCDC138 High Low

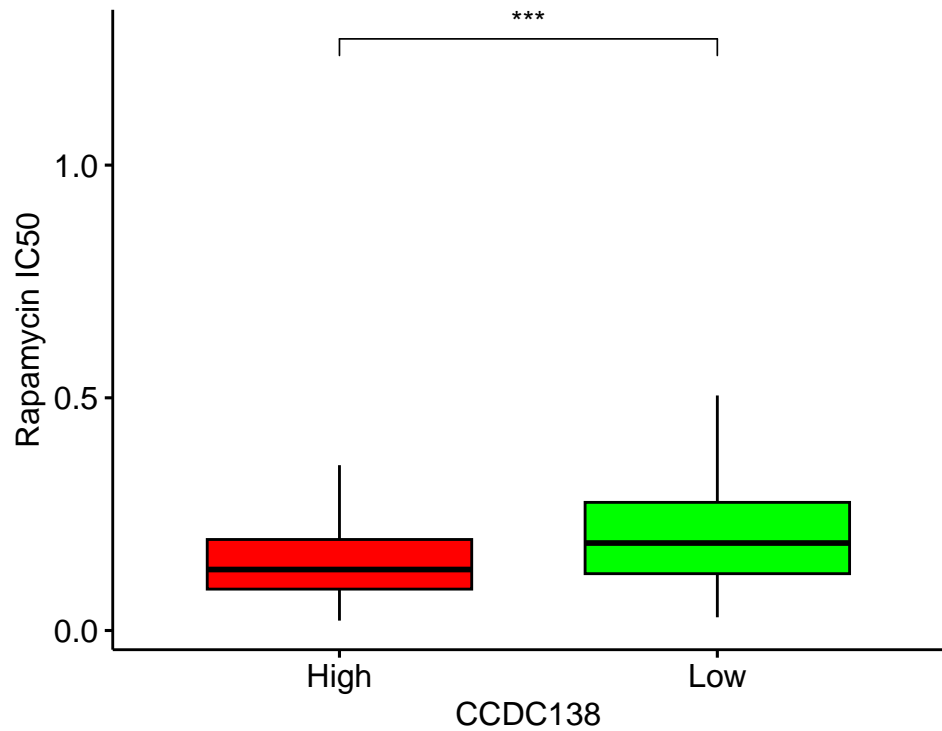

Supplement: Supplementary file 1 [file DataSheet1.zip › supplementary file/supplementary file 2/CCDC138_drugSenstivity.Rapamycin.pdf]

CCDC138 High Low

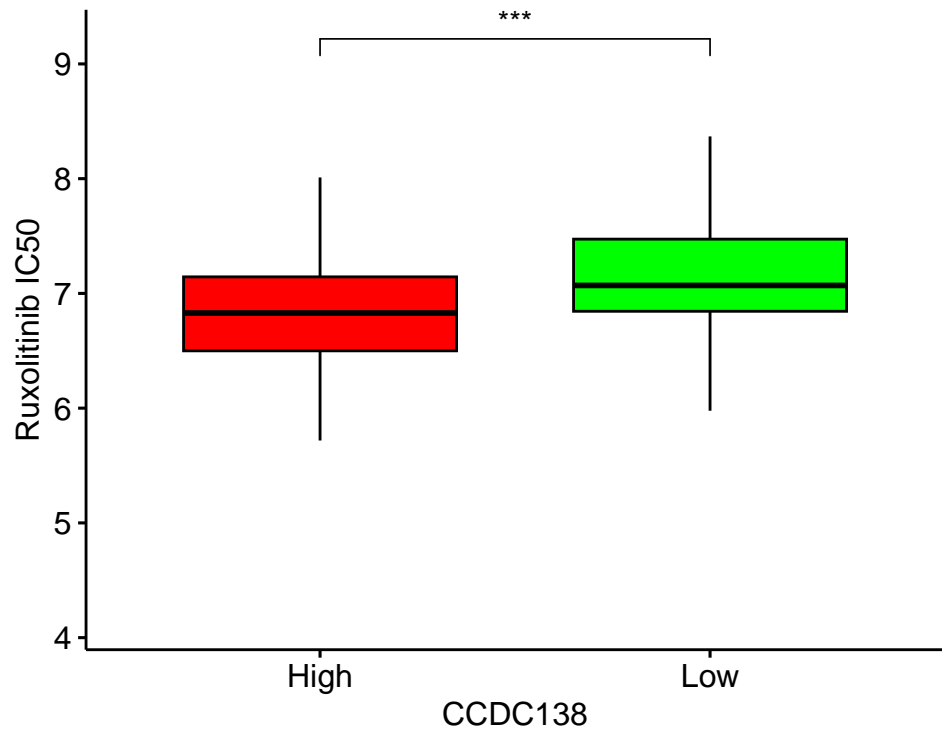

Supplement: Supplementary file 1 [file DataSheet1.zip › supplementary file/supplementary file 2/CCDC138_drugSenstivity.Ruxolitinib.pdf]

CCDC138 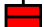 High 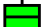 Low

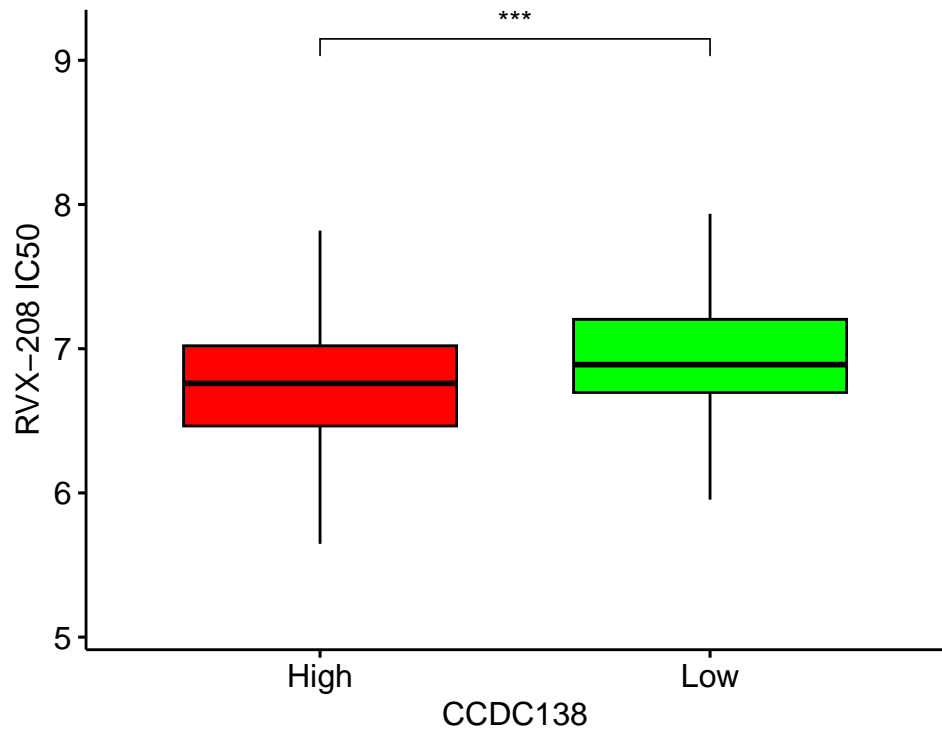

Supplement: Supplementary file 1 [file DataSheet1.zip › supplementary file/supplementary file 2/CCDC138_drugSenstivity.RVX-208.pdf]

CCDC138 High Low

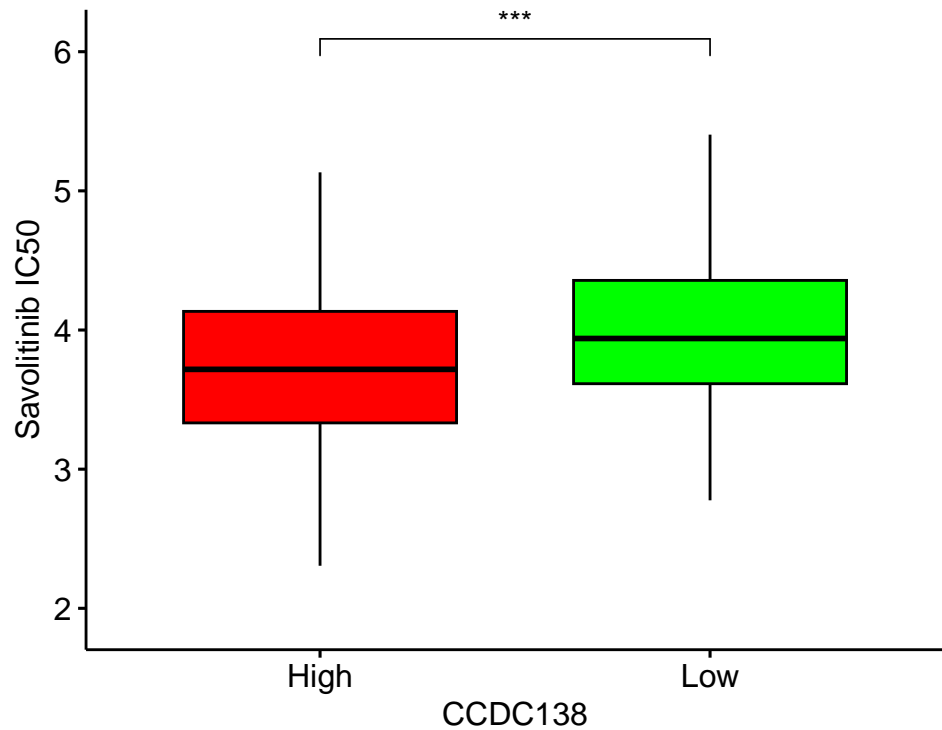

Supplement: Supplementary file 1 [file DataSheet1.zip › supplementary file/supplementary file 2/CCDC138_drugSenstivity.Savolitinib.pdf]

CCDC138 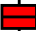 High 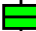 Low

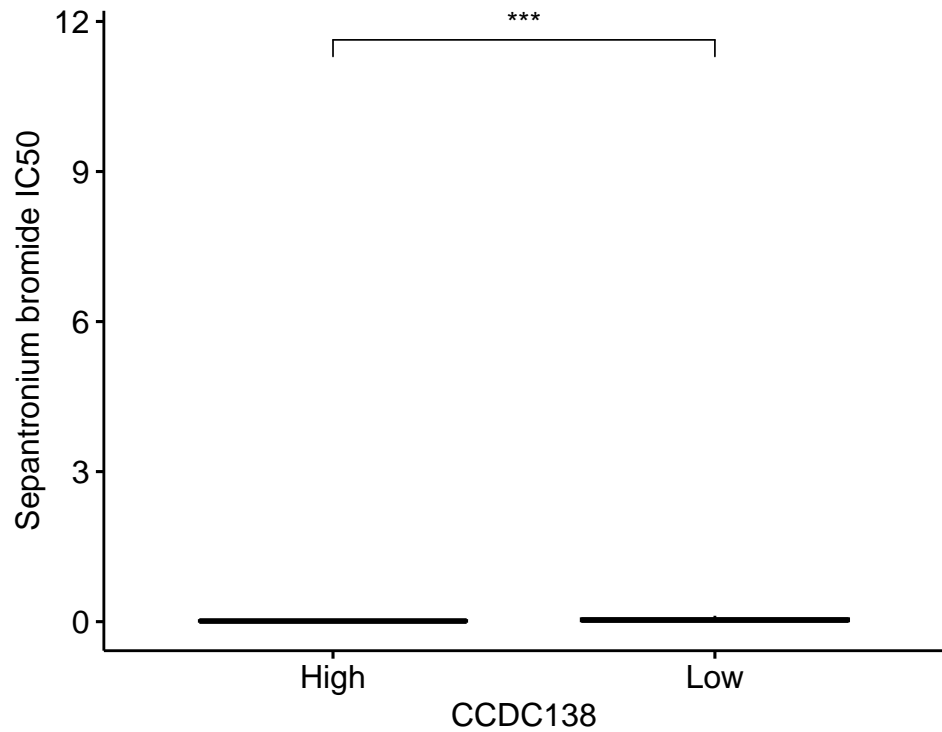

Supplement: Supplementary file 1 [file DataSheet1.zip › supplementary file/supplementary file 2/CCDC138_drugSenstivity.Sepantronium bromide.pdf]

CCDC138 High Low

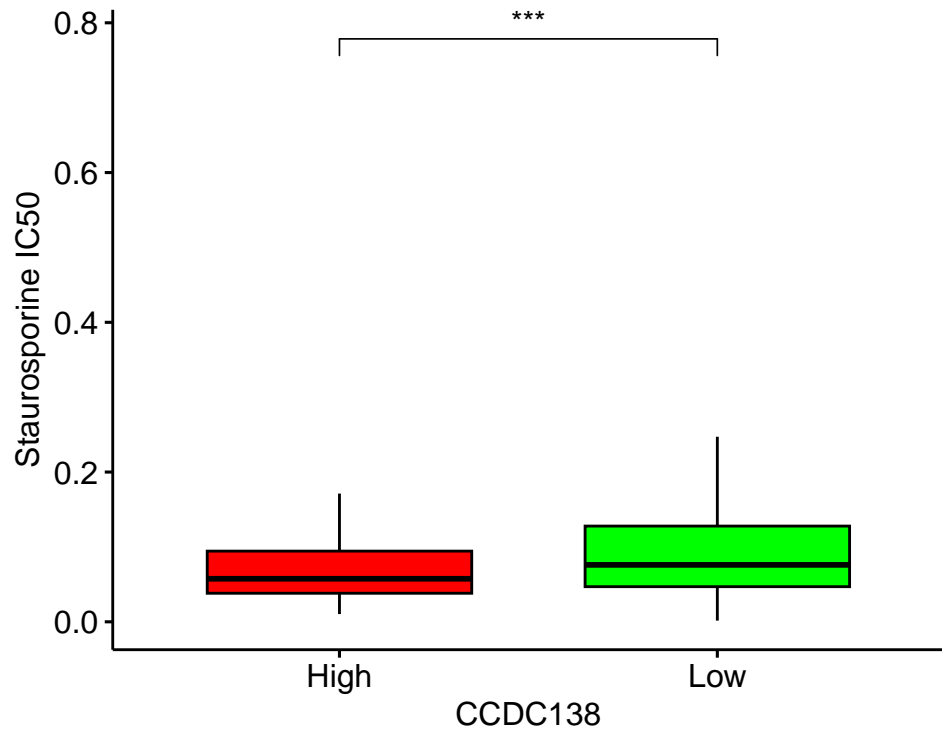

Supplement: Supplementary file 1 [file DataSheet1.zip › supplementary file/supplementary file 2/CCDC138_drugSenstivity.Staurosporine.pdf]

CCDC138 High Low

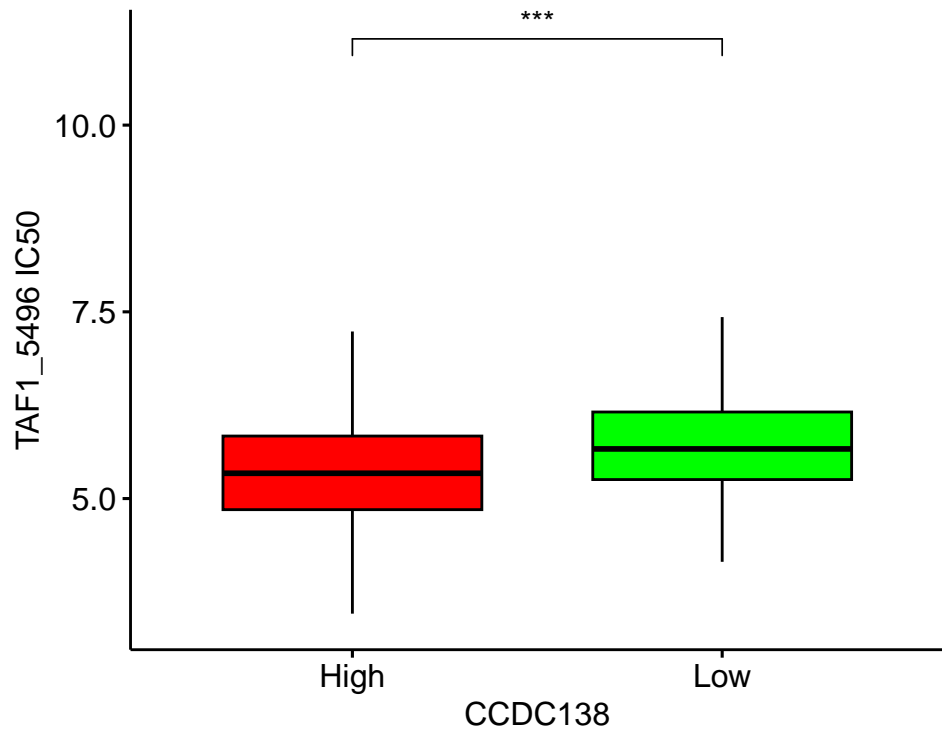

Supplement: Supplementary file 1 [file DataSheet1.zip › supplementary file/supplementary file 2/CCDC138_drugSenstivity.TAF1_5496.pdf]

CCDC138 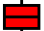 High 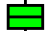 Low

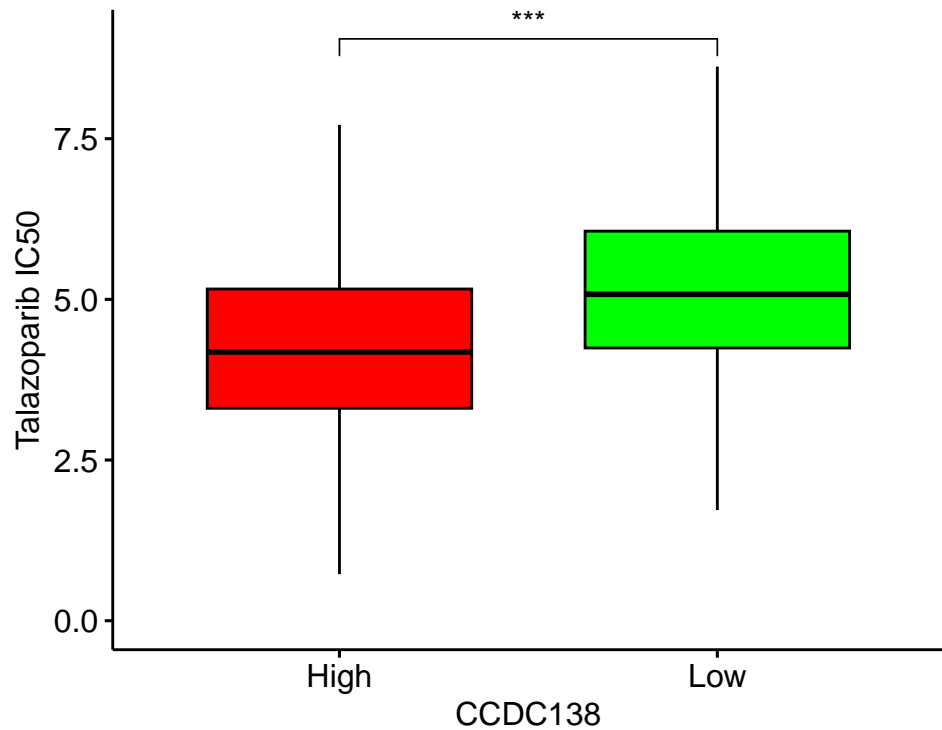

Supplement: Supplementary file 1 [file DataSheet1.zip › supplementary file/supplementary file 2/CCDC138_drugSenstivity.Talazoparib.pdf]

CCDC138 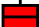 High 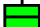 Low

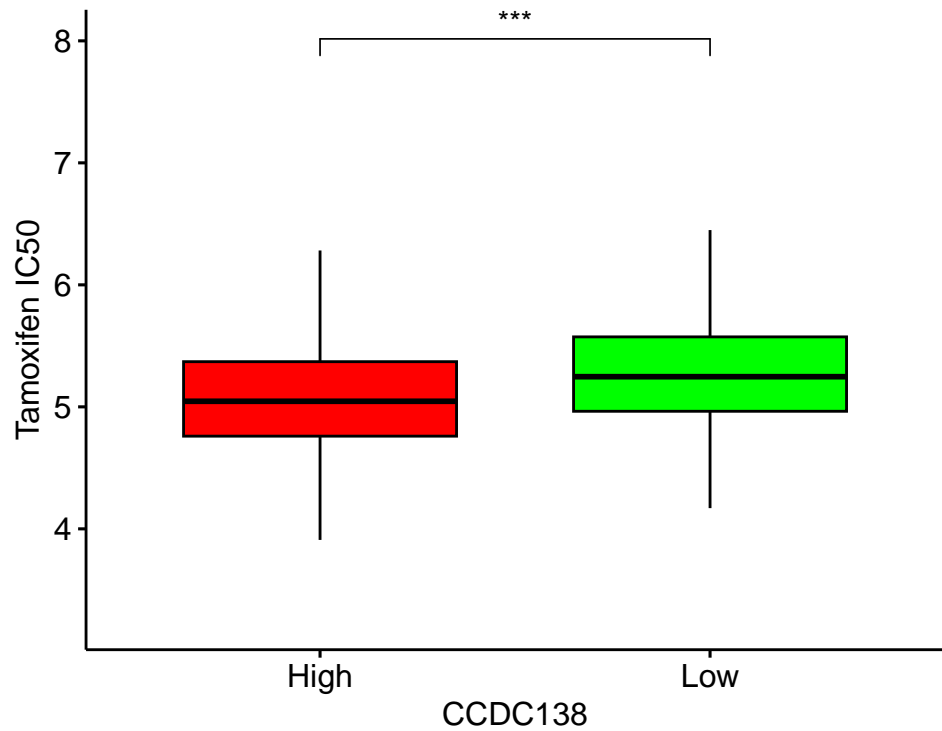

Supplement: Supplementary file 1 [file DataSheet1.zip › supplementary file/supplementary file 2/CCDC138_drugSenstivity.Tamoxifen.pdf]

CCDC138 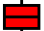 High 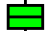 Low

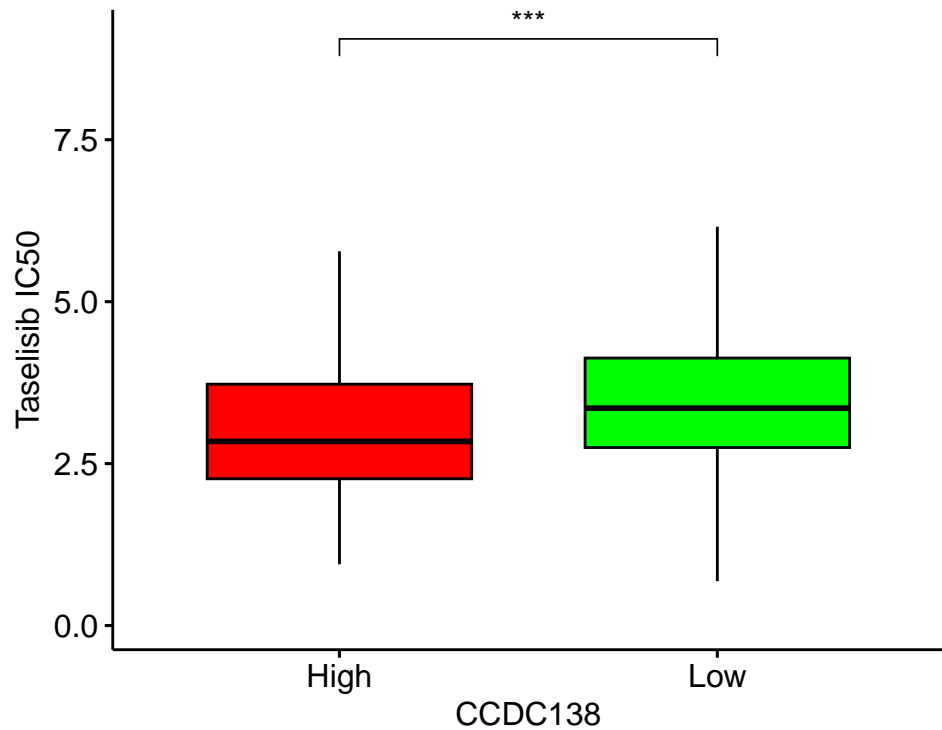

Supplement: Supplementary file 1 [file DataSheet1.zip › supplementary file/supplementary file 2/CCDC138_drugSenstivity.Taselisib.pdf]

CCDC138 High Low

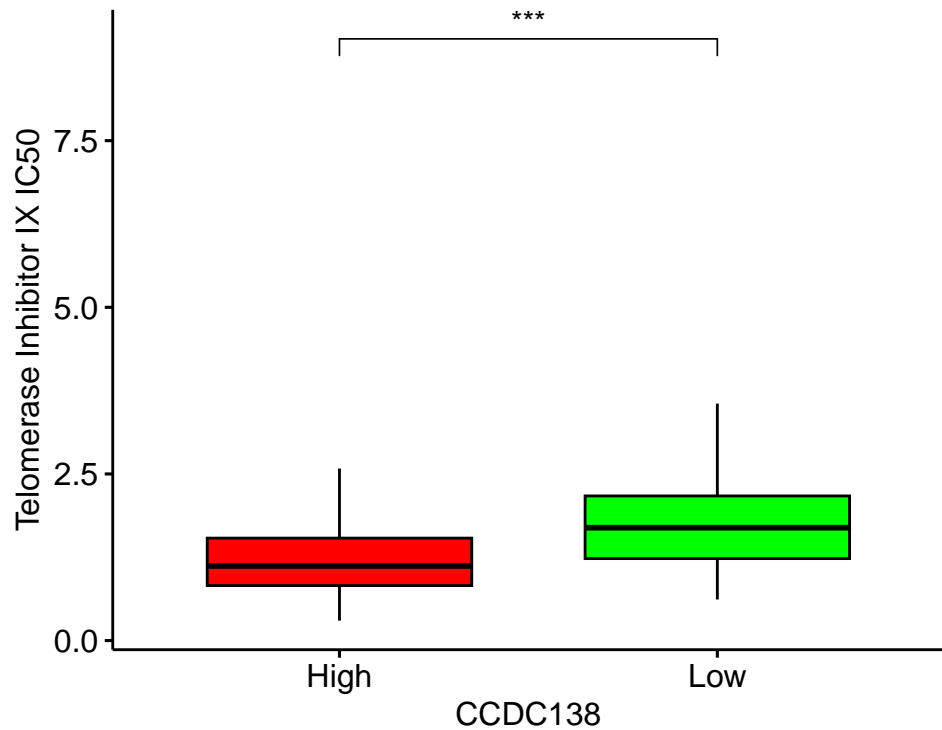

Supplement: Supplementary file 1 [file DataSheet1.zip › supplementary file/supplementary file 2/CCDC138_drugSenstivity.Telomerase Inhibitor IX.pdf]

CCDC138 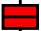 High 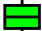 Low

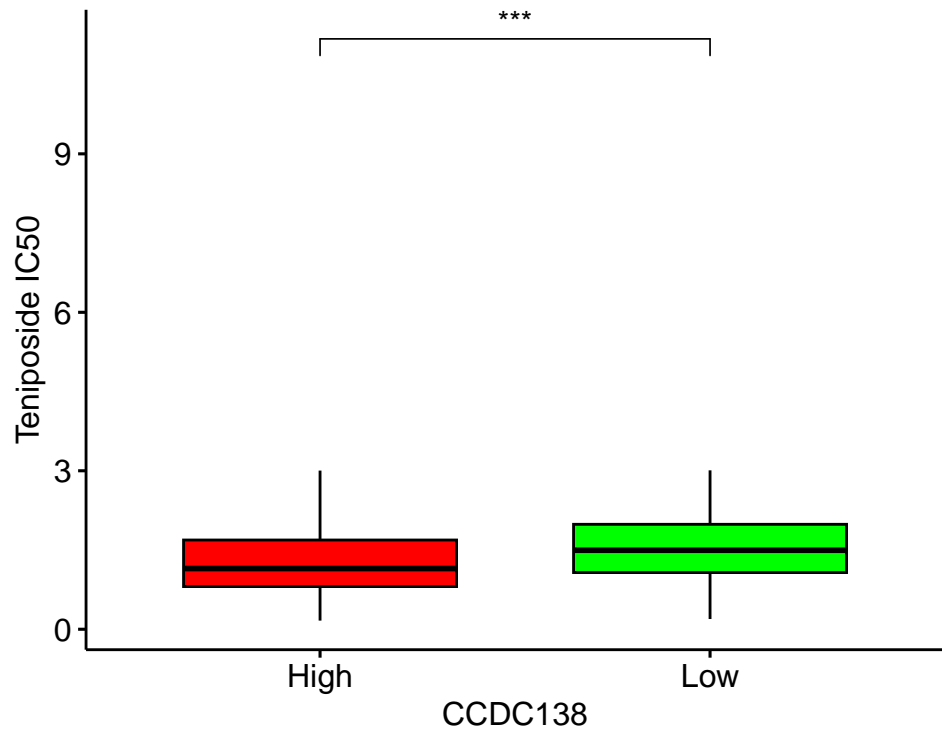

Supplement: Supplementary file 1 [file DataSheet1.zip › supplementary file/supplementary file 2/CCDC138_drugSenstivity.Teniposide.pdf]

CCDC138 High Low

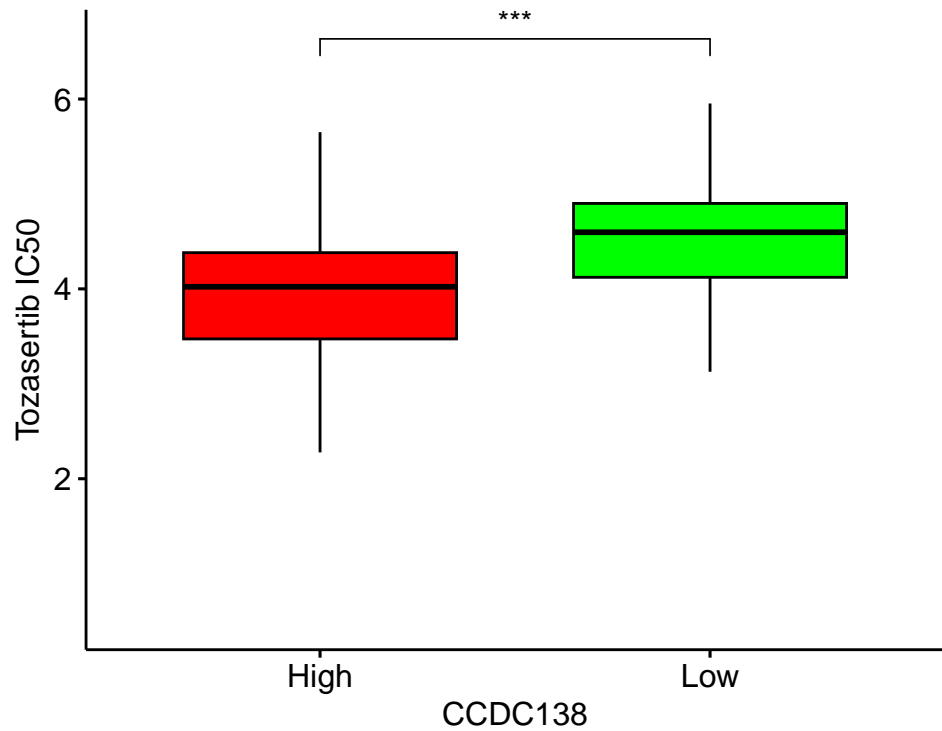

Supplement: Supplementary file 1 [file DataSheet1.zip › supplementary file/supplementary file 2/CCDC138_drugSenstivity.Tozasertib.pdf]

CCDC138 High Low

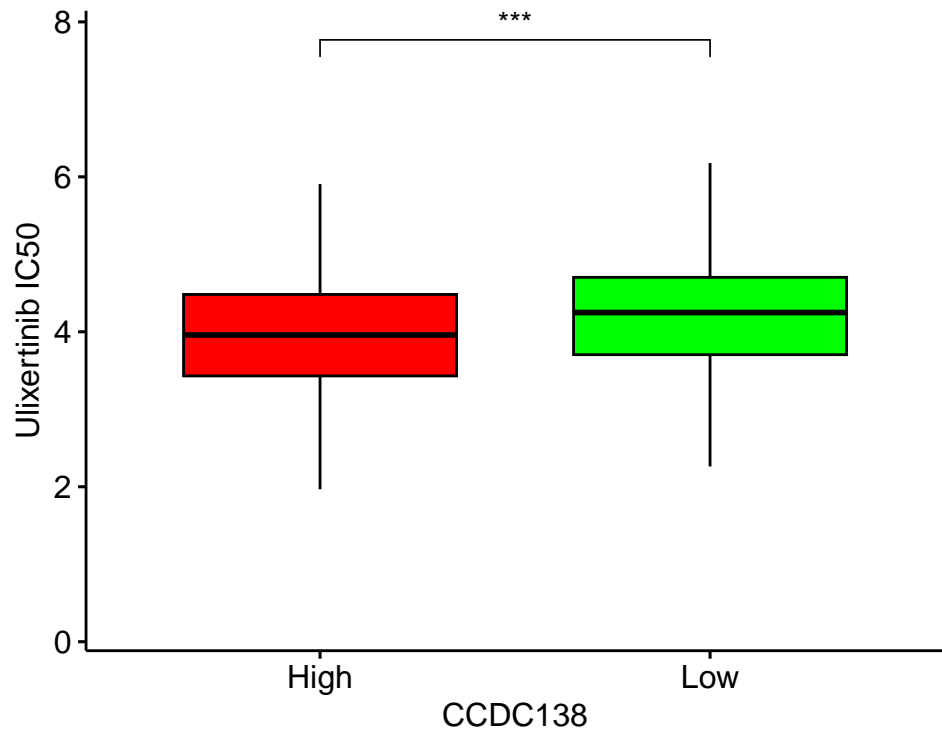

Supplement: Supplementary file 1 [file DataSheet1.zip › supplementary file/supplementary file 2/CCDC138_drugSenstivity.Ulixertinib.pdf]

CCDC138 High Low

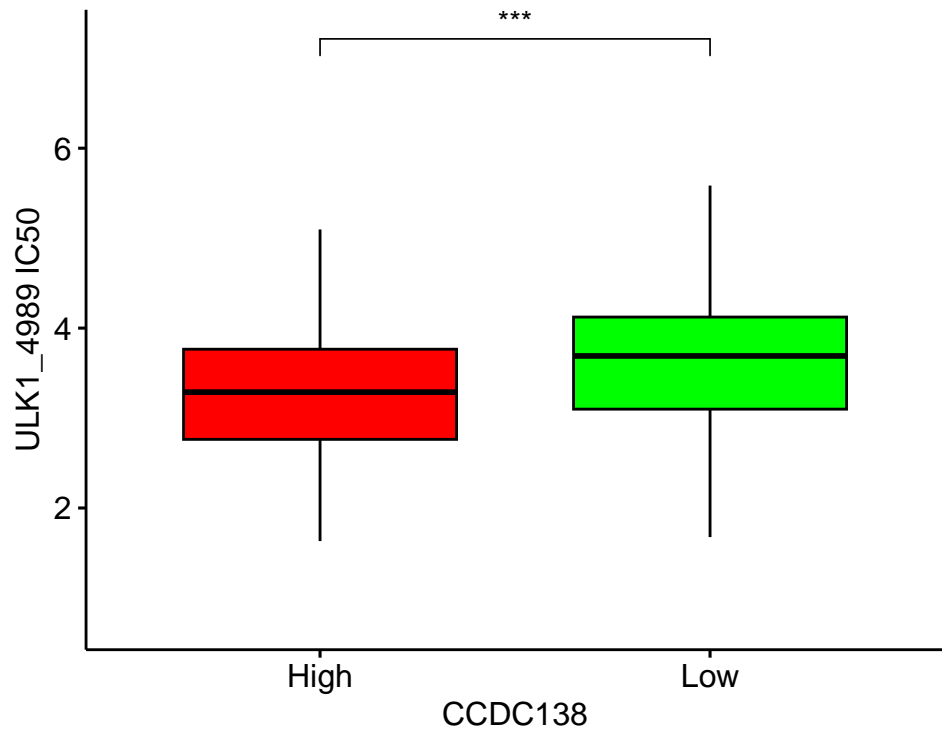

Supplement: Supplementary file 1 [file DataSheet1.zip › supplementary file/supplementary file 2/CCDC138_drugSenstivity.ULK1_4989.pdf]

CCDC138 High Low

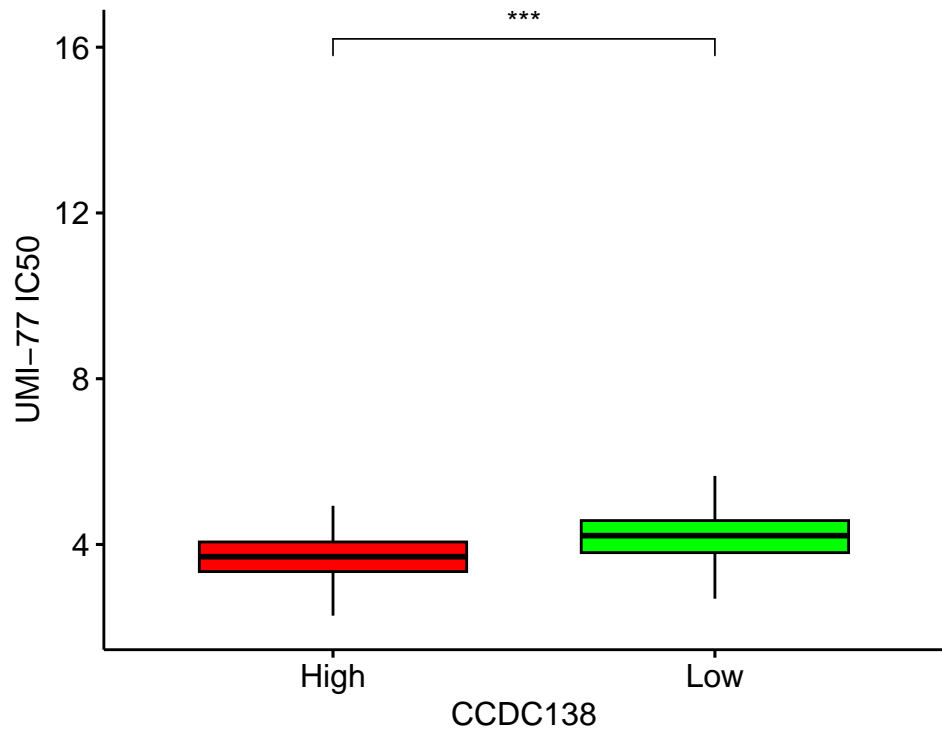

Supplement: Supplementary file 1 [file DataSheet1.zip › supplementary file/supplementary file 2/CCDC138_drugSenstivity.UMI-77.pdf]

CCDC138 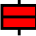 High 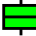 Low

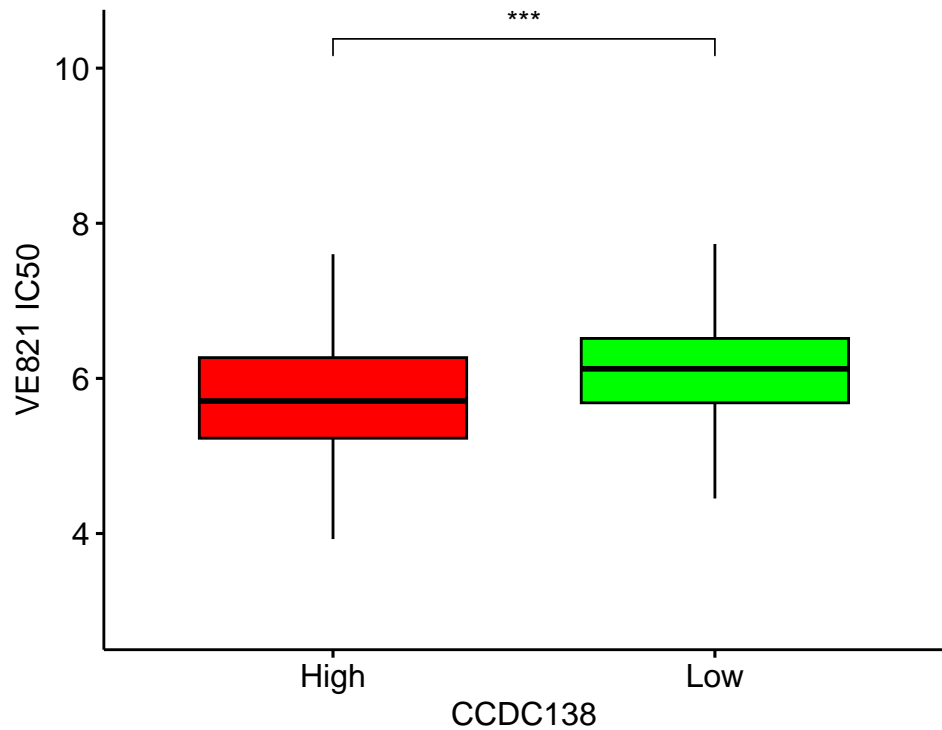

Supplement: Supplementary file 1 [file DataSheet1.zip › supplementary file/supplementary file 2/CCDC138_drugSenstivity.VE821.pdf]

CCDC138 High Low

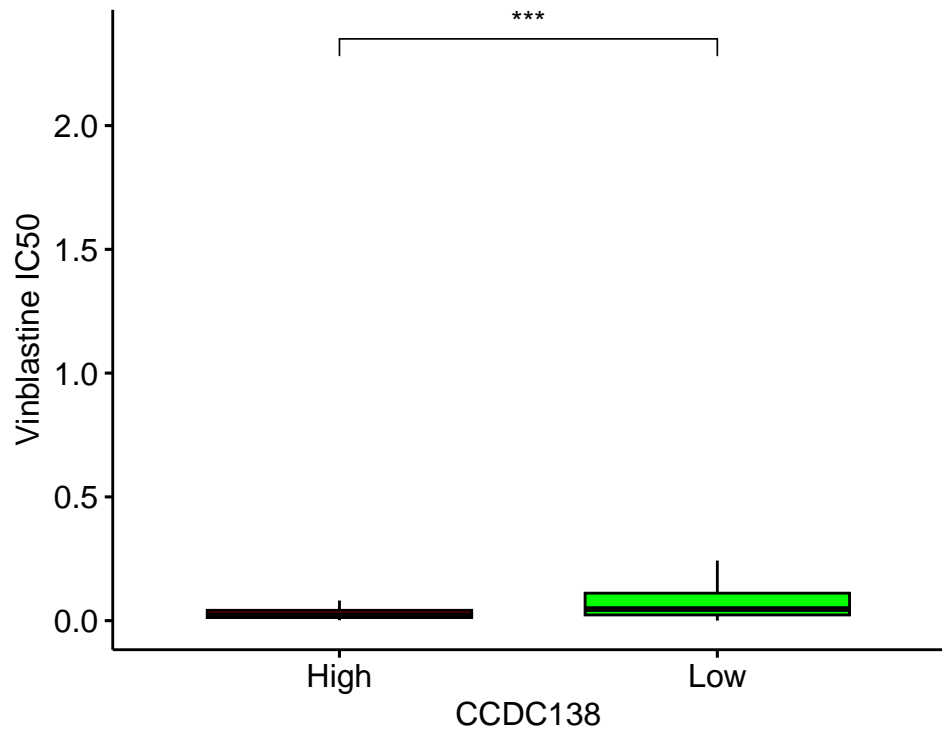

Supplement: Supplementary file 1 [file DataSheet1.zip › supplementary file/supplementary file 2/CCDC138_drugSenstivity.Vinblastine.pdf]

CCDC138 High Low

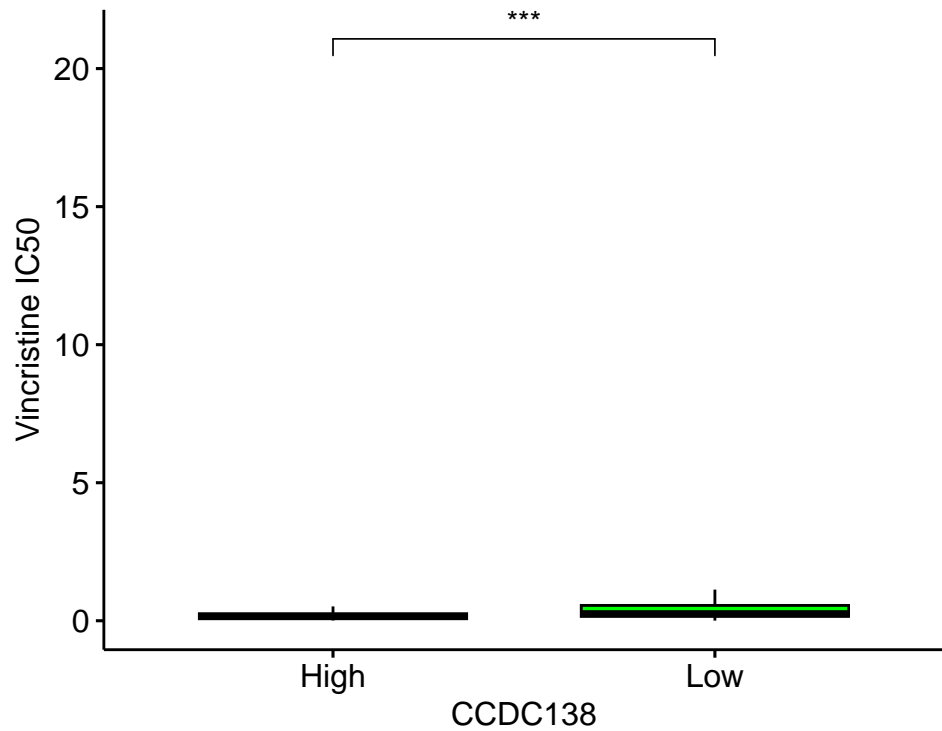

Supplement: Supplementary file 1 [file DataSheet1.zip › supplementary file/supplementary file 2/CCDC138_drugSenstivity.Vincristine.pdf]

CCDC138 High Low

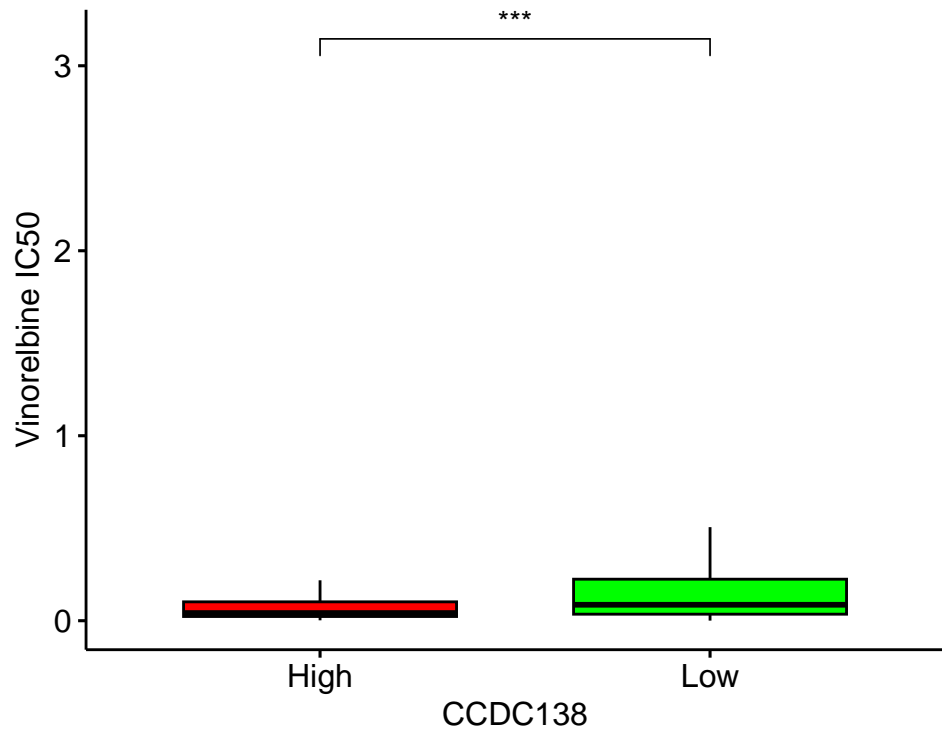

Supplement: Supplementary file 1 [file DataSheet1.zip › supplementary file/supplementary file 2/CCDC138_drugSenstivity.Vinorelbine.pdf]

CCDC138 High Low

\*\*\*

Wee1 Inhibitor IC50

10.0

7.5

5.0

2.5

High

Low

CCDC138

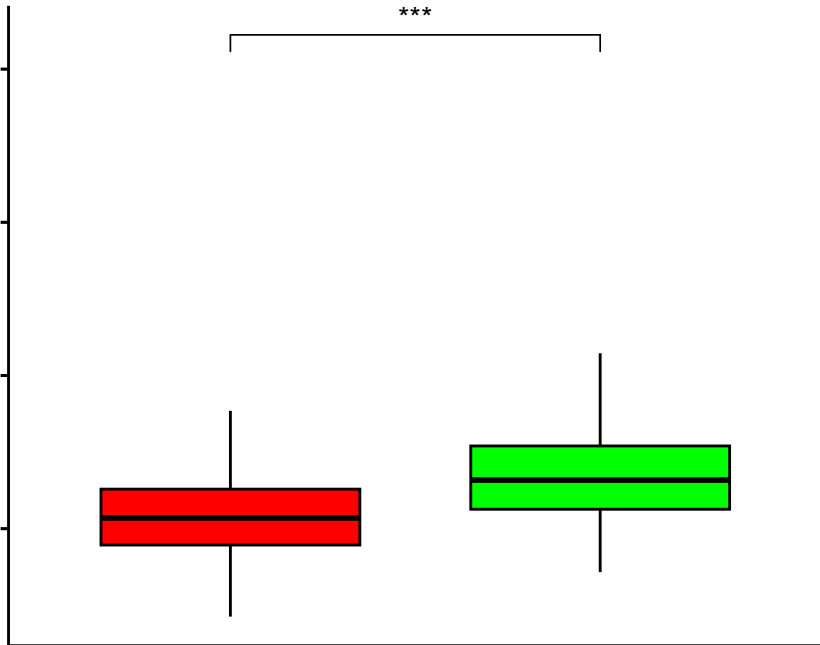

Supplement: Supplementary file 1 [file DataSheet1.zip › supplementary file/supplementary file 2/CCDC138_drugSenstivity.Wee1 Inhibitor.pdf]

CCDC138 High Low

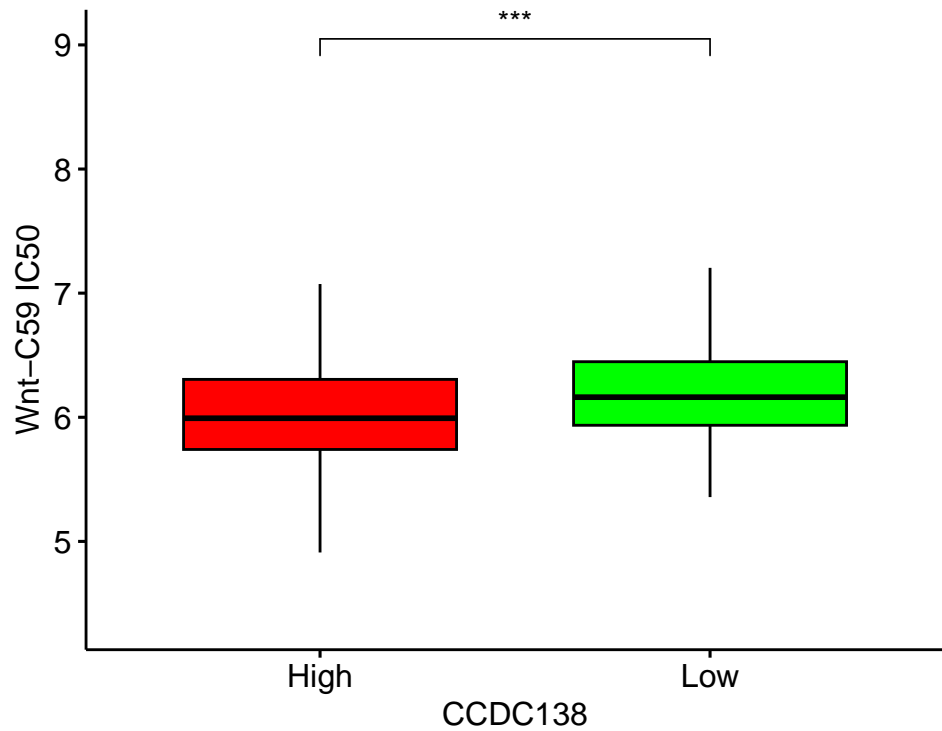

Supplement: Supplementary file 1 [file DataSheet1.zip › supplementary file/supplementary file 2/CCDC138_drugSenstivity.Wnt-C59.pdf]

CCDC138 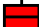 High 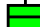 Low

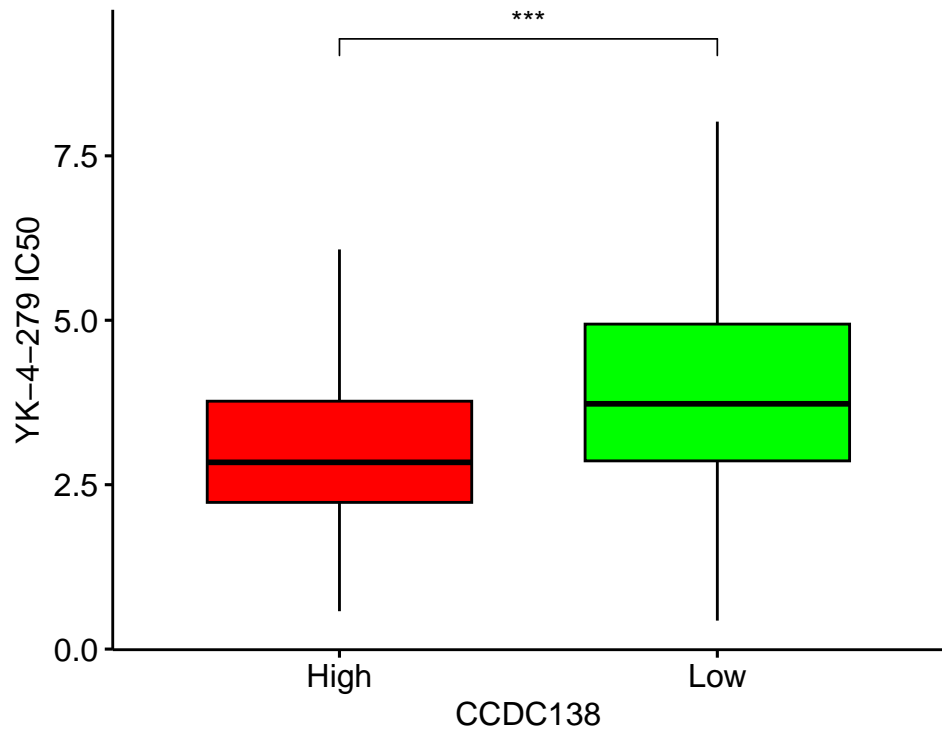

Supplement: Supplementary file 1 [file DataSheet1.zip › supplementary file/supplementary file 2/CCDC138_drugSenstivity.YK-4-279.pdf]

CCDC138 High Low

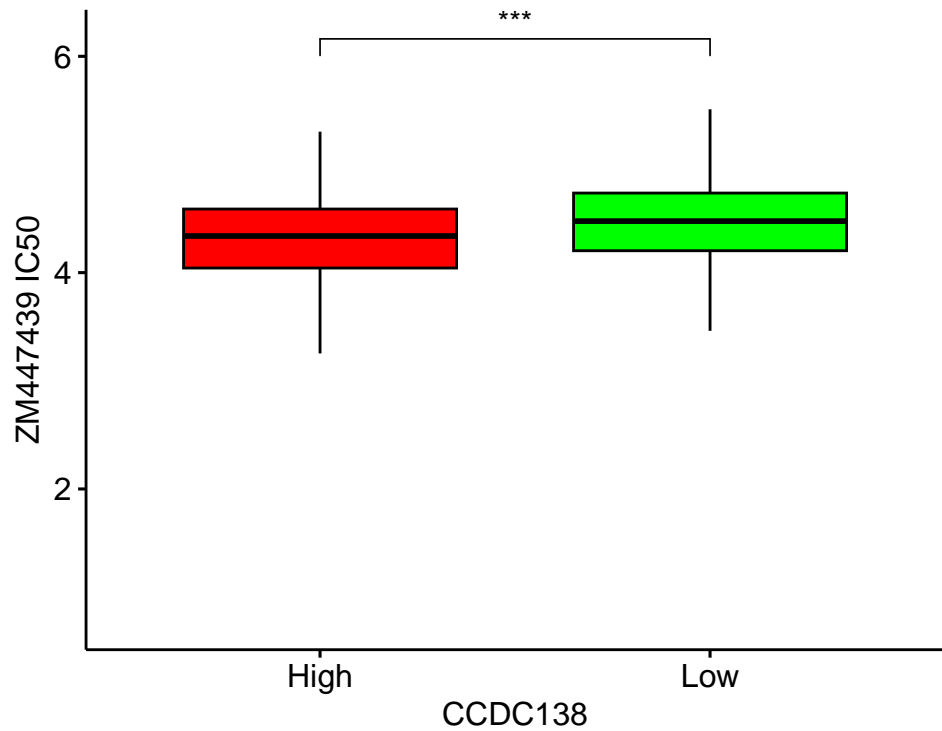

Supplement: Supplementary file 1 [file DataSheet1.zip › supplementary file/supplementary file 2/CCDC138_drugSenstivity.ZM447439.pdf]

CCDC138 High Low

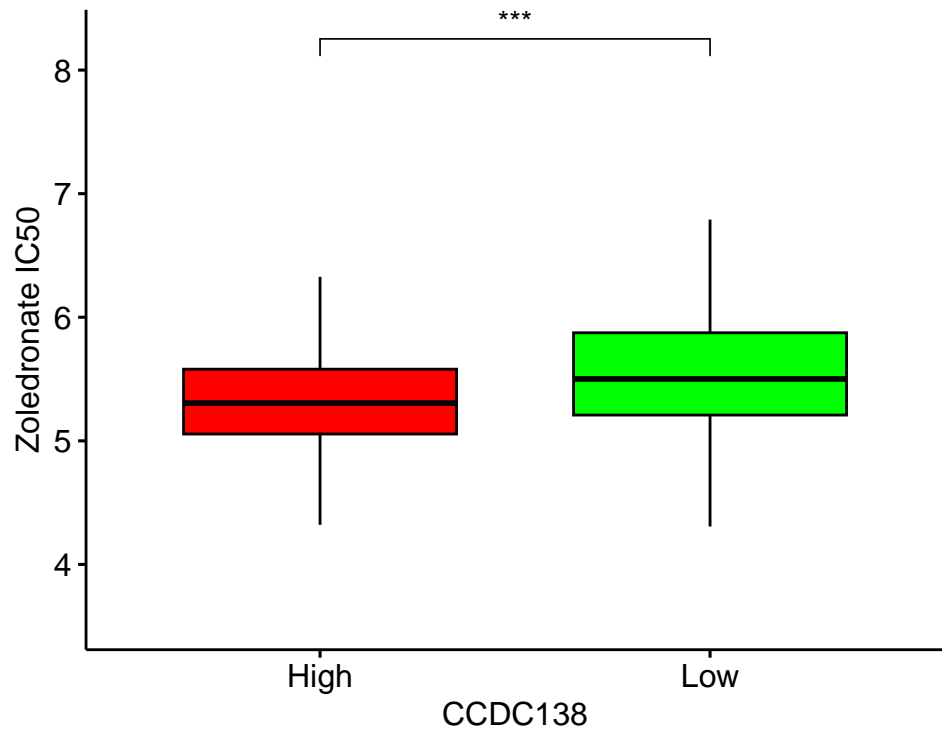

Supplement: Supplementary file 1 [file DataSheet1.zip › supplementary file/supplementary file 2/CCDC138_drugSenstivity.Zoledronate.pdf]
