# Supplementary figures and images for: CCDC138 overexpression predicts poor prognosis and highlights ciliopathy-linked mechanisms in uterine corpus endometrial carcinoma (part 1 of 2)
Source: Front Mol Biosci. 2025 Aug 8;12:1622496. doi: 10.3389/fmolb.2025.1622496 (PMC12370488; doi:10.3389/fmolb.2025.1622496)

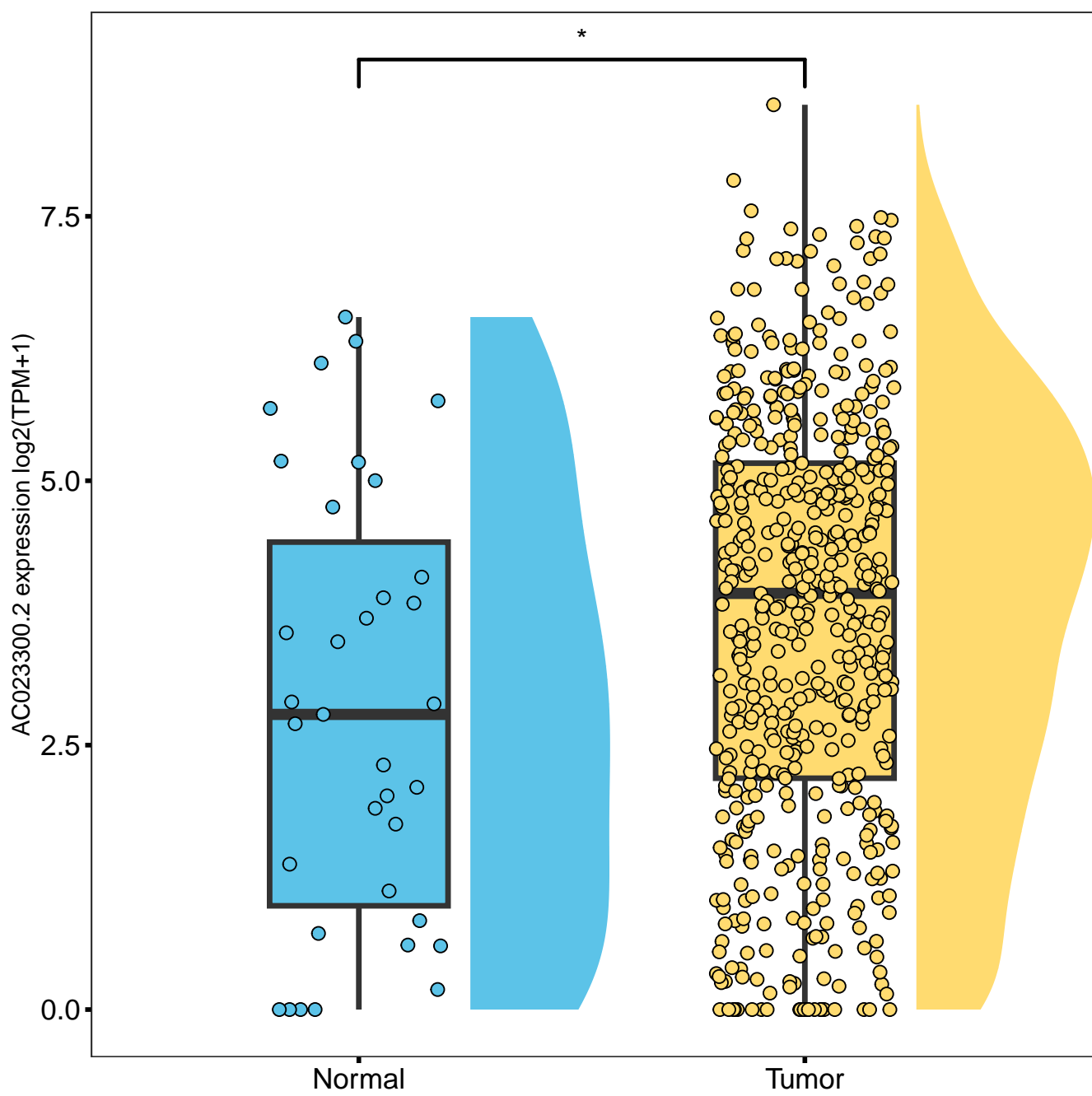

Supplement: Supplementary file 1 [file DataSheet1.zip › supplementary file/supplementary file 1/AC023300.2_boxplot.pdf]

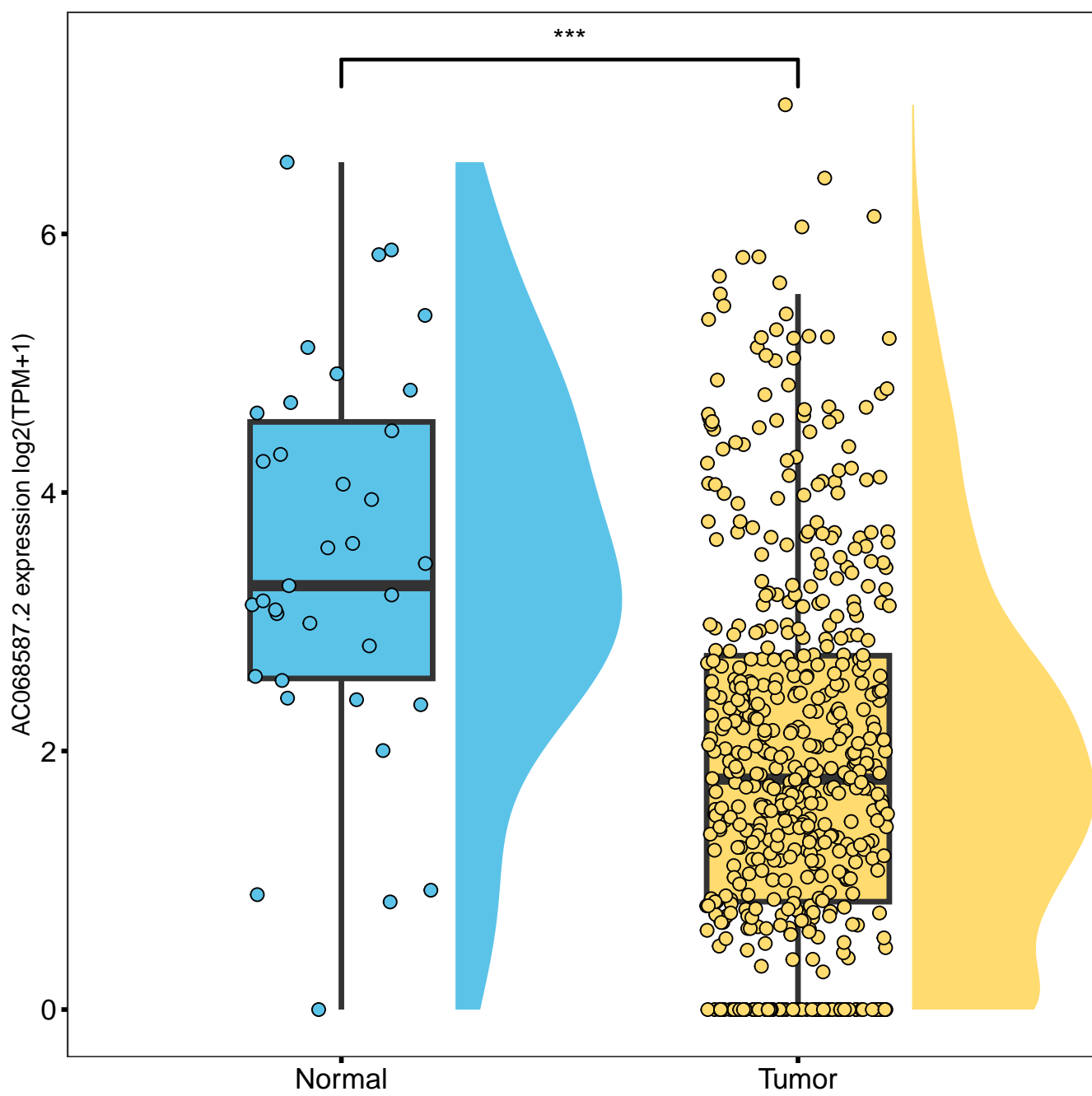

Supplement: Supplementary file 1 [file DataSheet1.zip › supplementary file/supplementary file 1/AC068587.2_boxplot.pdf]

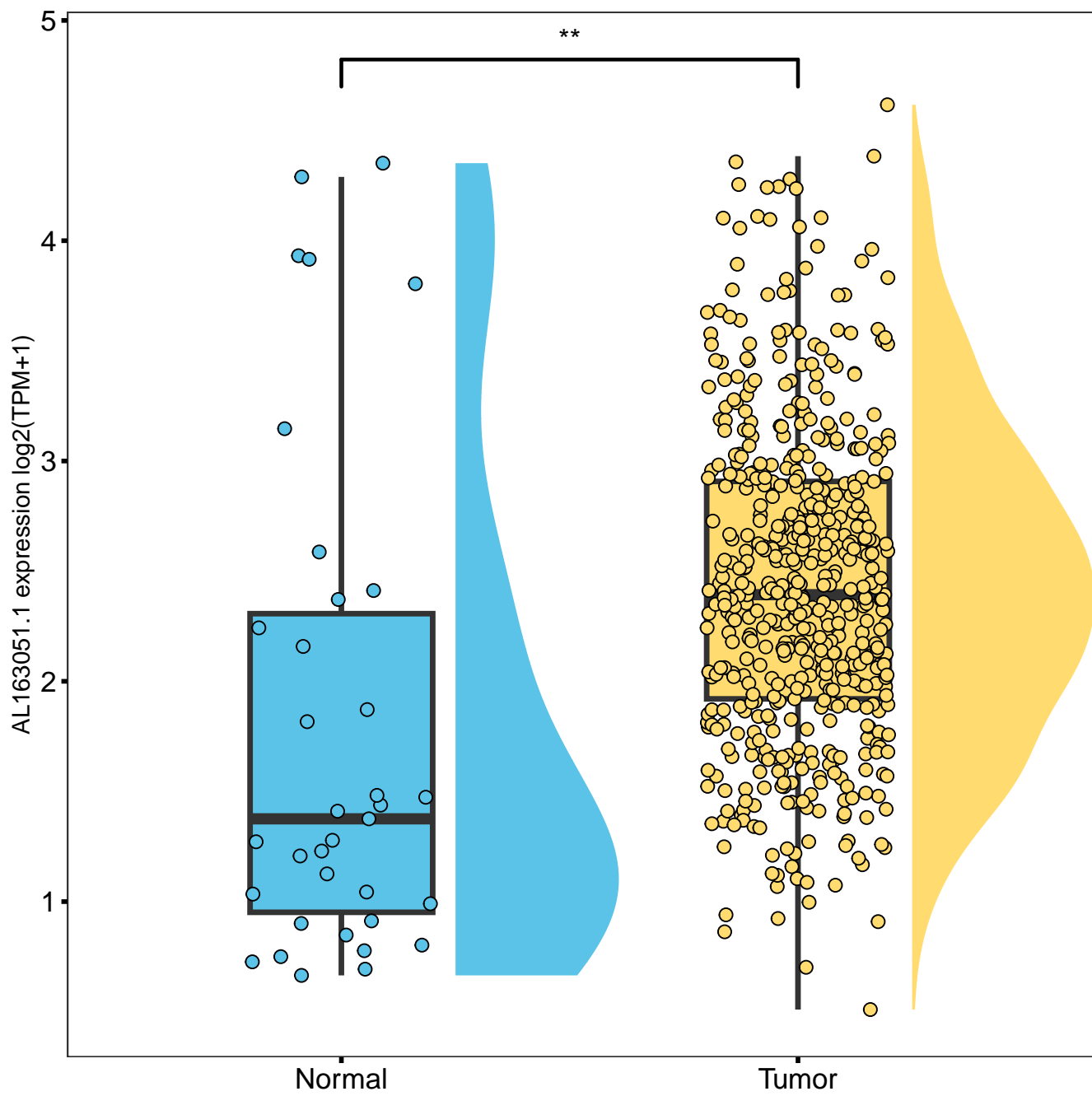

Supplement: Supplementary file 1 [file DataSheet1.zip › supplementary file/supplementary file 1/AL163051.1_boxplot.pdf]

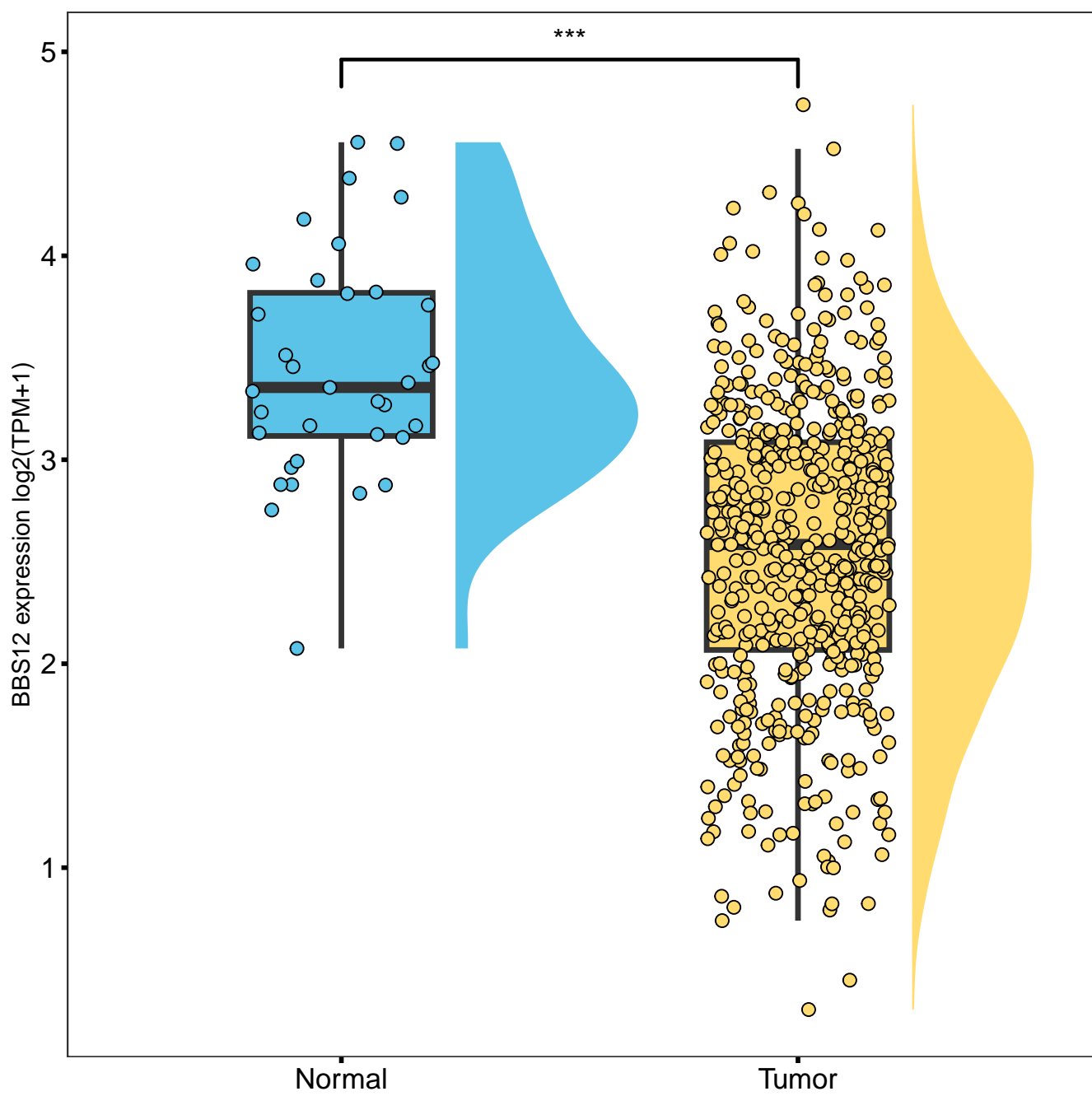

Supplement: Supplementary file 1 [file DataSheet1.zip › supplementary file/supplementary file 1/BBS12_boxplot.pdf]

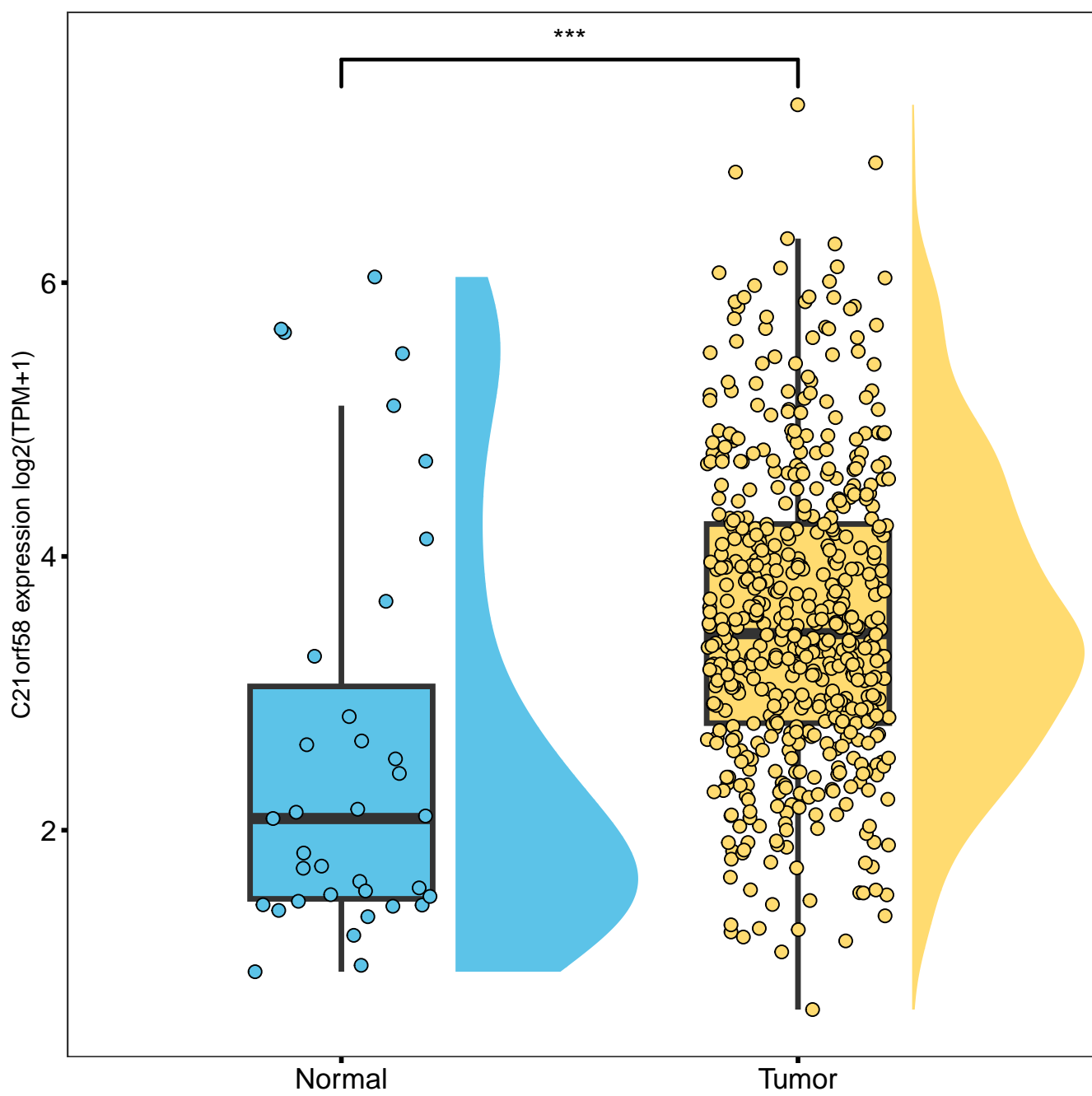

Supplement: Supplementary file 1 [file DataSheet1.zip › supplementary file/supplementary file 1/C21orf58_boxplot.pdf]

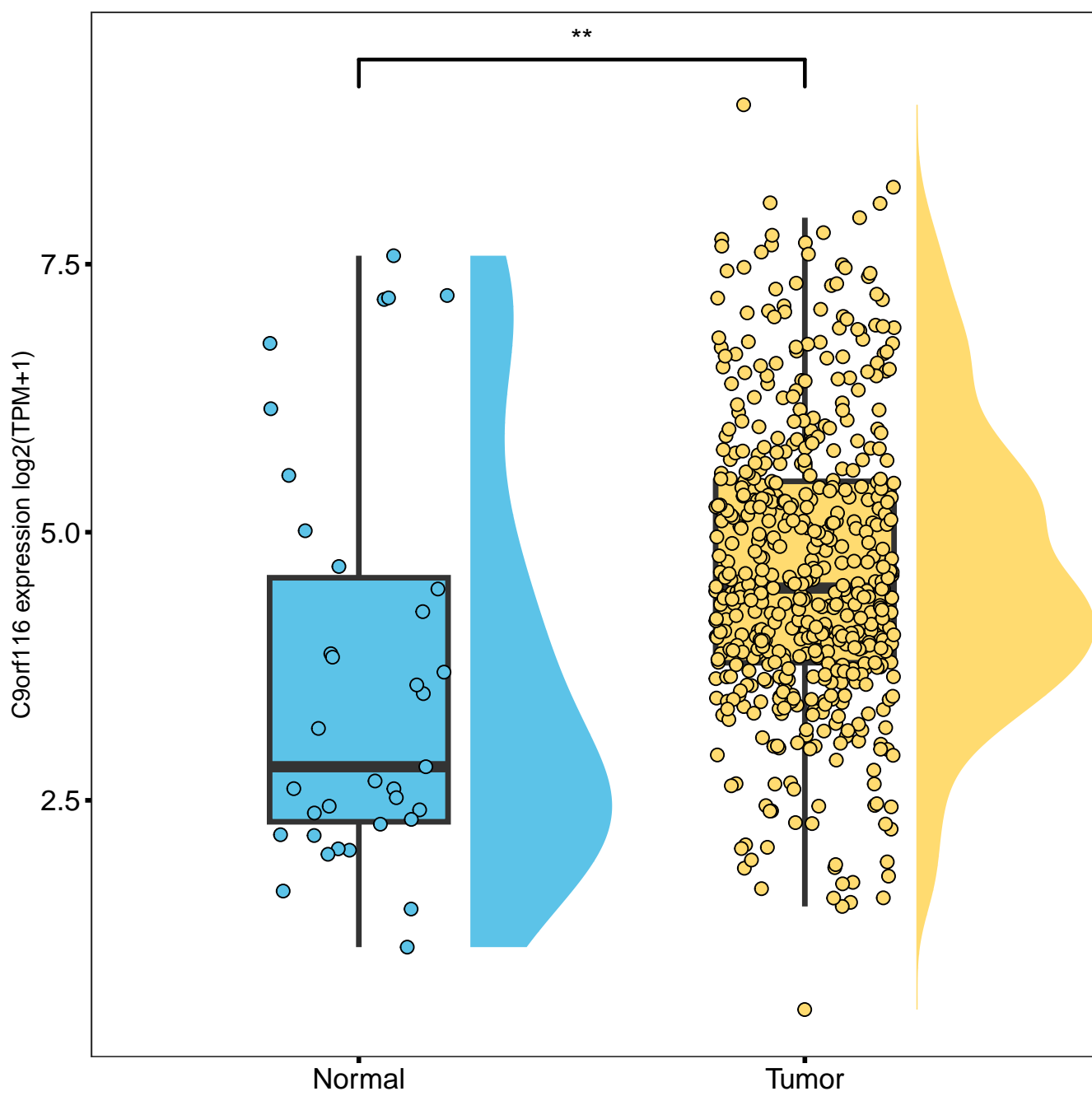

Supplement: Supplementary file 1 [file DataSheet1.zip › supplementary file/supplementary file 1/C9orf116_boxplot.pdf]

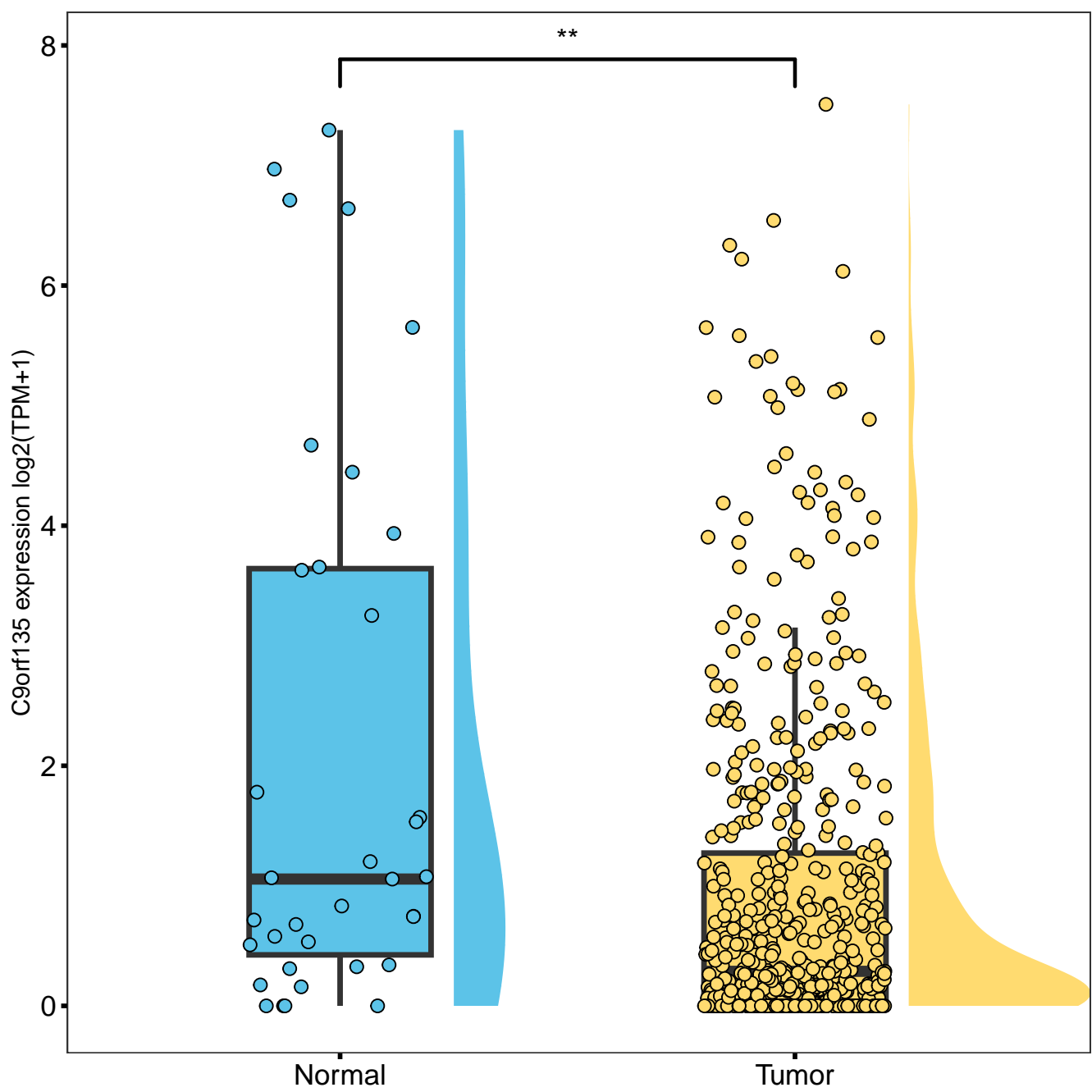

Supplement: Supplementary file 1 [file DataSheet1.zip › supplementary file/supplementary file 1/C9orf135_boxplot.pdf]

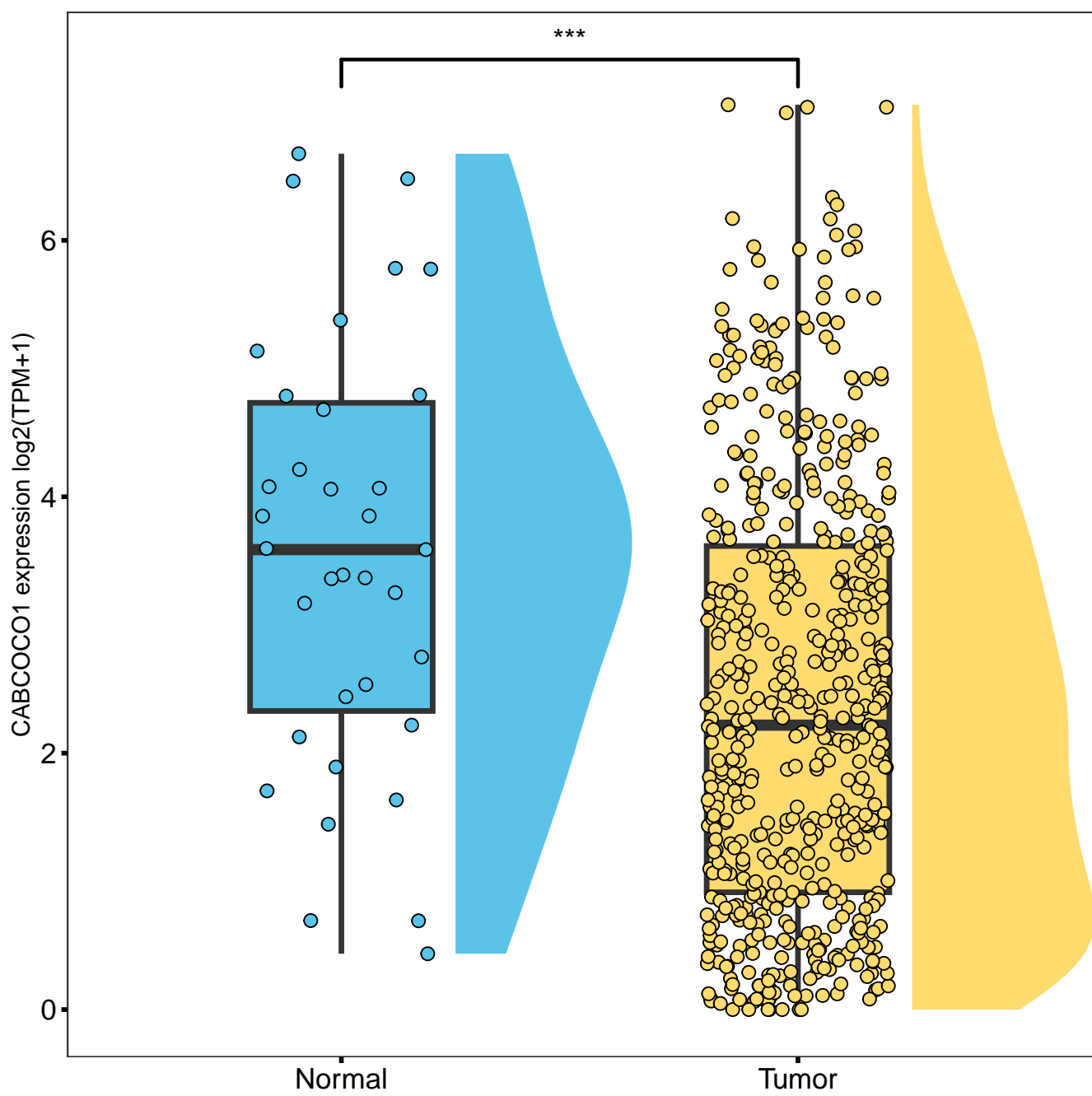

Supplement: Supplementary file 1 [file DataSheet1.zip › supplementary file/supplementary file 1/CABCOCO1_boxplot.pdf]

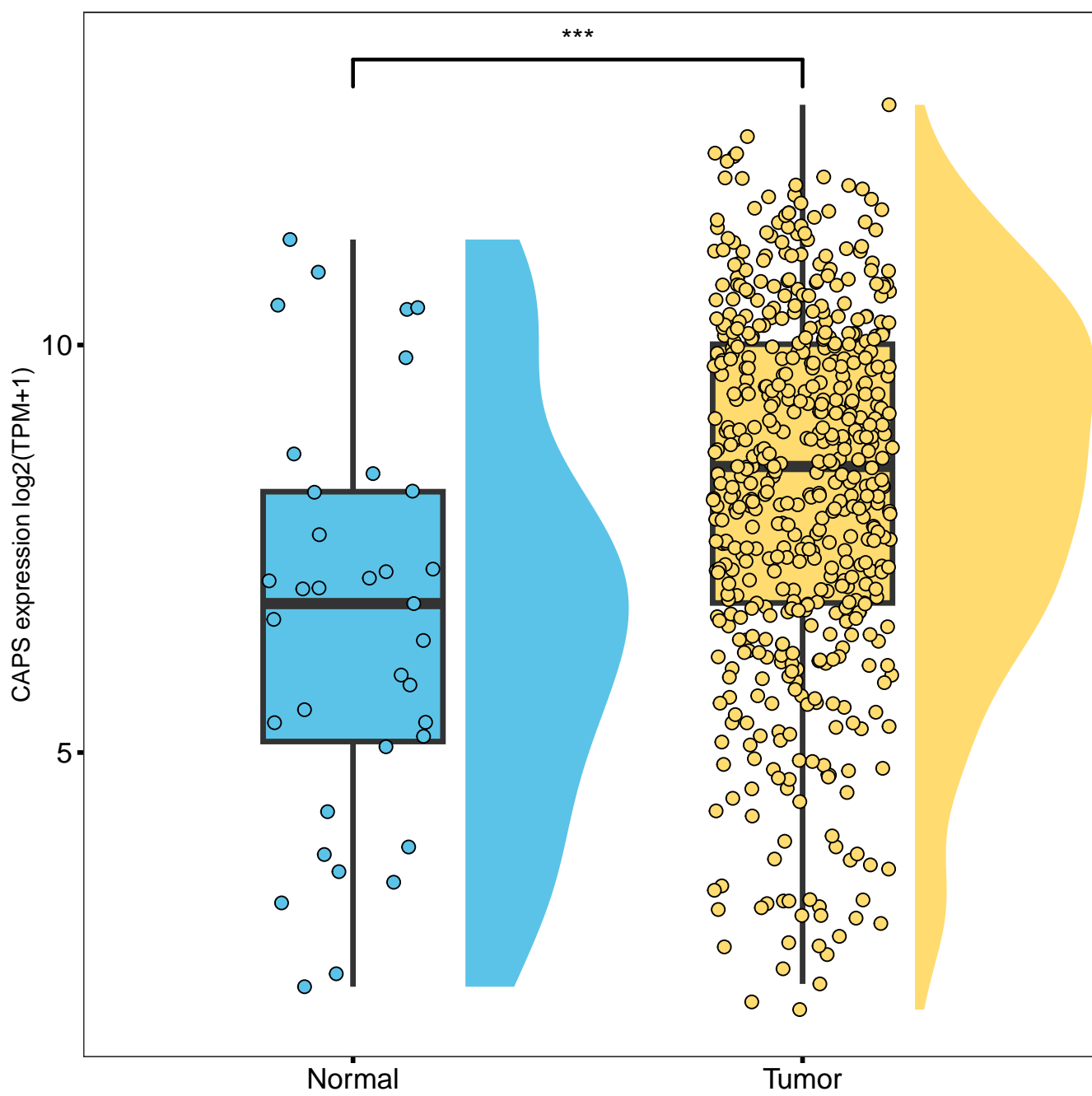

Supplement: Supplementary file 1 [file DataSheet1.zip › supplementary file/supplementary file 1/CAPS_boxplot.pdf]

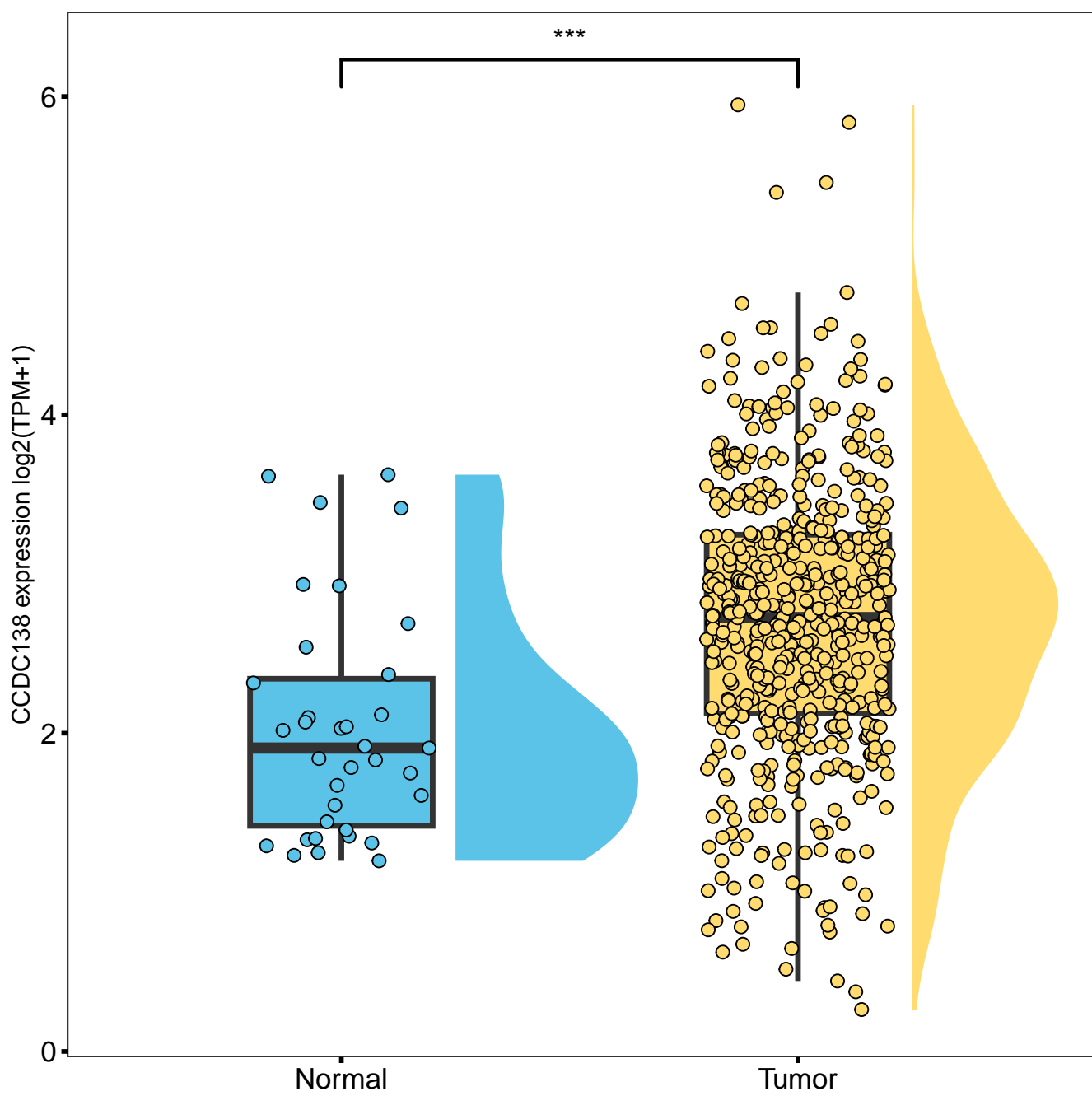

Supplement: Supplementary file 1 [file DataSheet1.zip › supplementary file/supplementary file 1/CCDC138_boxplot.pdf]

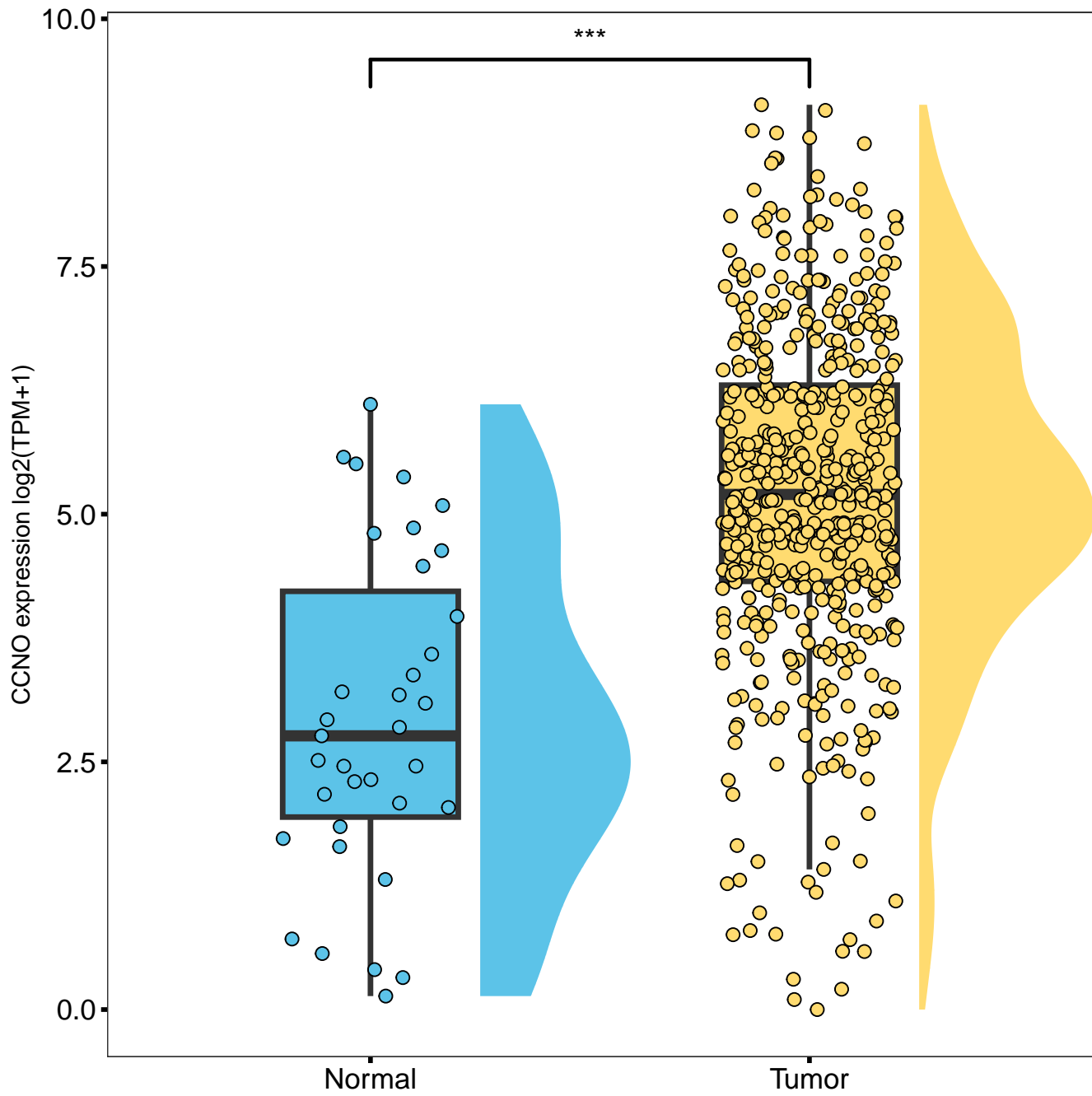

Supplement: Supplementary file 1 [file DataSheet1.zip › supplementary file/supplementary file 1/CCNO_boxplot.pdf]

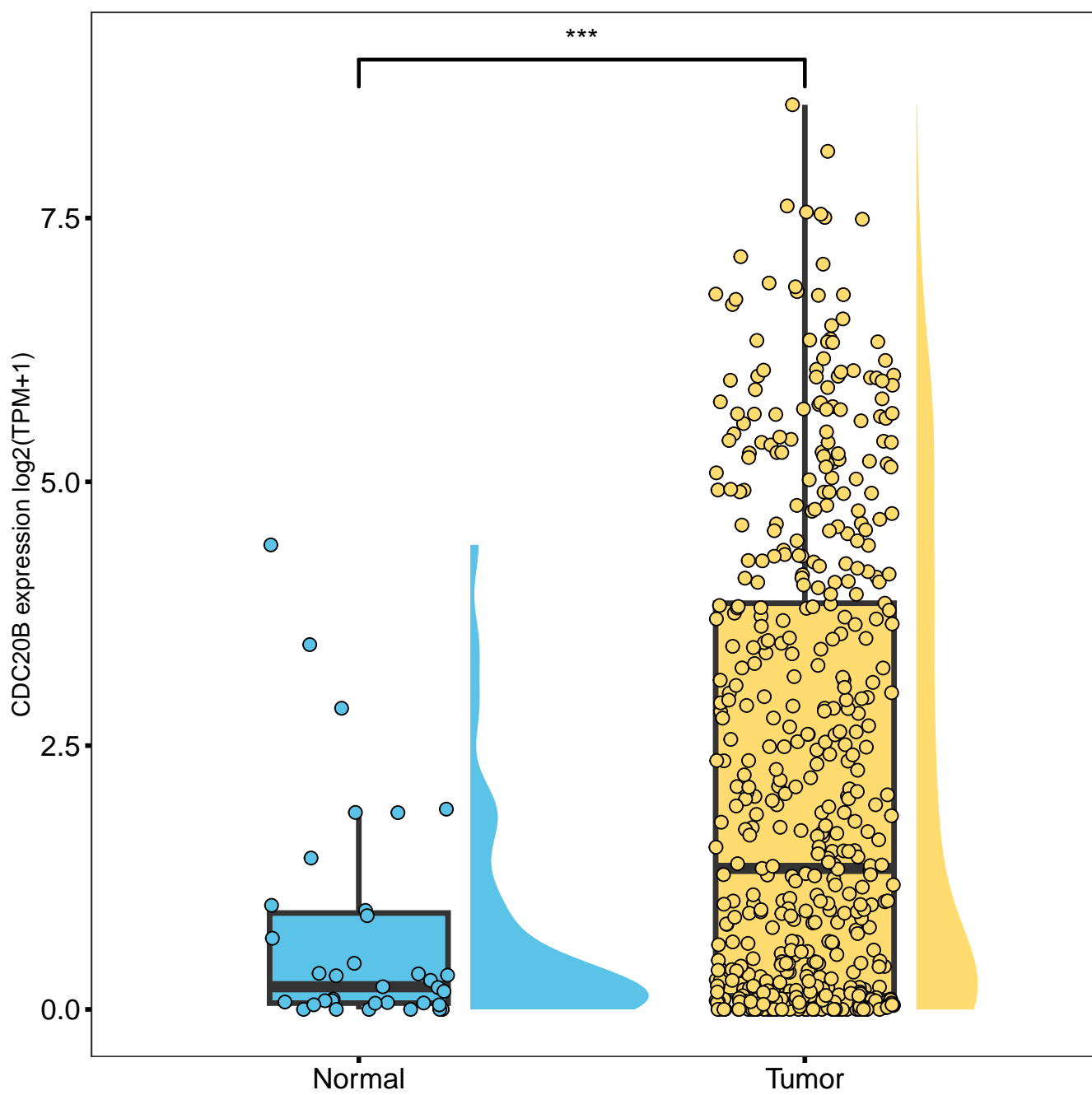

Supplement: Supplementary file 1 [file DataSheet1.zip › supplementary file/supplementary file 1/CDC20B_boxplot.pdf]

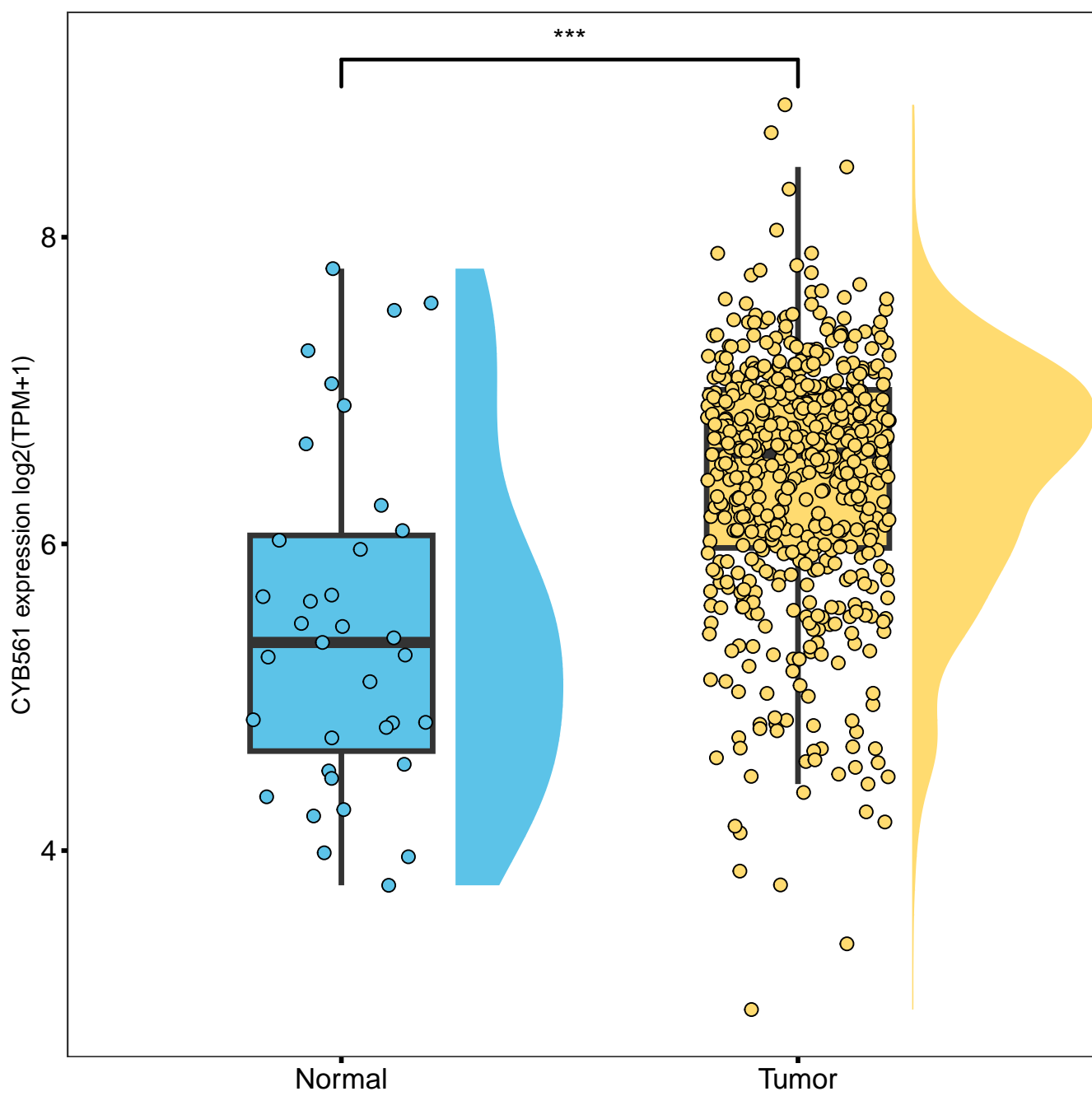

Supplement: Supplementary file 1 [file DataSheet1.zip › supplementary file/supplementary file 1/CYB561_boxplot.pdf]

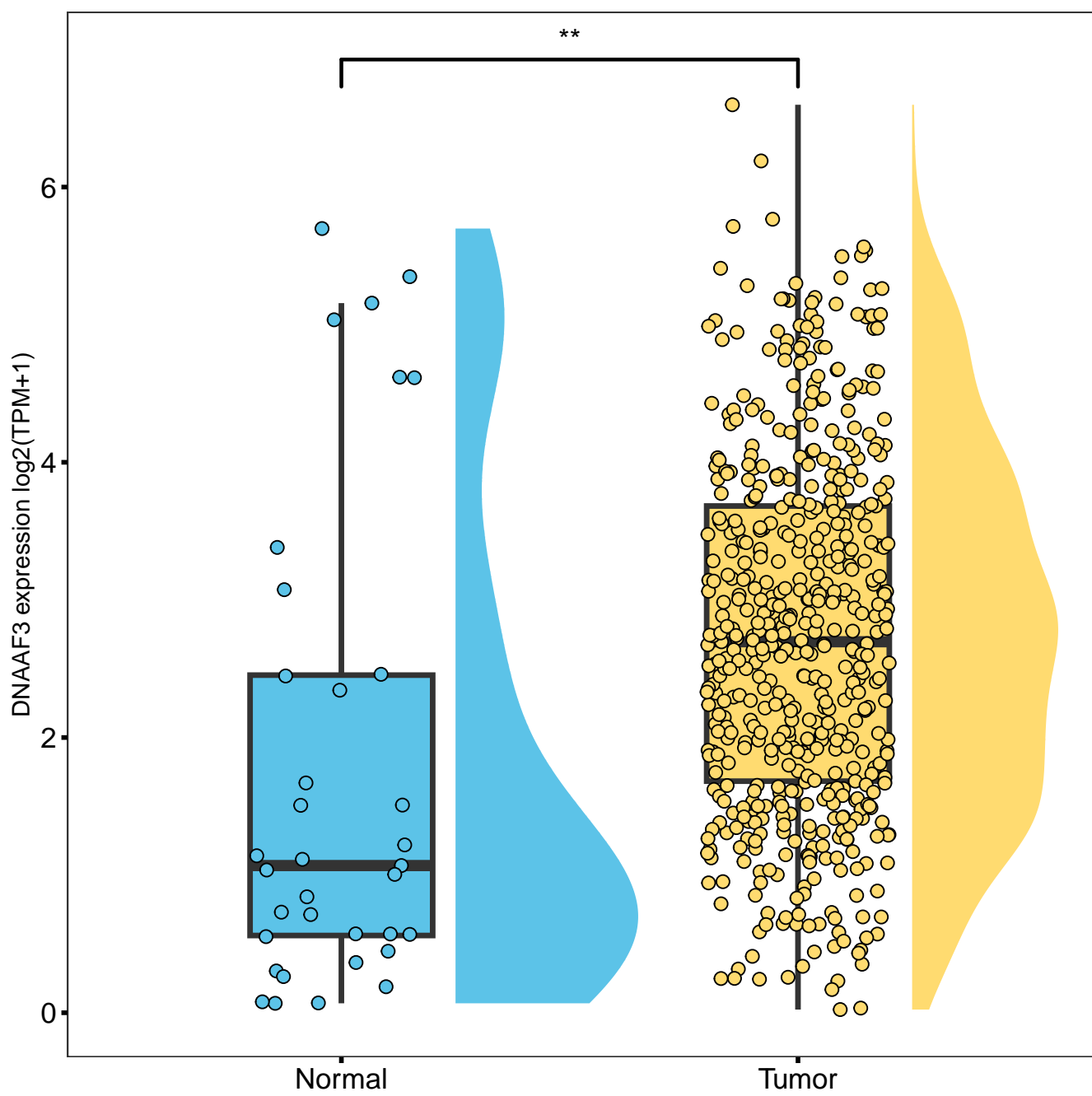

Supplement: Supplementary file 1 [file DataSheet1.zip › supplementary file/supplementary file 1/DNAAF3_boxplot.pdf]

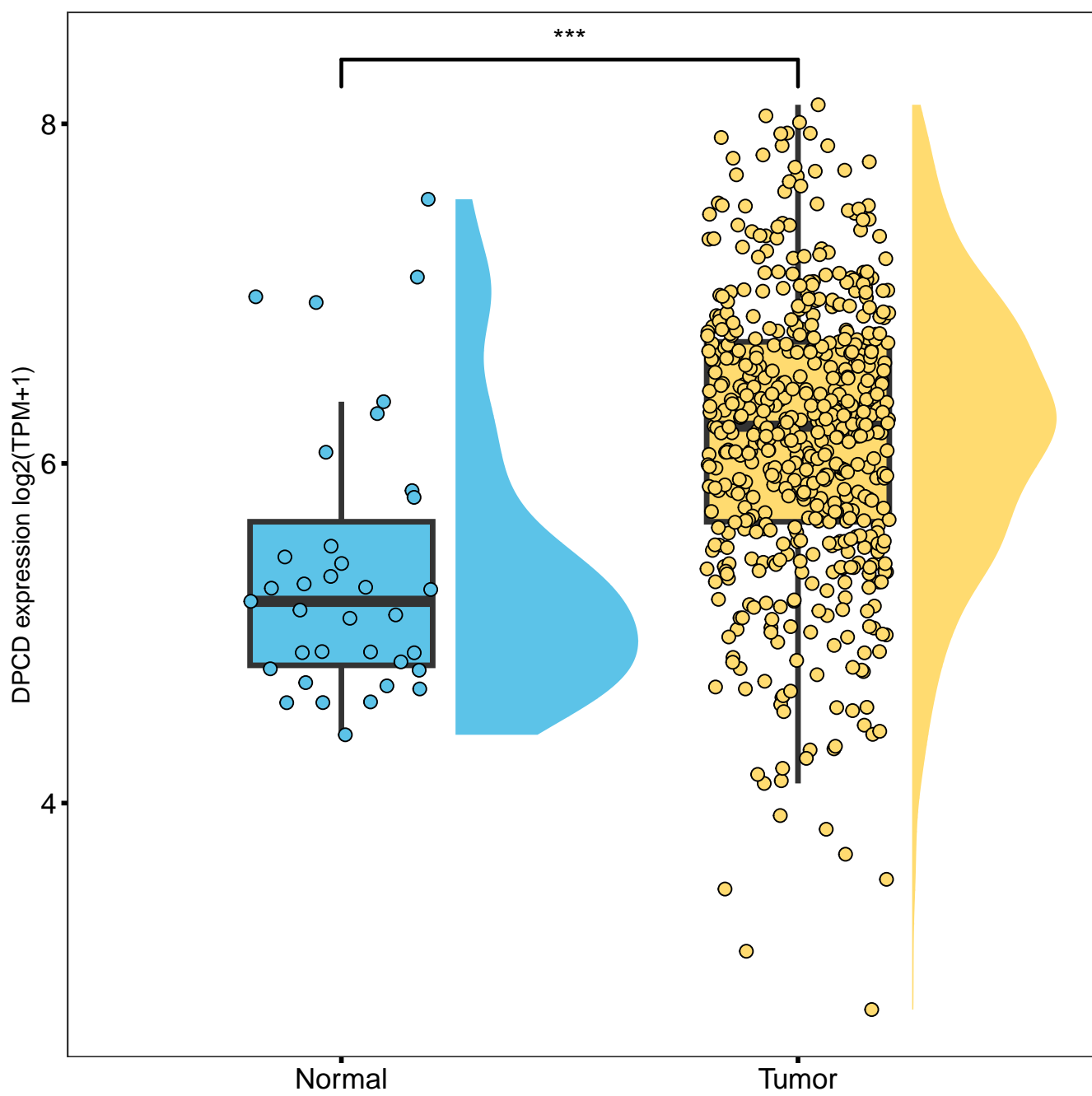

Supplement: Supplementary file 1 [file DataSheet1.zip › supplementary file/supplementary file 1/DPCD_boxplot.pdf]

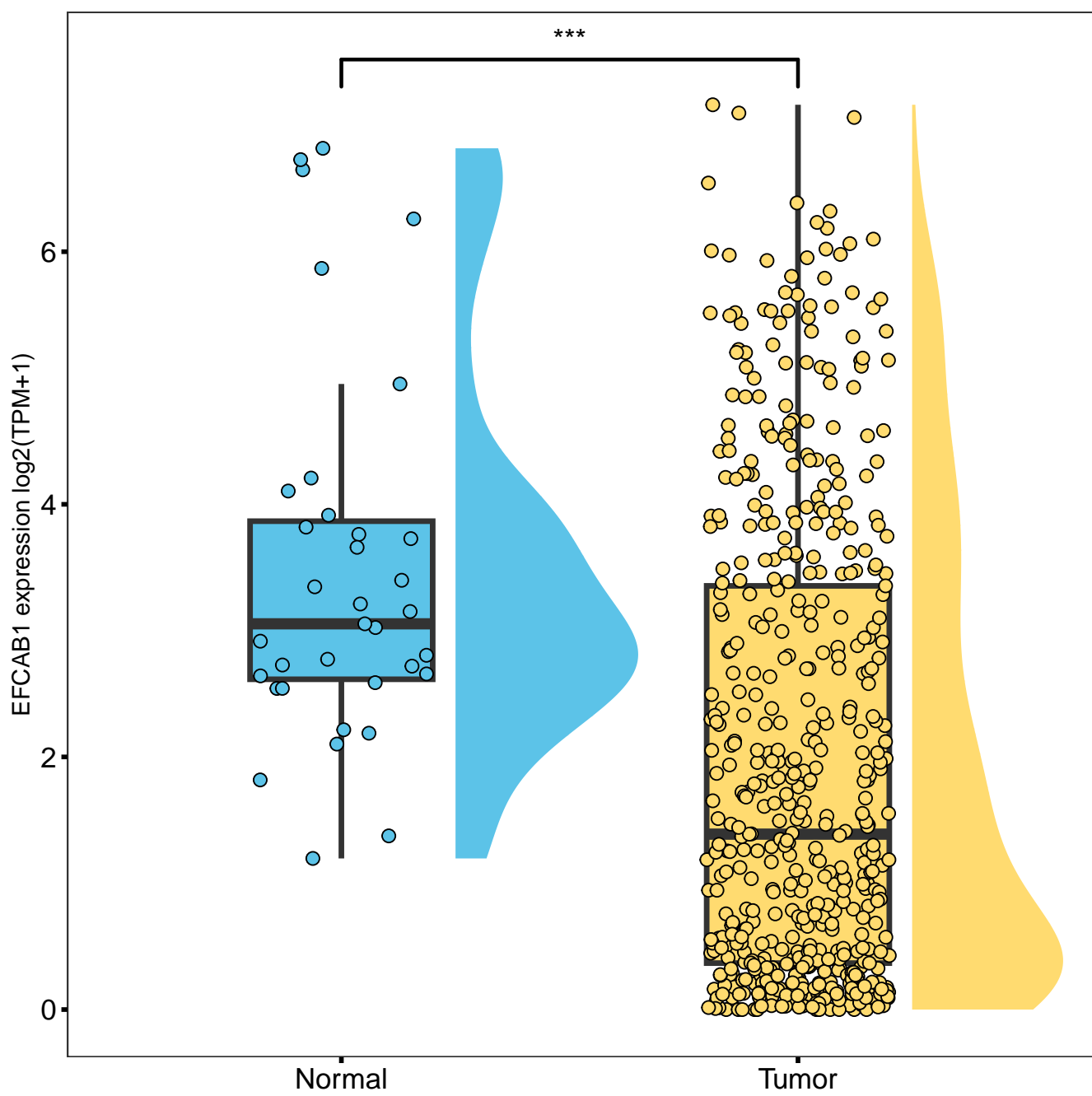

Supplement: Supplementary file 1 [file DataSheet1.zip › supplementary file/supplementary file 1/EFCAB1_boxplot.pdf]

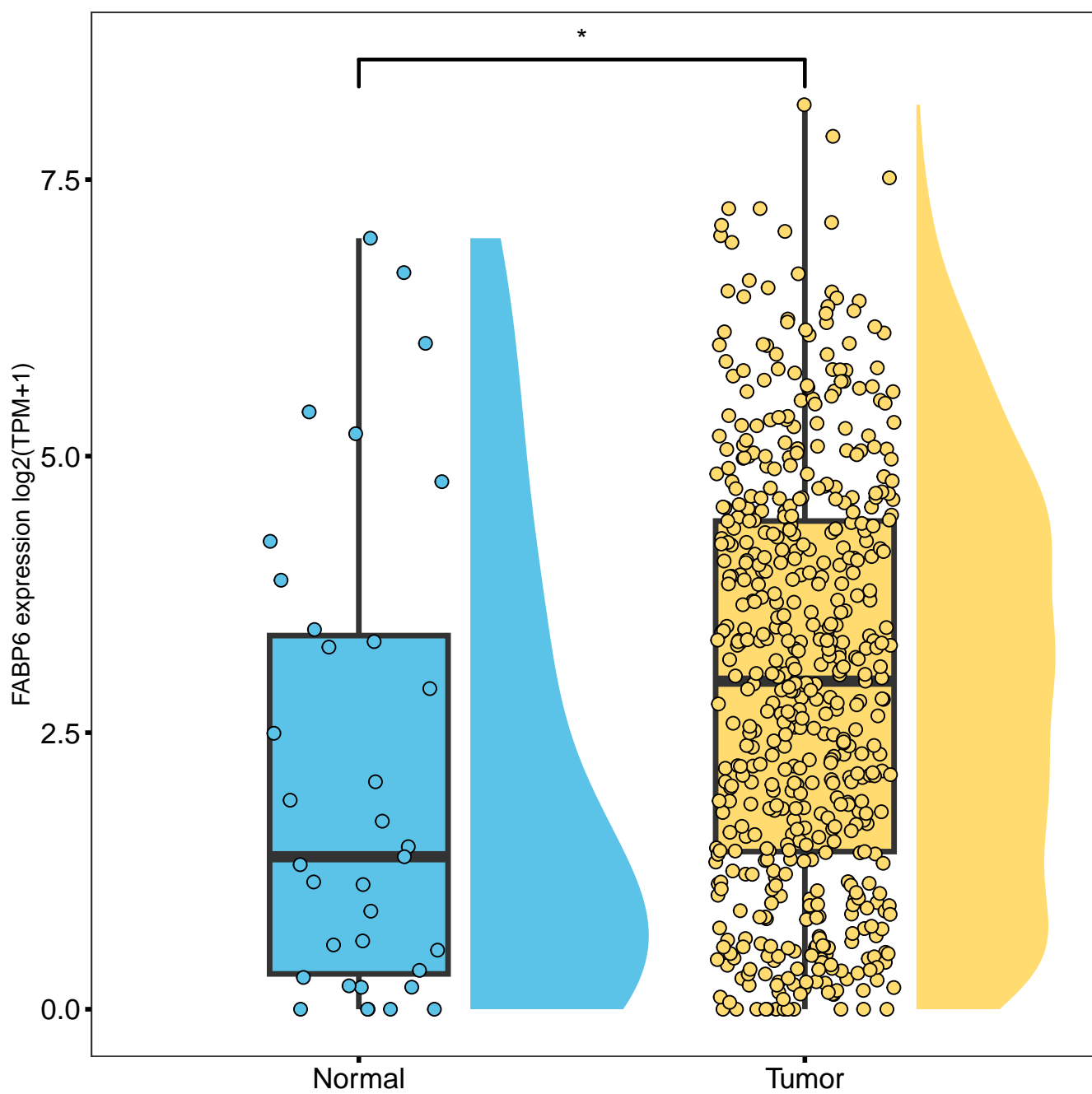

Supplement: Supplementary file 1 [file DataSheet1.zip › supplementary file/supplementary file 1/FABP6_boxplot.pdf]

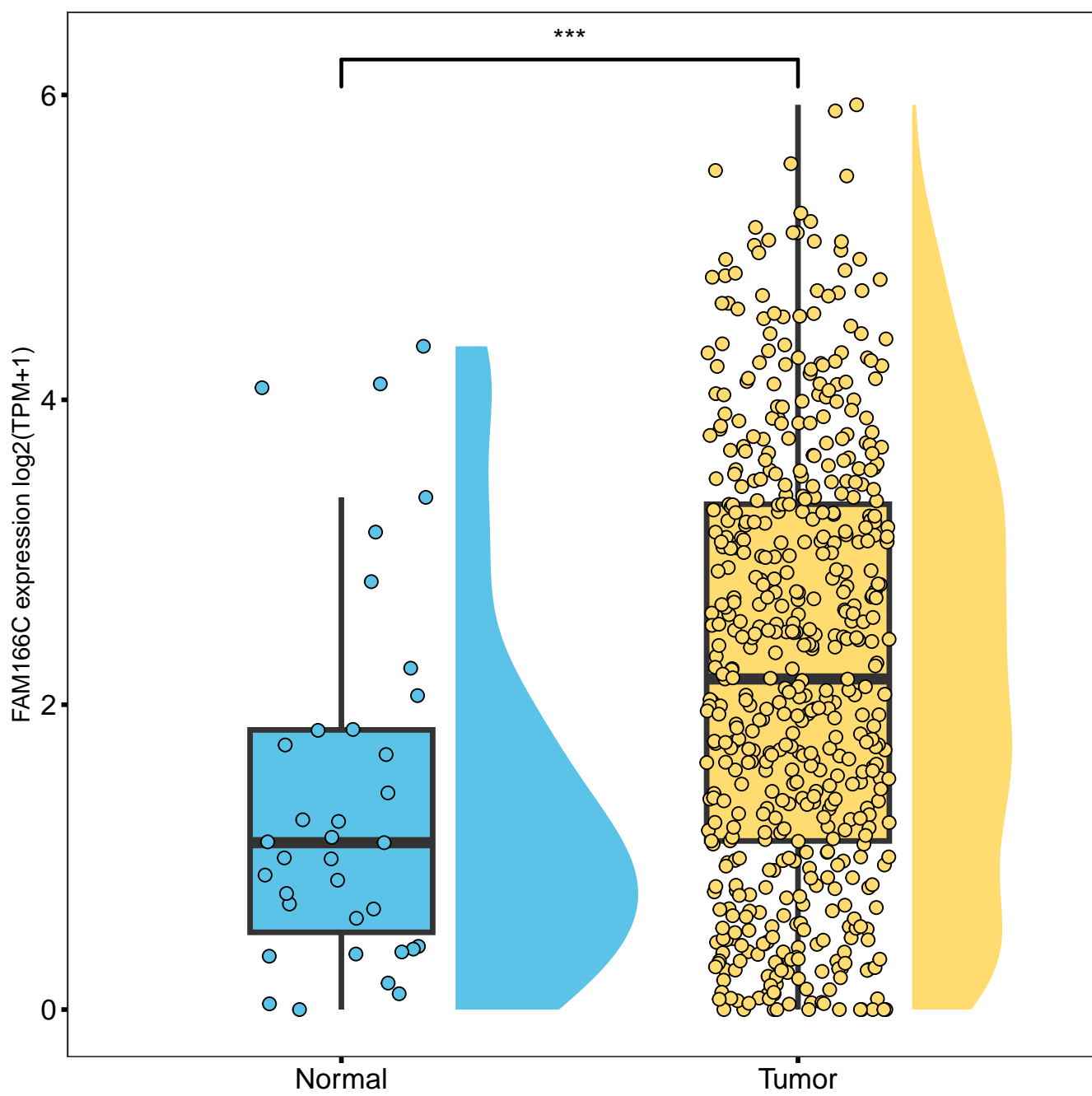

Supplement: Supplementary file 1 [file DataSheet1.zip › supplementary file/supplementary file 1/FAM166C_boxplot.pdf]

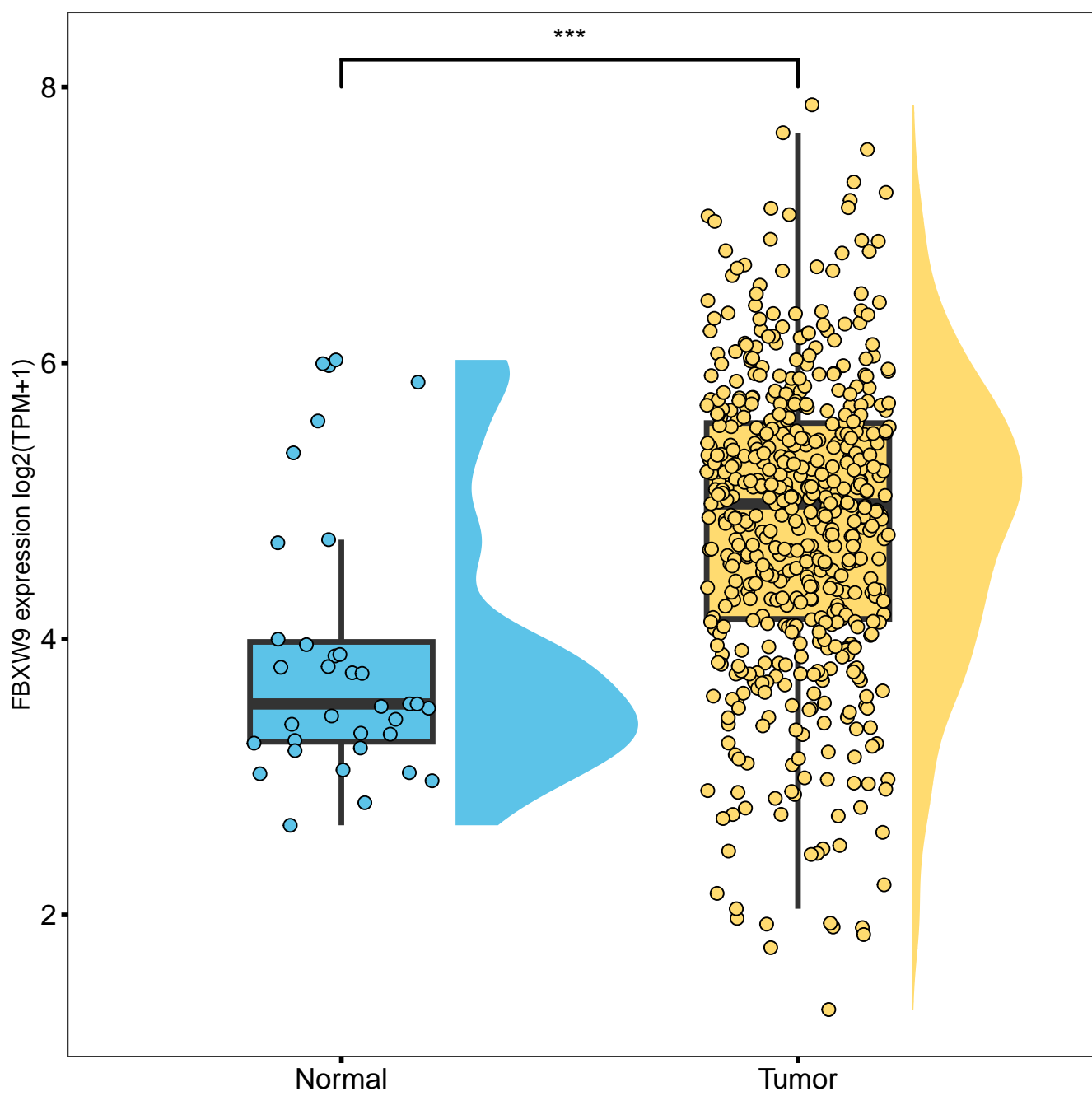

Supplement: Supplementary file 1 [file DataSheet1.zip › supplementary file/supplementary file 1/FBXW9_boxplot.pdf]

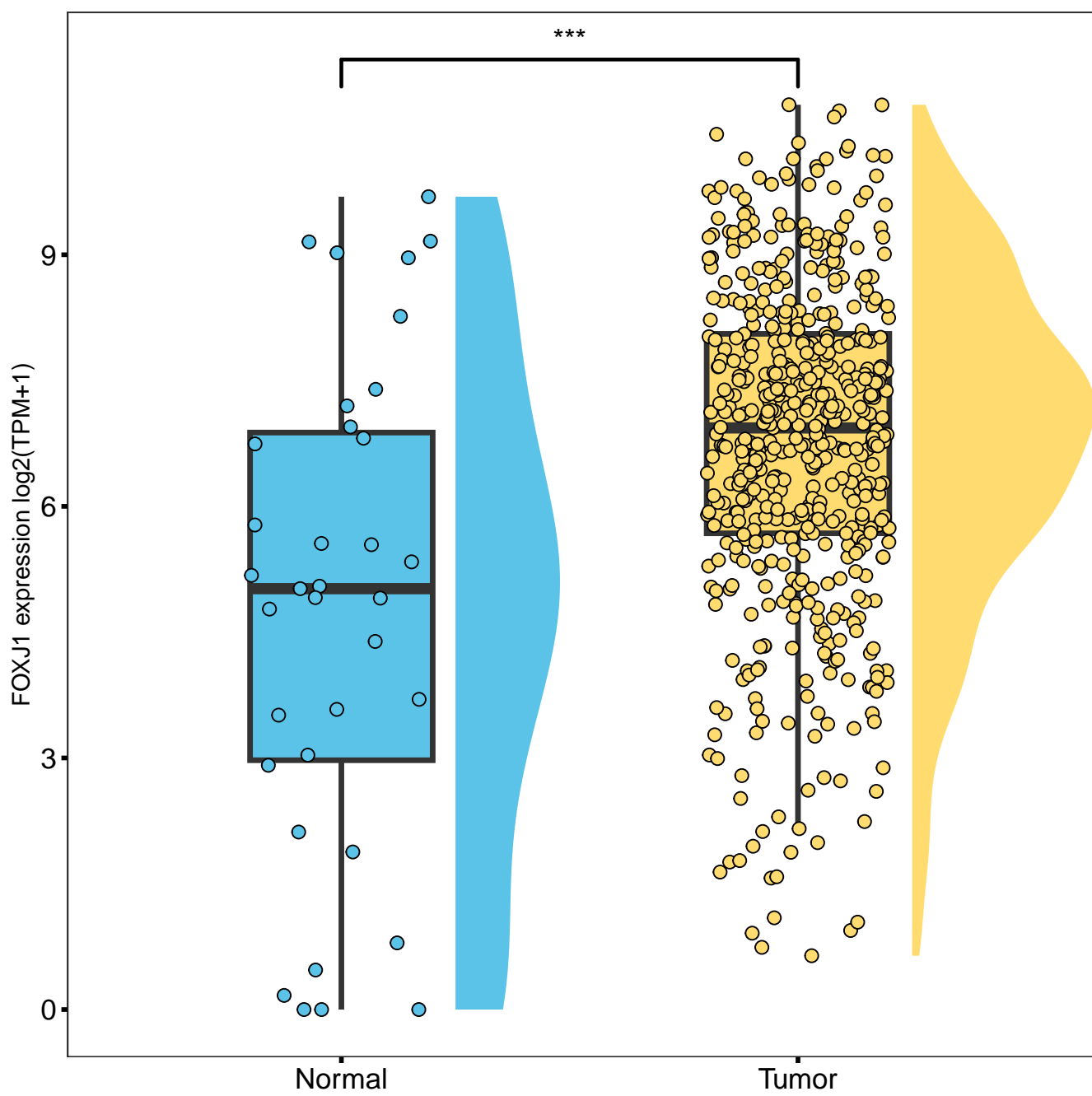

Supplement: Supplementary file 1 [file DataSheet1.zip › supplementary file/supplementary file 1/FOXJ1_boxplot.pdf]

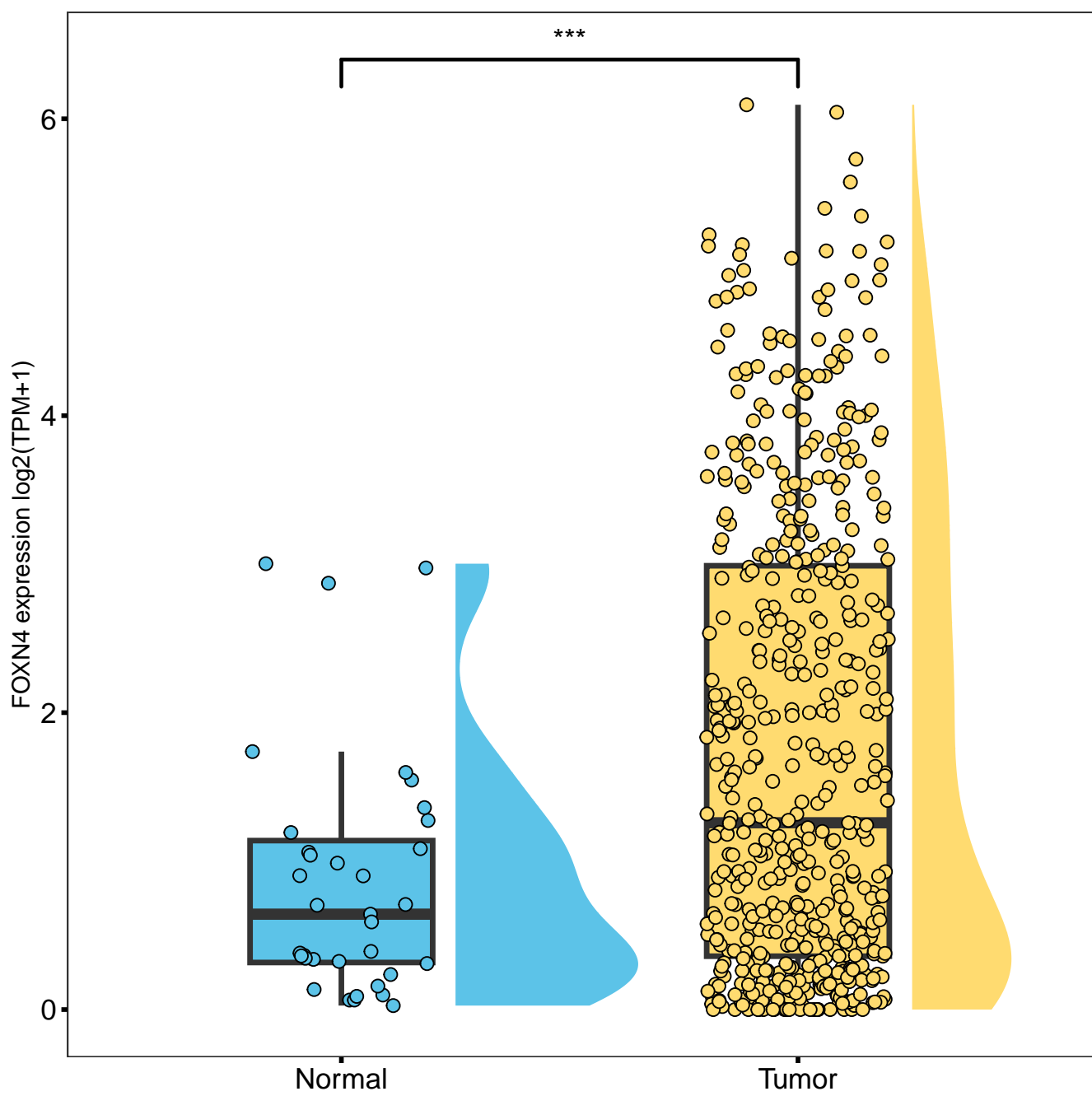

Supplement: Supplementary file 1 [file DataSheet1.zip › supplementary file/supplementary file 1/FOXN4_boxplot.pdf]

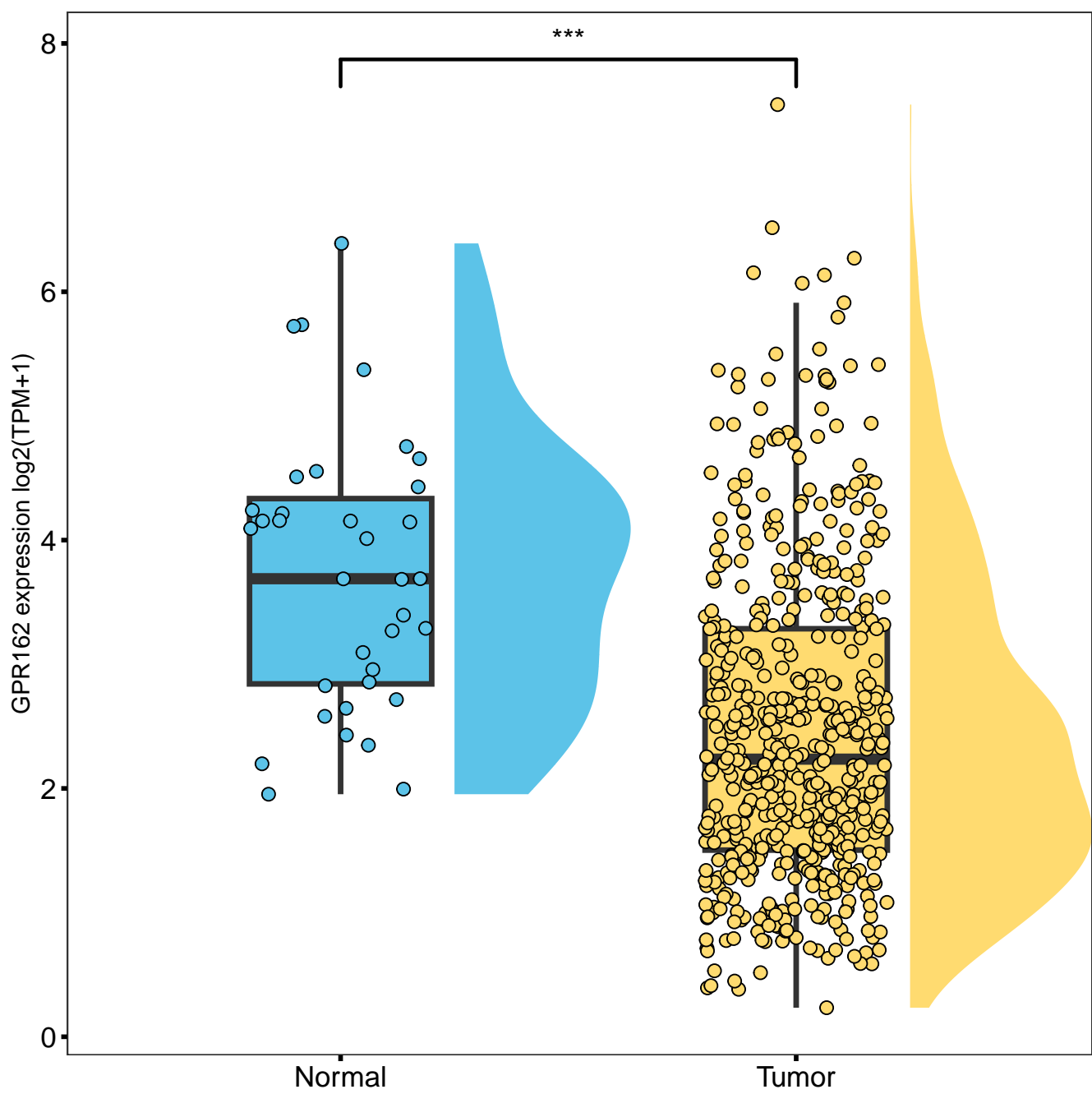

Supplement: Supplementary file 1 [file DataSheet1.zip › supplementary file/supplementary file 1/GPR162_boxplot.pdf]

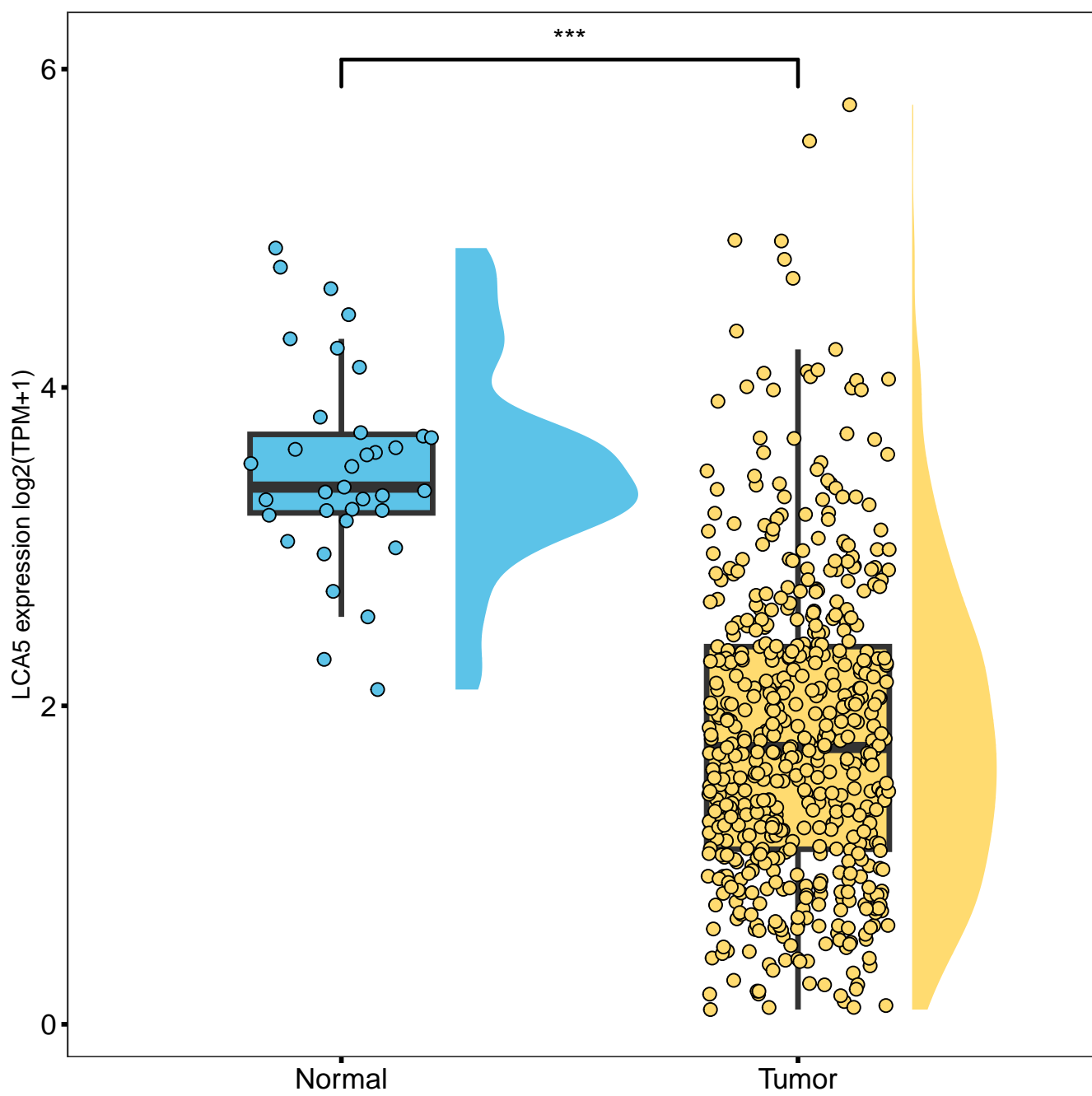

Supplement: Supplementary file 1 [file DataSheet1.zip › supplementary file/supplementary file 1/LCA5_boxplot.pdf]

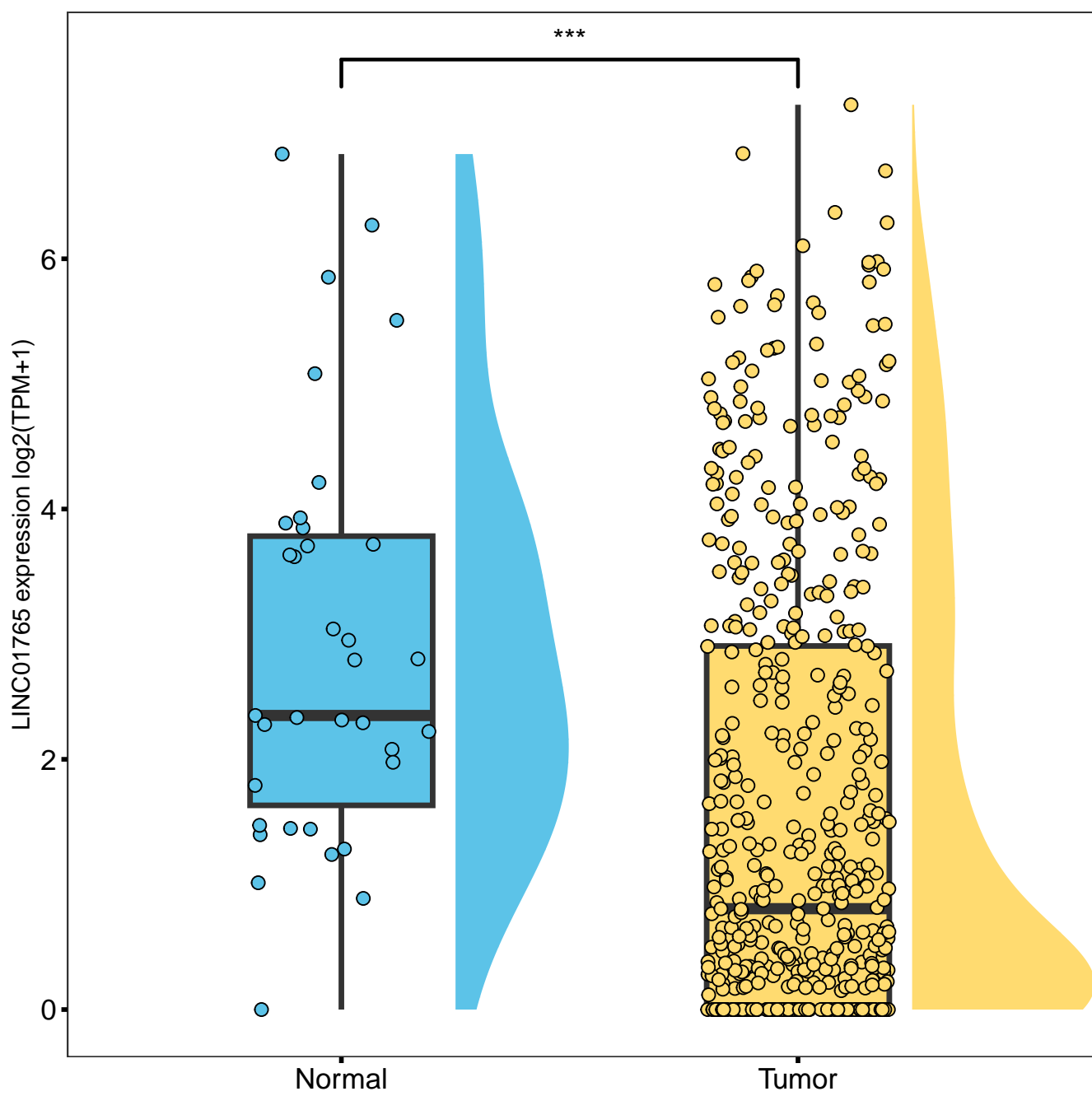

Supplement: Supplementary file 1 [file DataSheet1.zip › supplementary file/supplementary file 1/LINC01765_boxplot.pdf]

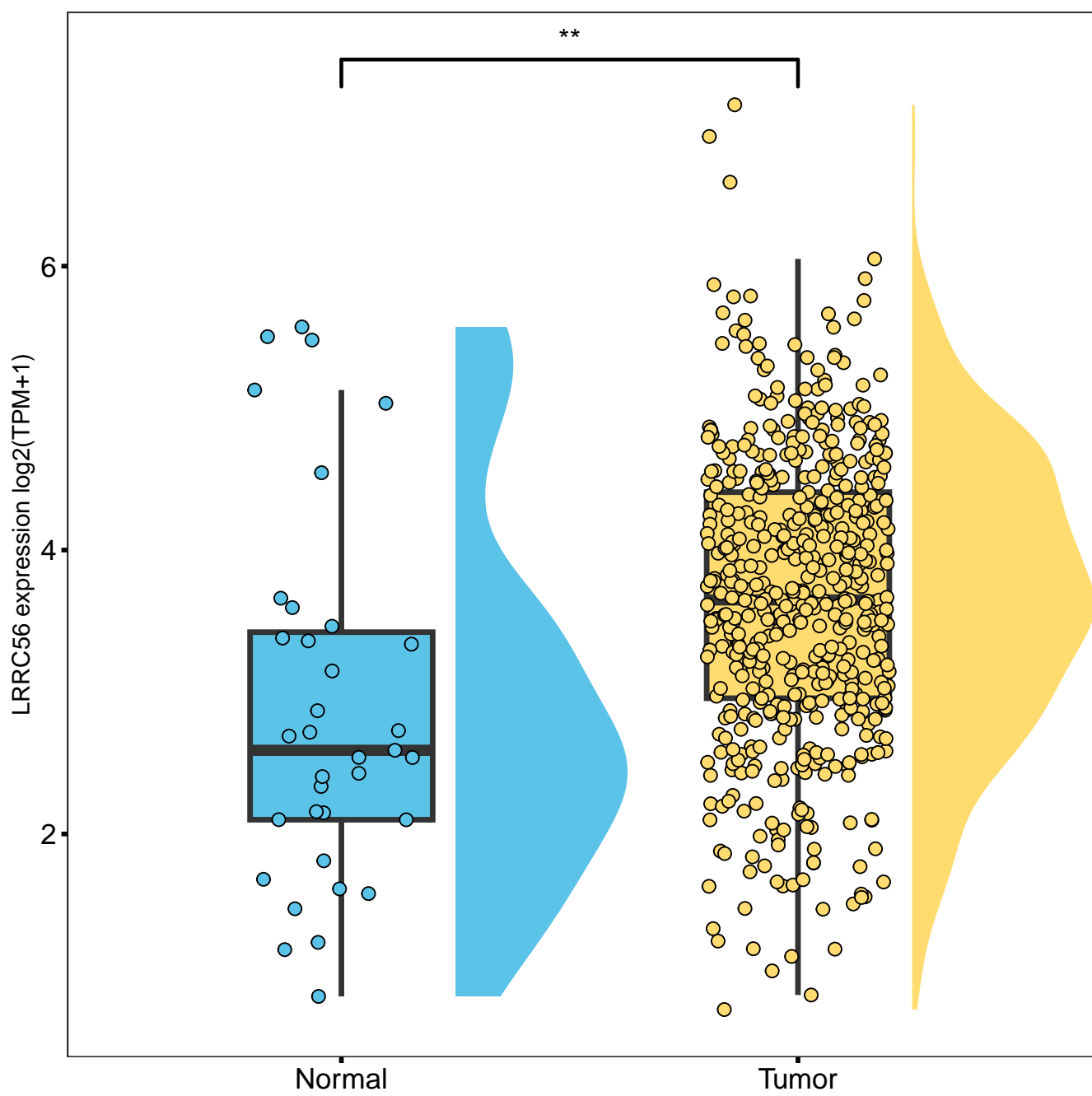

Supplement: Supplementary file 1 [file DataSheet1.zip › supplementary file/supplementary file 1/LRRC56_boxplot.pdf]

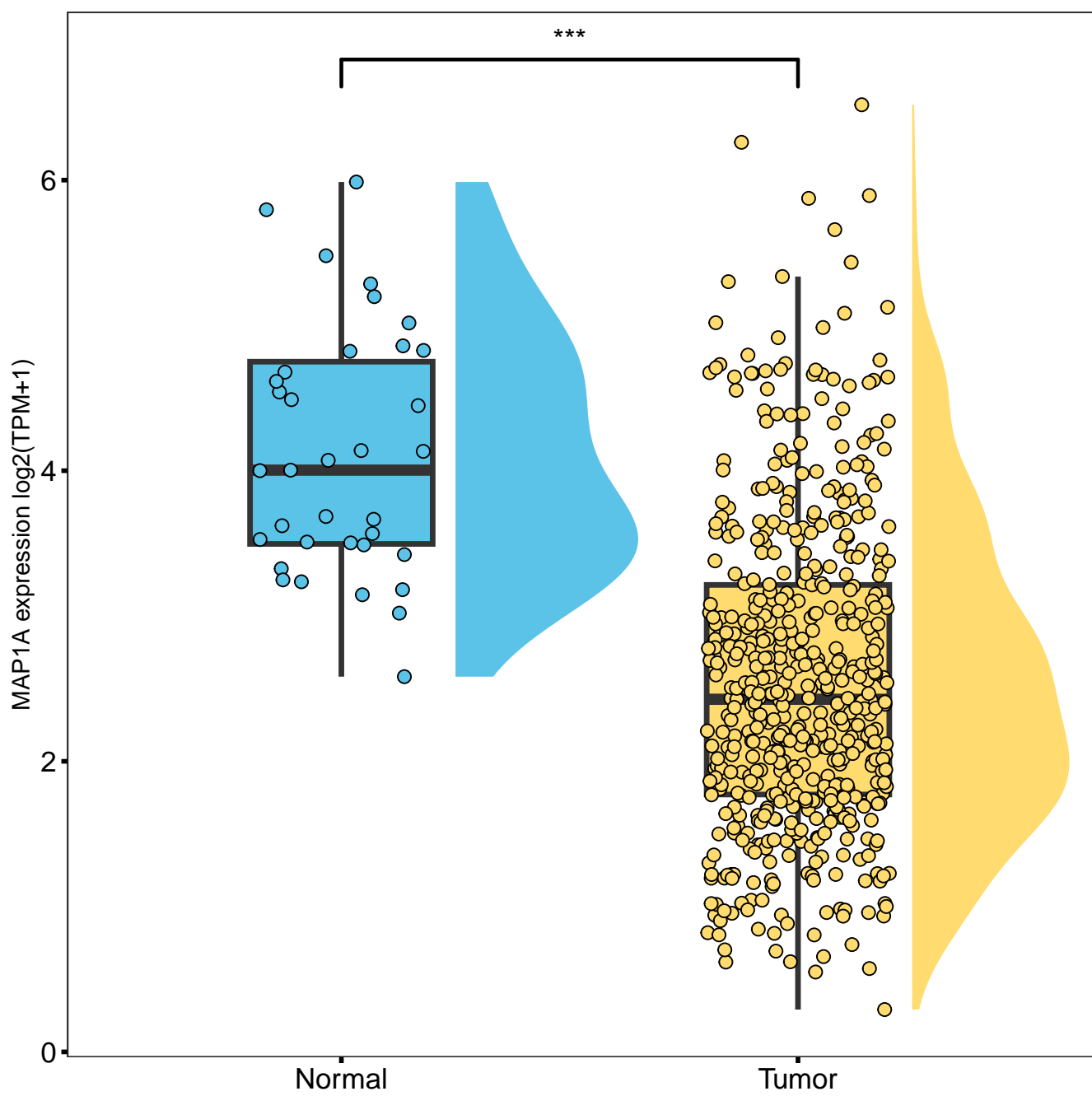

Supplement: Supplementary file 1 [file DataSheet1.zip › supplementary file/supplementary file 1/MAP1A_boxplot.pdf]

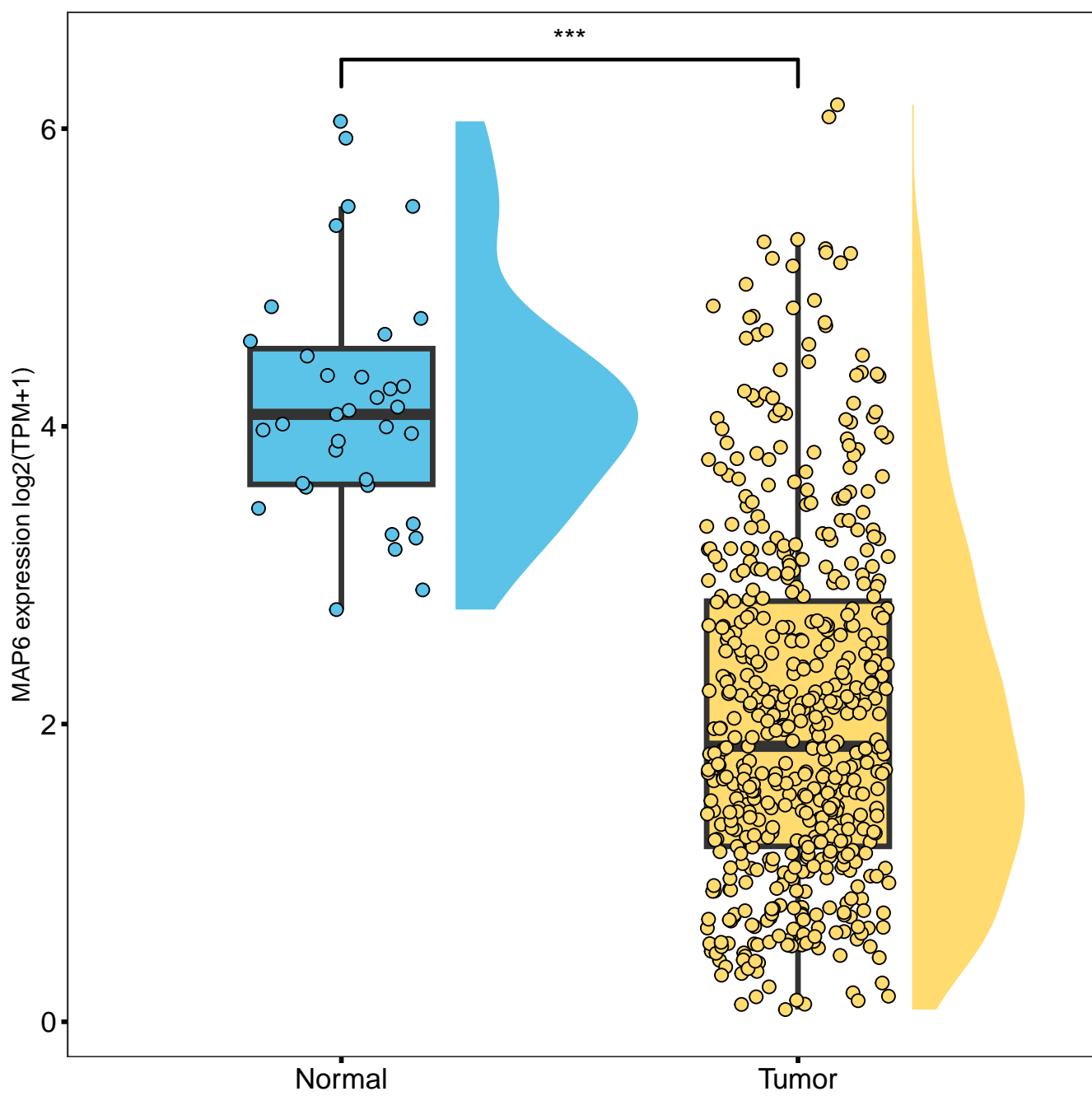

Supplement: Supplementary file 1 [file DataSheet1.zip › supplementary file/supplementary file 1/MAP6_boxplot.pdf]

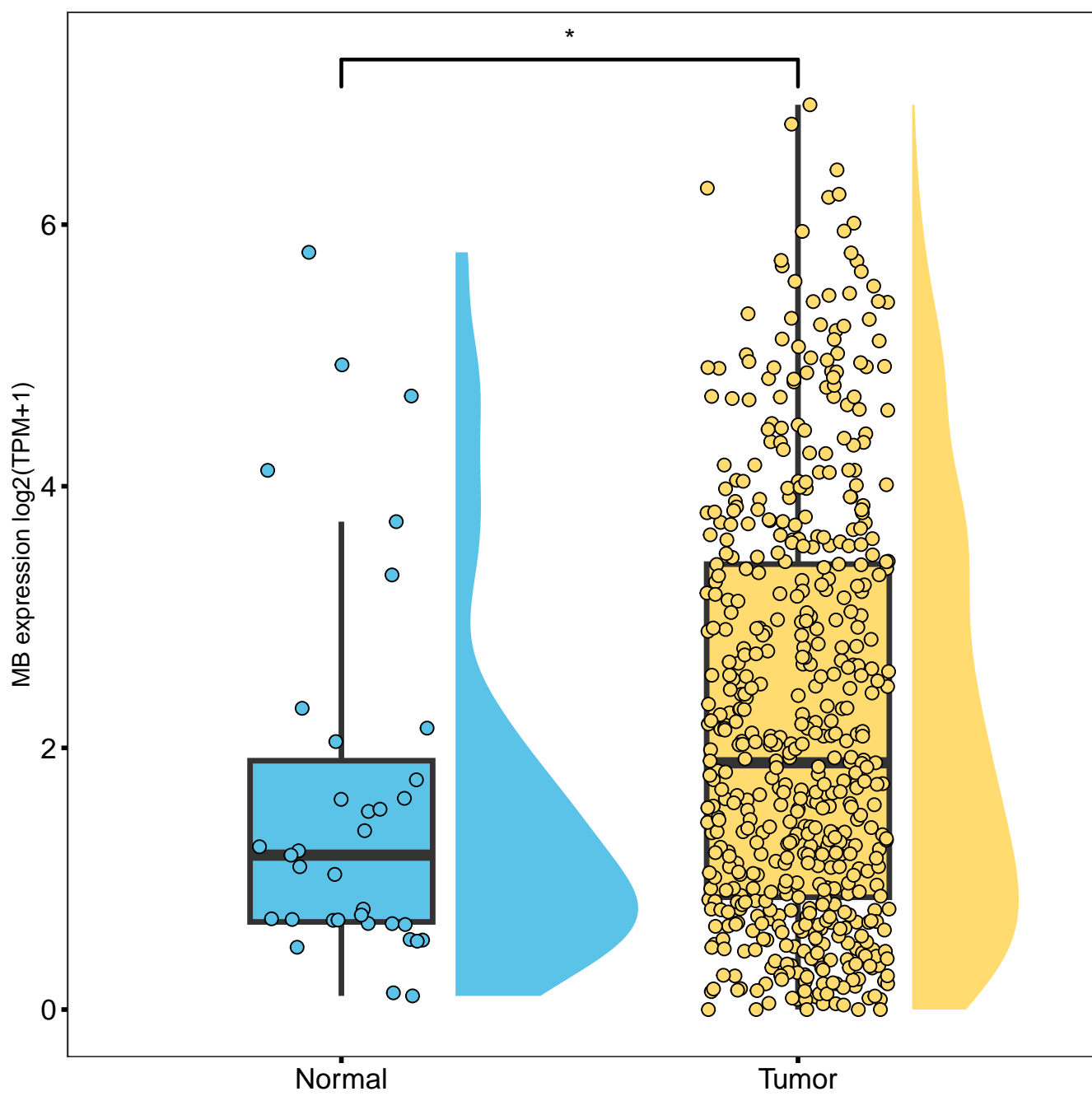

Supplement: Supplementary file 1 [file DataSheet1.zip › supplementary file/supplementary file 1/MB_boxplot.pdf]

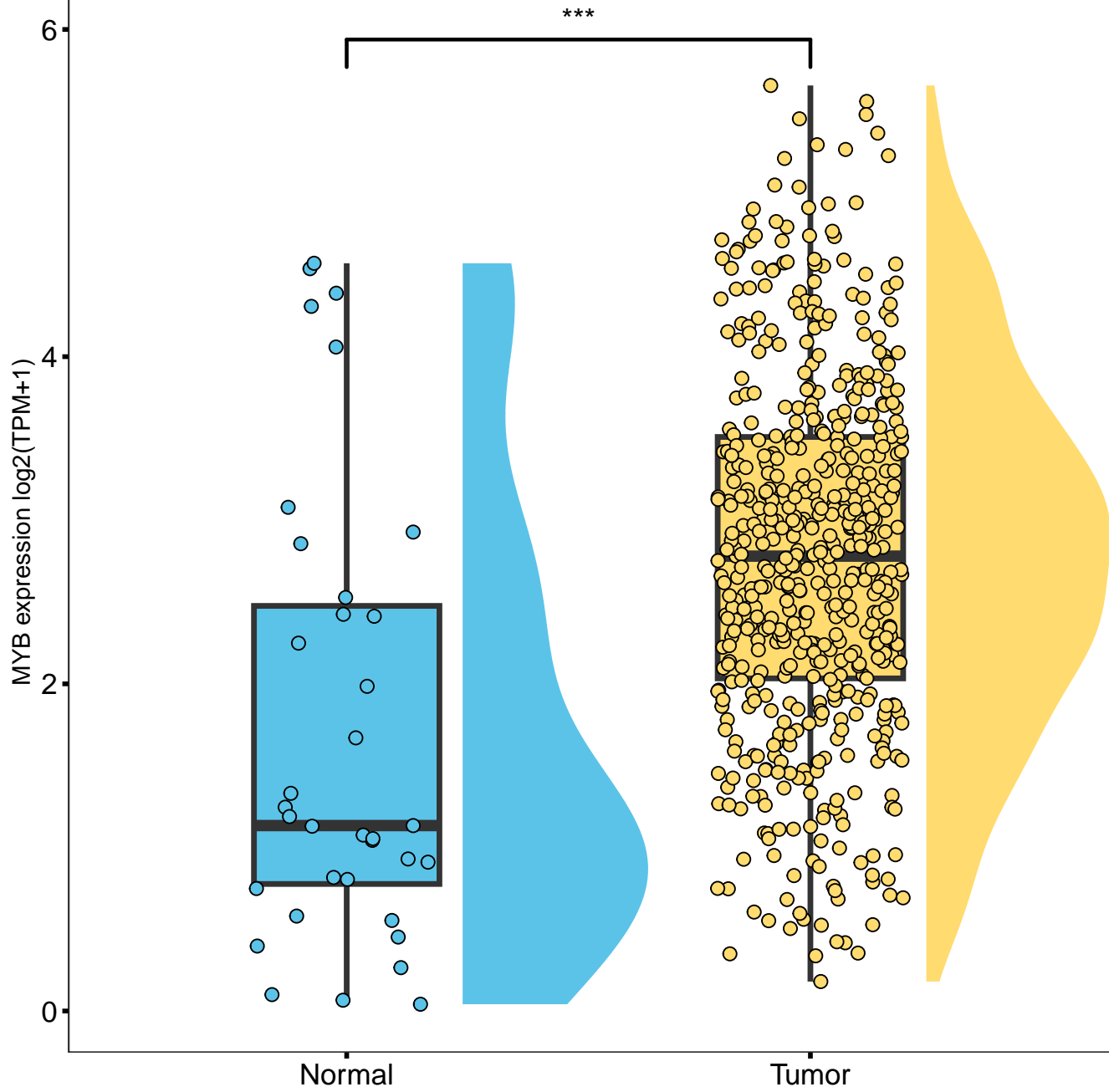

Supplement: Supplementary file 1 [file DataSheet1.zip › supplementary file/supplementary file 1/MYB_boxplot.pdf]

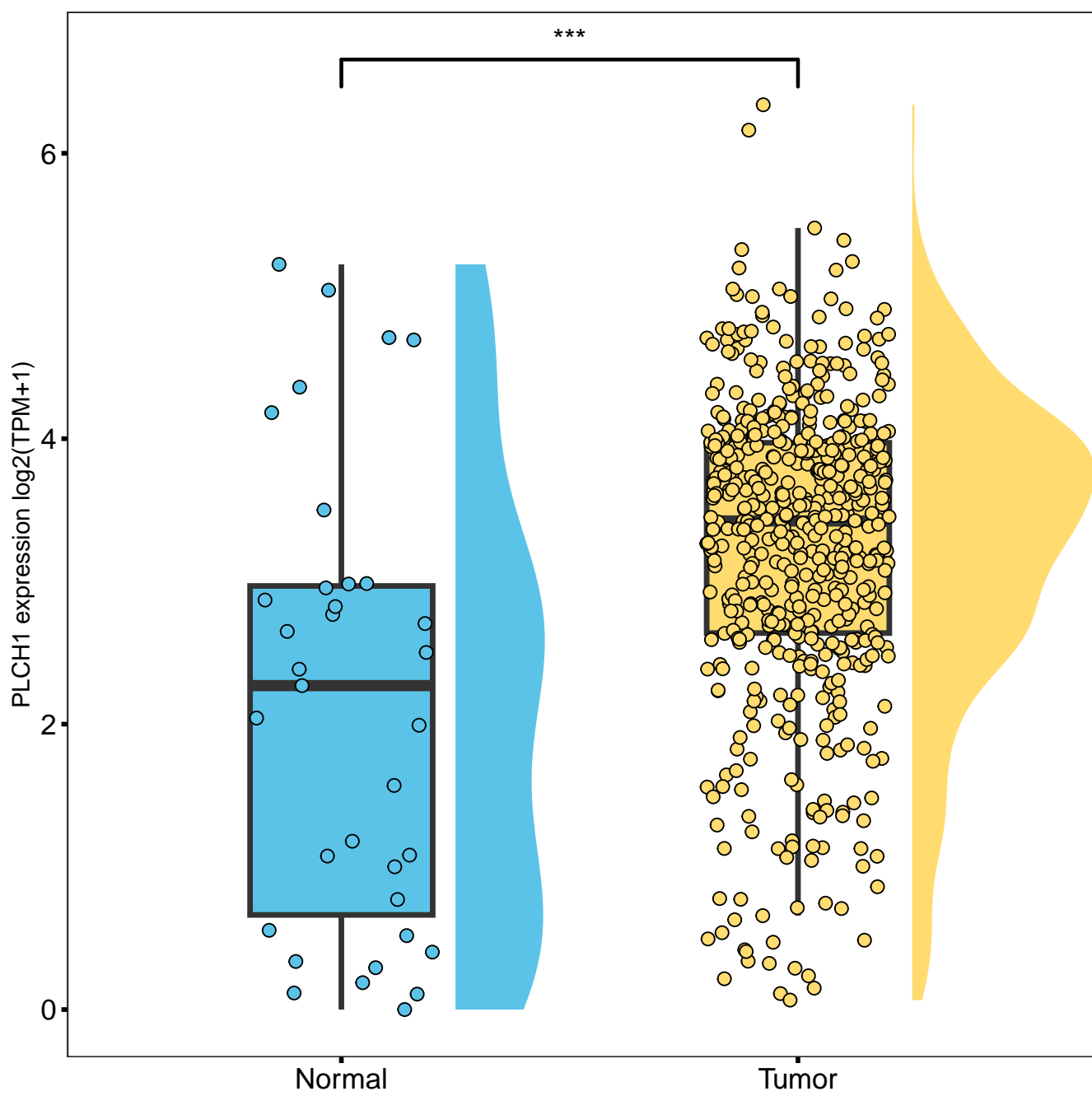

Supplement: Supplementary file 1 [file DataSheet1.zip › supplementary file/supplementary file 1/PLCH1_boxplot.pdf]

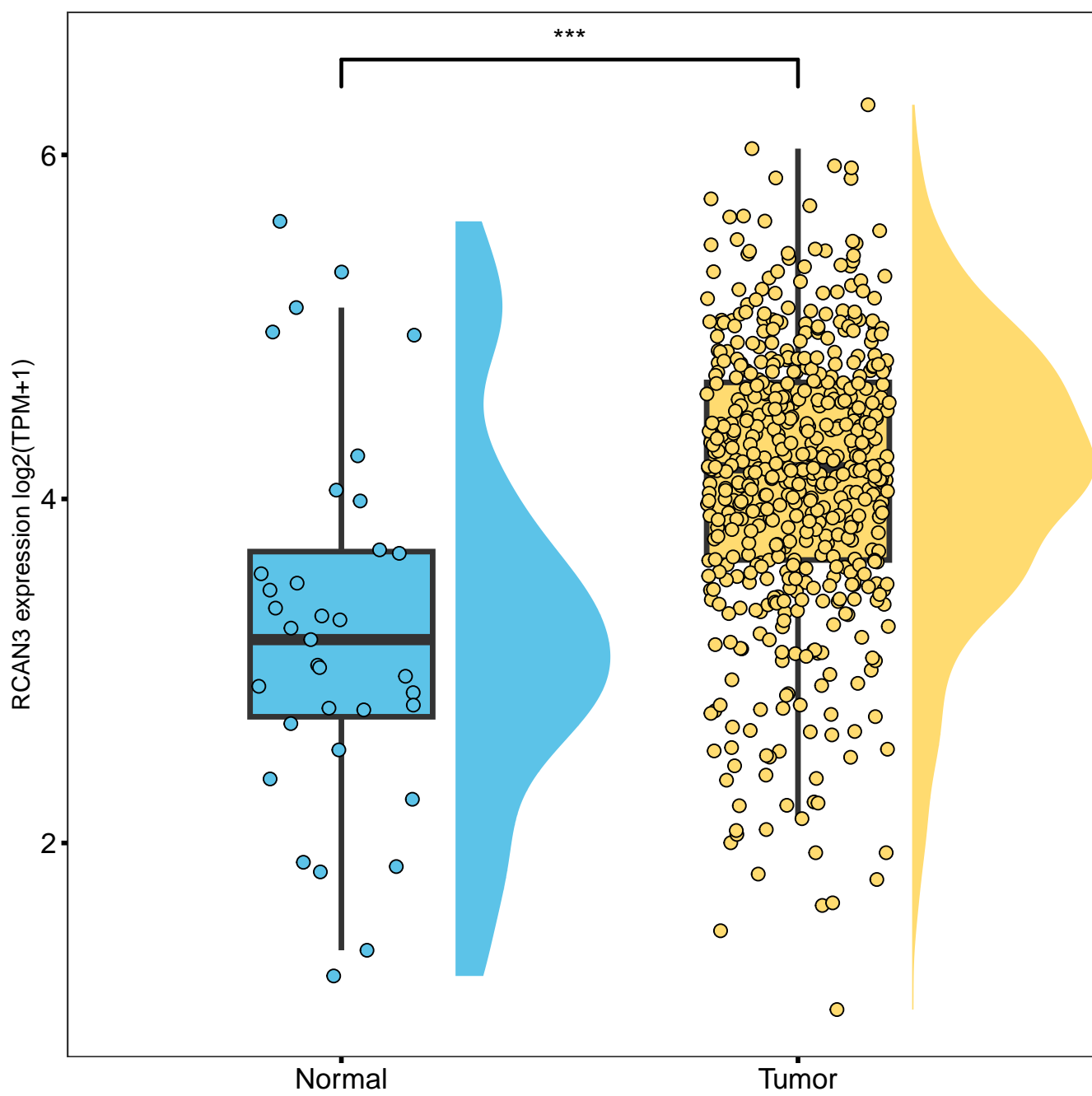

Supplement: Supplementary file 1 [file DataSheet1.zip › supplementary file/supplementary file 1/RCAN3_boxplot.pdf]

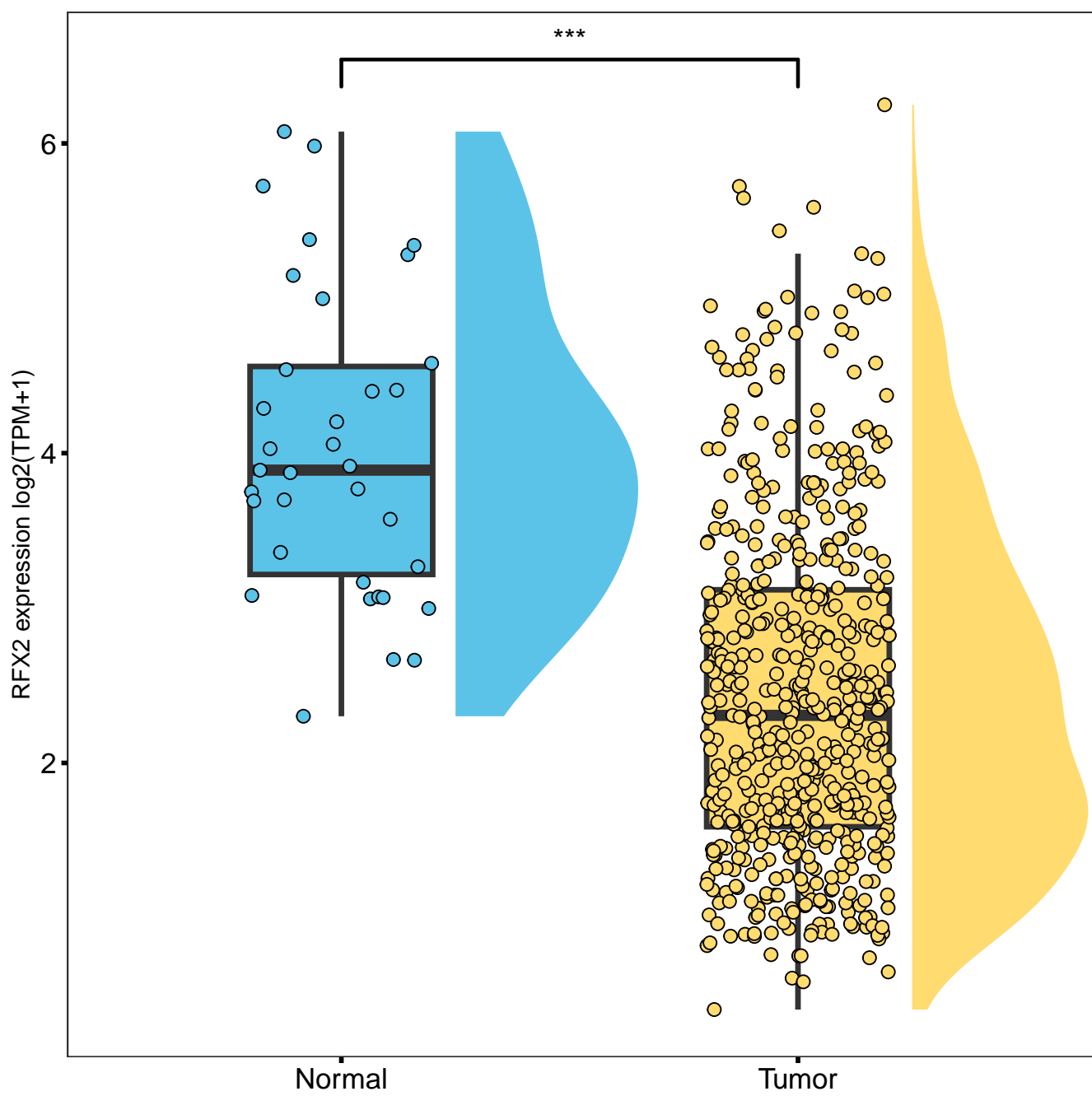

Supplement: Supplementary file 1 [file DataSheet1.zip › supplementary file/supplementary file 1/RFX2_boxplot.pdf]

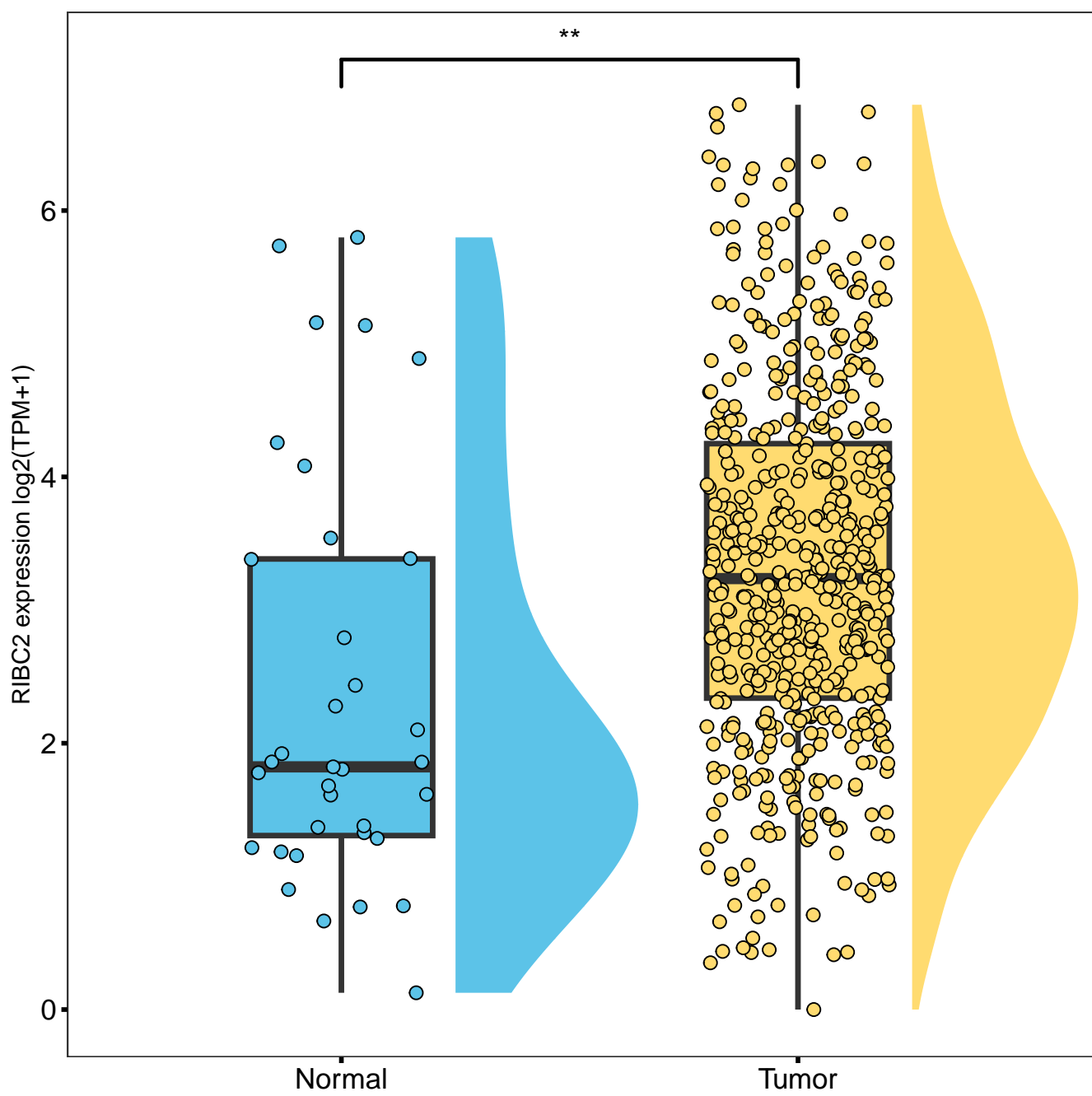

Supplement: Supplementary file 1 [file DataSheet1.zip › supplementary file/supplementary file 1/RIBC2_boxplot.pdf]

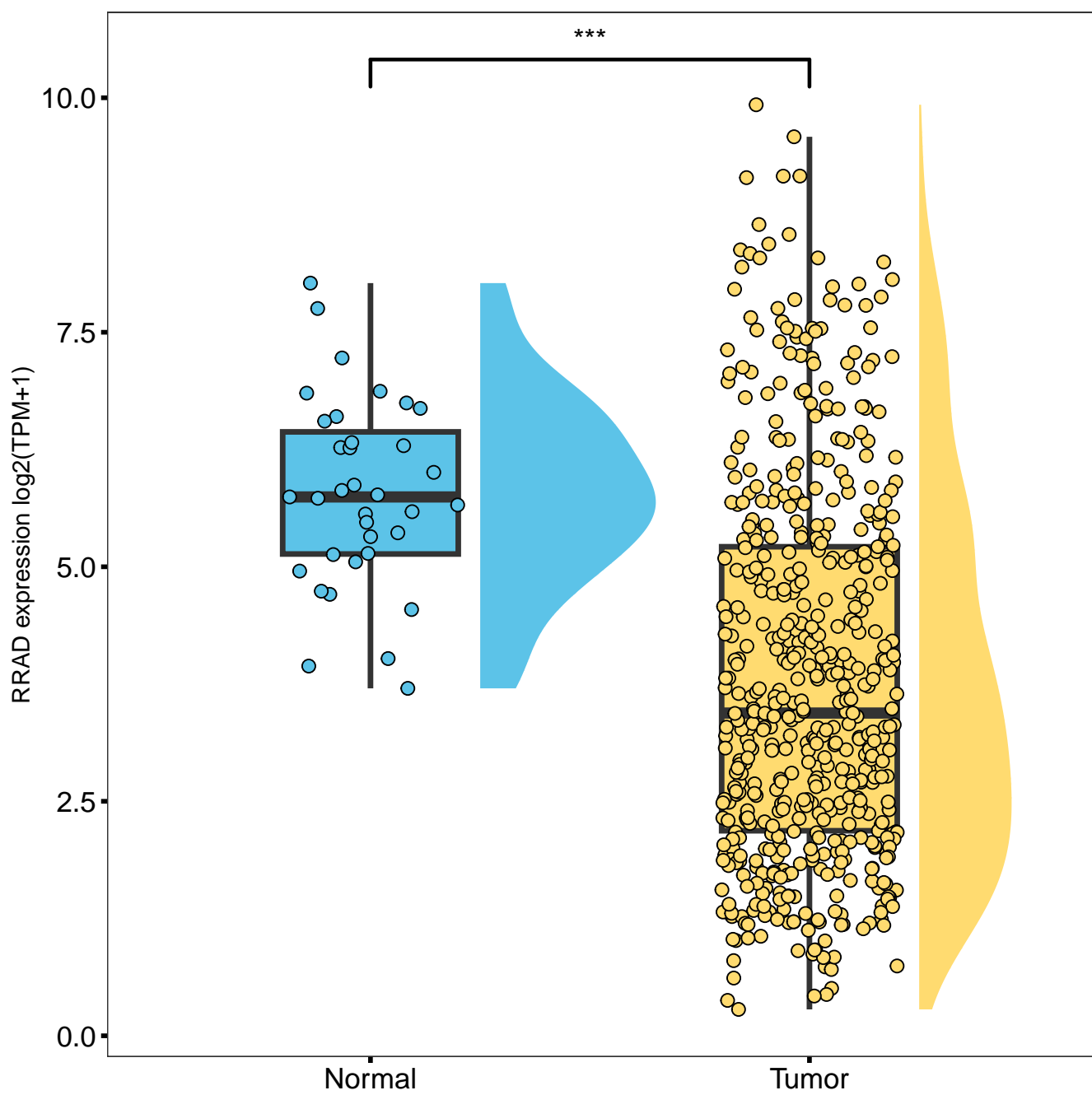

Supplement: Supplementary file 1 [file DataSheet1.zip › supplementary file/supplementary file 1/RRAD_boxplot.pdf]

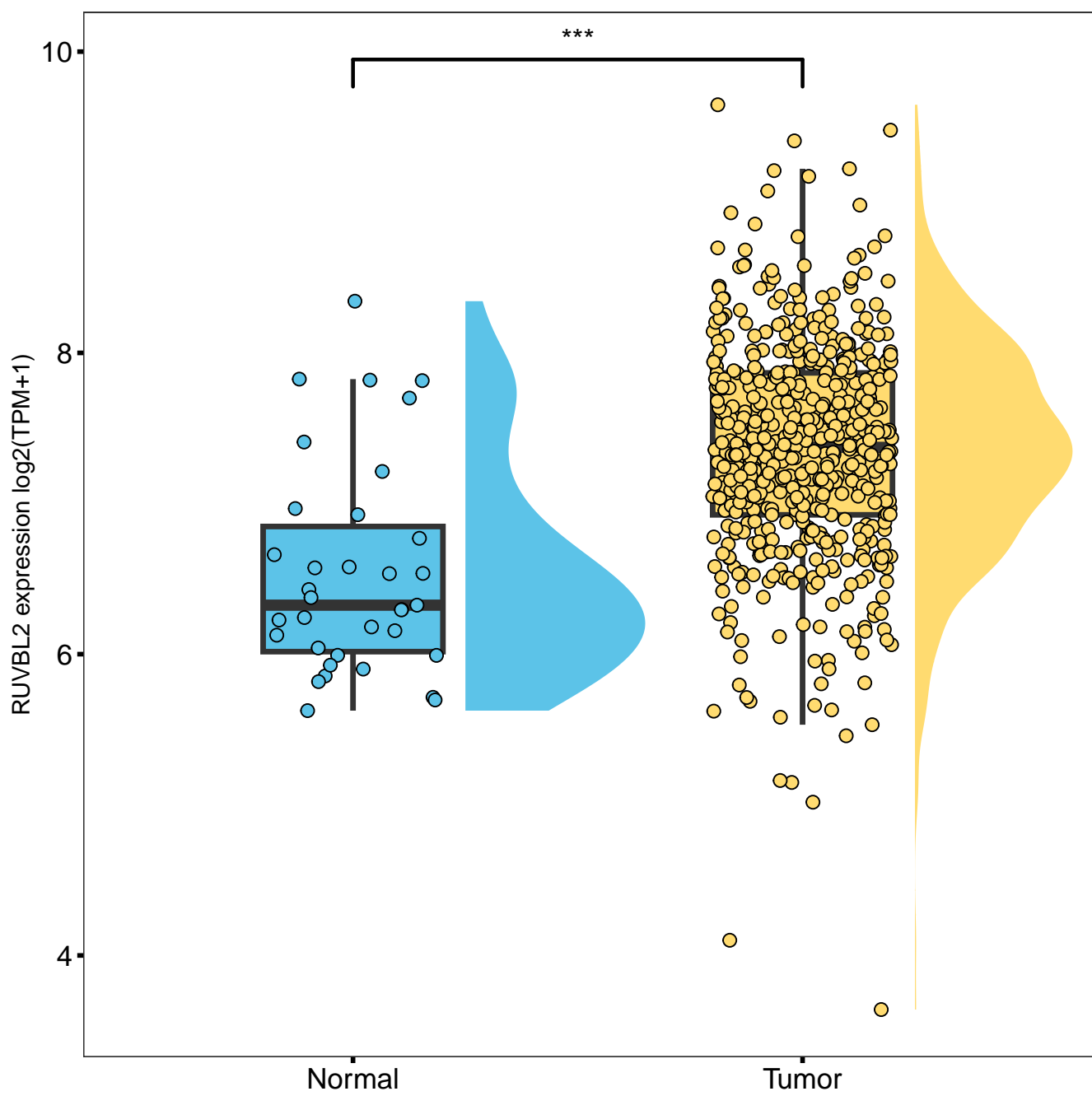

Supplement: Supplementary file 1 [file DataSheet1.zip › supplementary file/supplementary file 1/RUVBL2_boxplot.pdf]

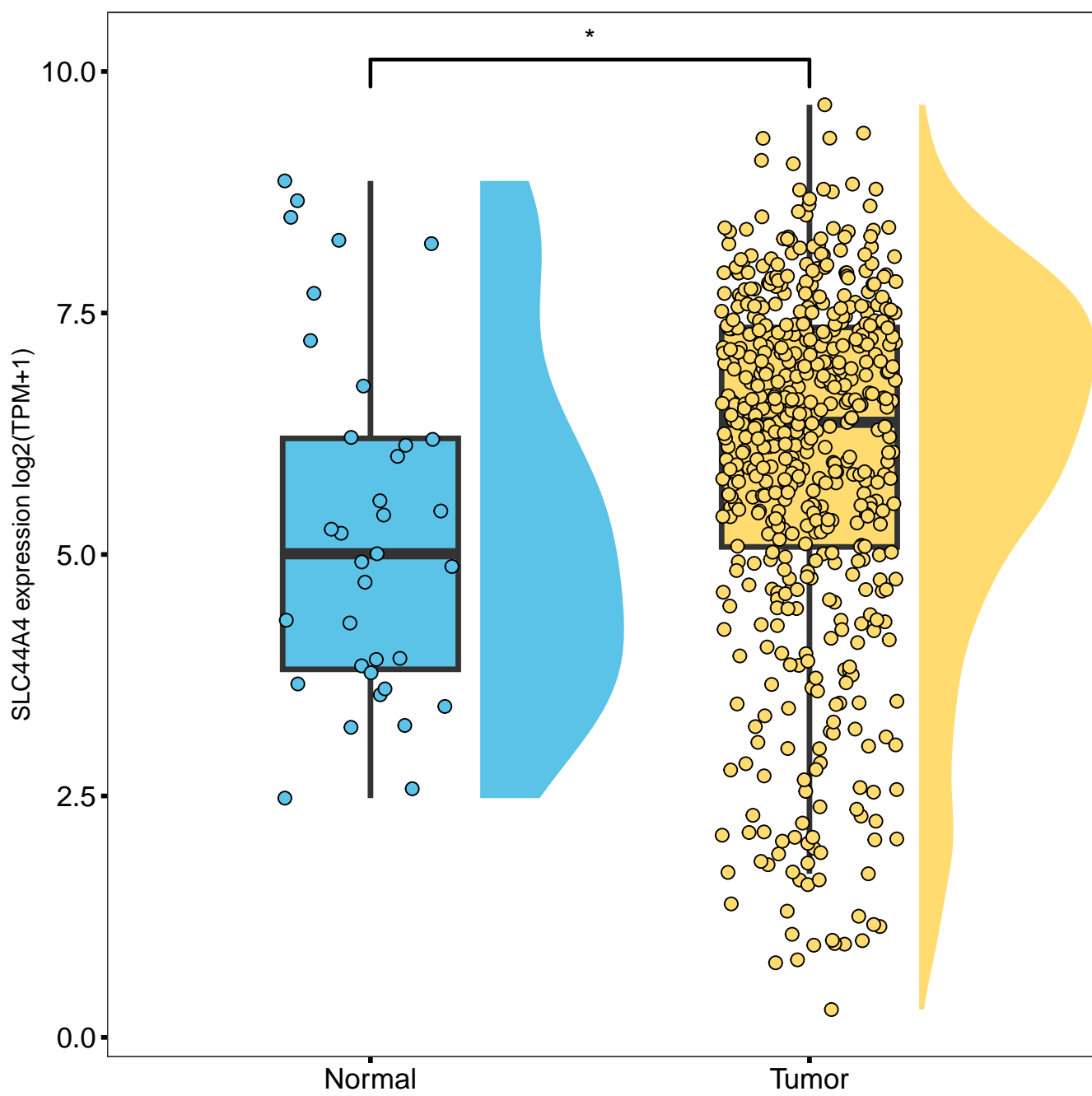

Supplement: Supplementary file 1 [file DataSheet1.zip › supplementary file/supplementary file 1/SLC44A4_boxplot.pdf]

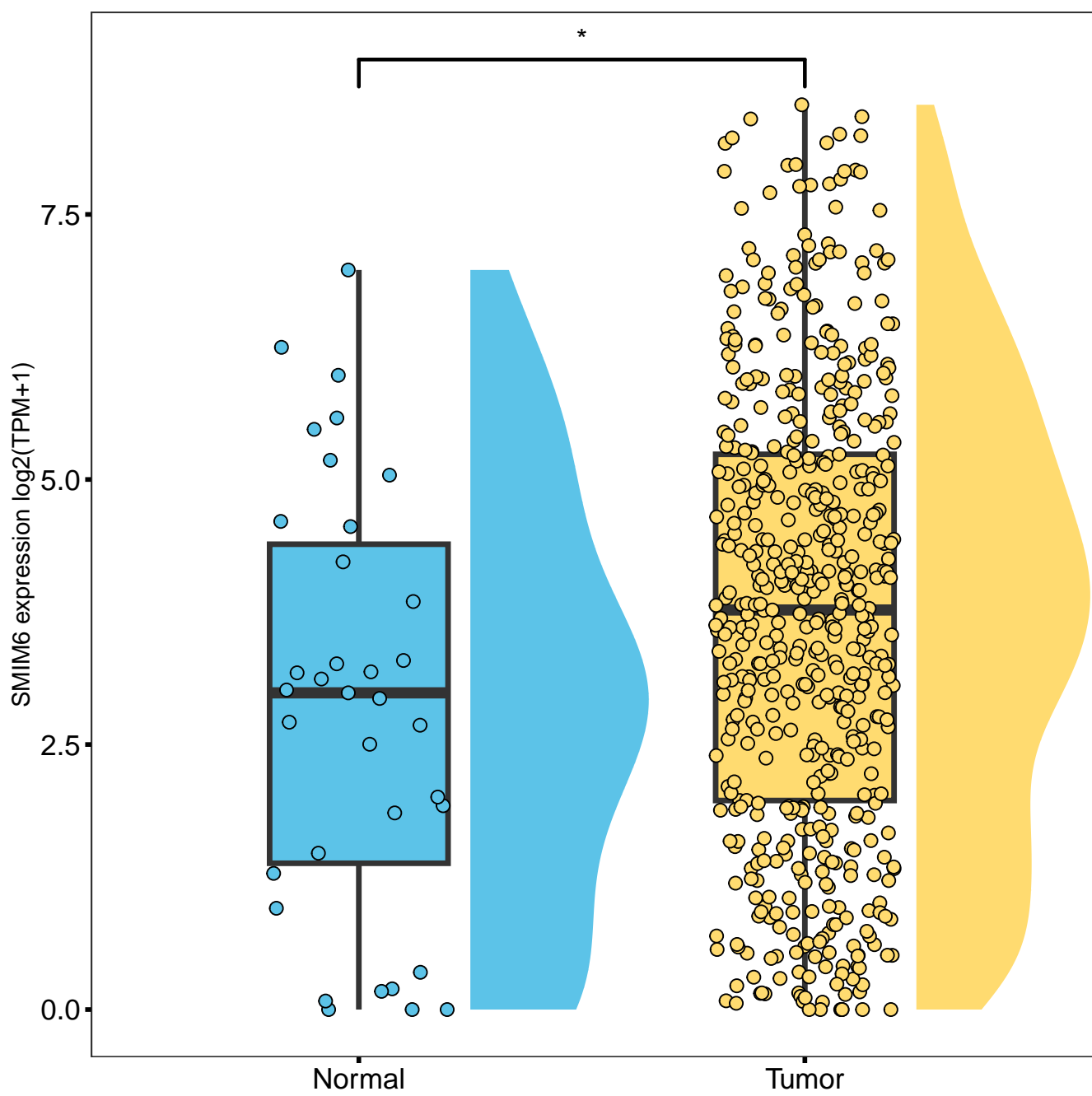

Supplement: Supplementary file 1 [file DataSheet1.zip › supplementary file/supplementary file 1/SMIM6_boxplot.pdf]

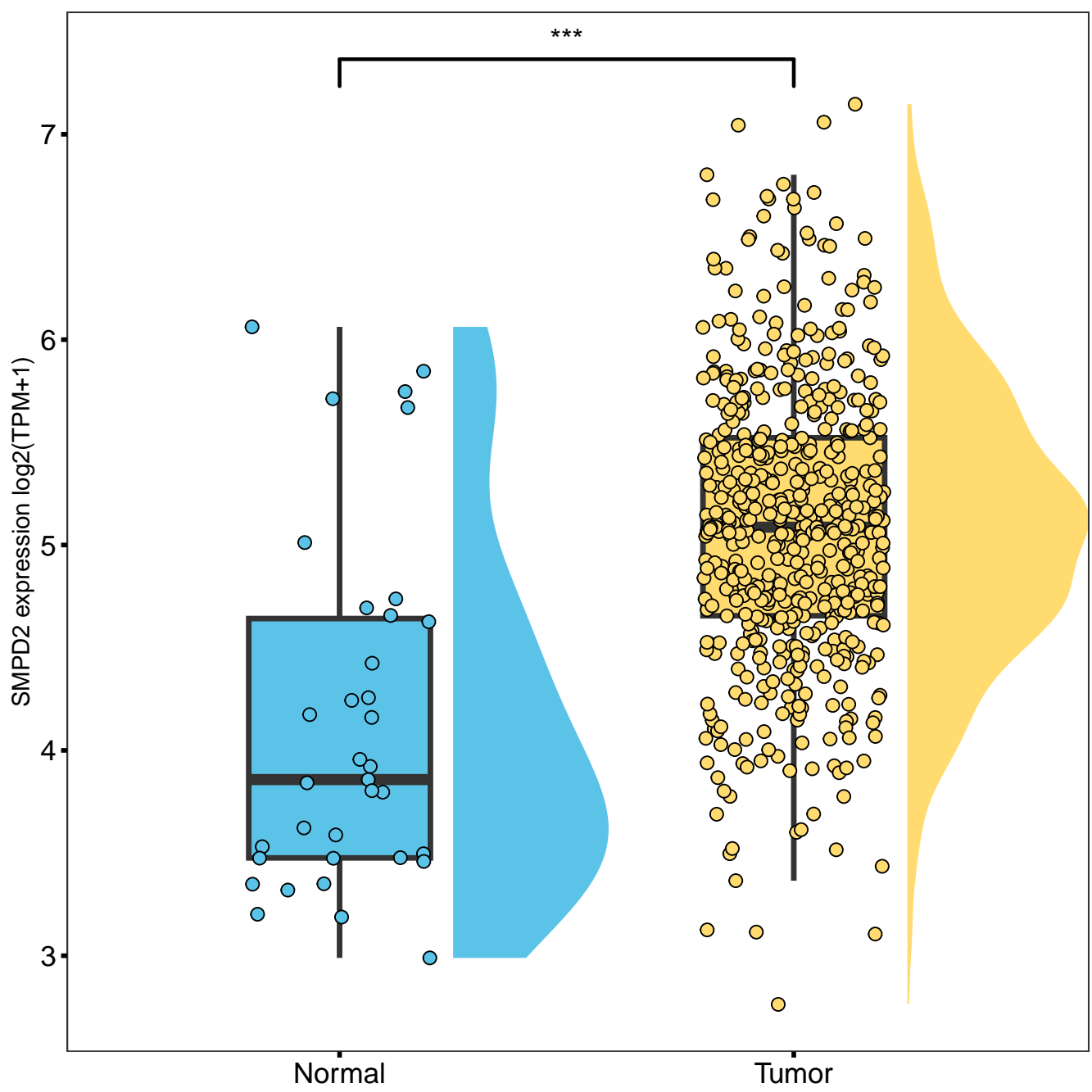

Supplement: Supplementary file 1 [file DataSheet1.zip › supplementary file/supplementary file 1/SMPD2_boxplot.pdf]

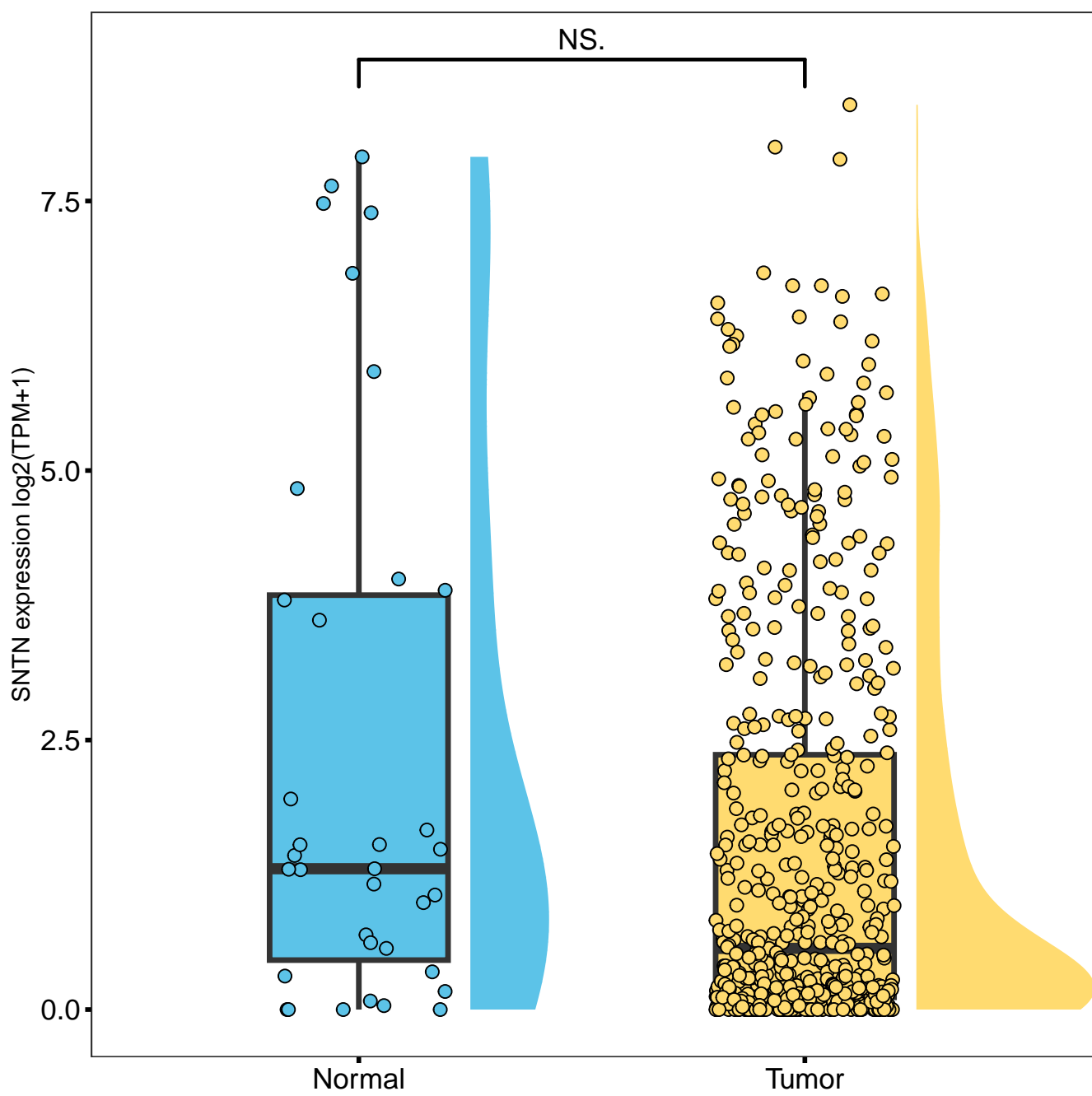

Supplement: Supplementary file 1 [file DataSheet1.zip › supplementary file/supplementary file 1/SNTN_boxplot.pdf]

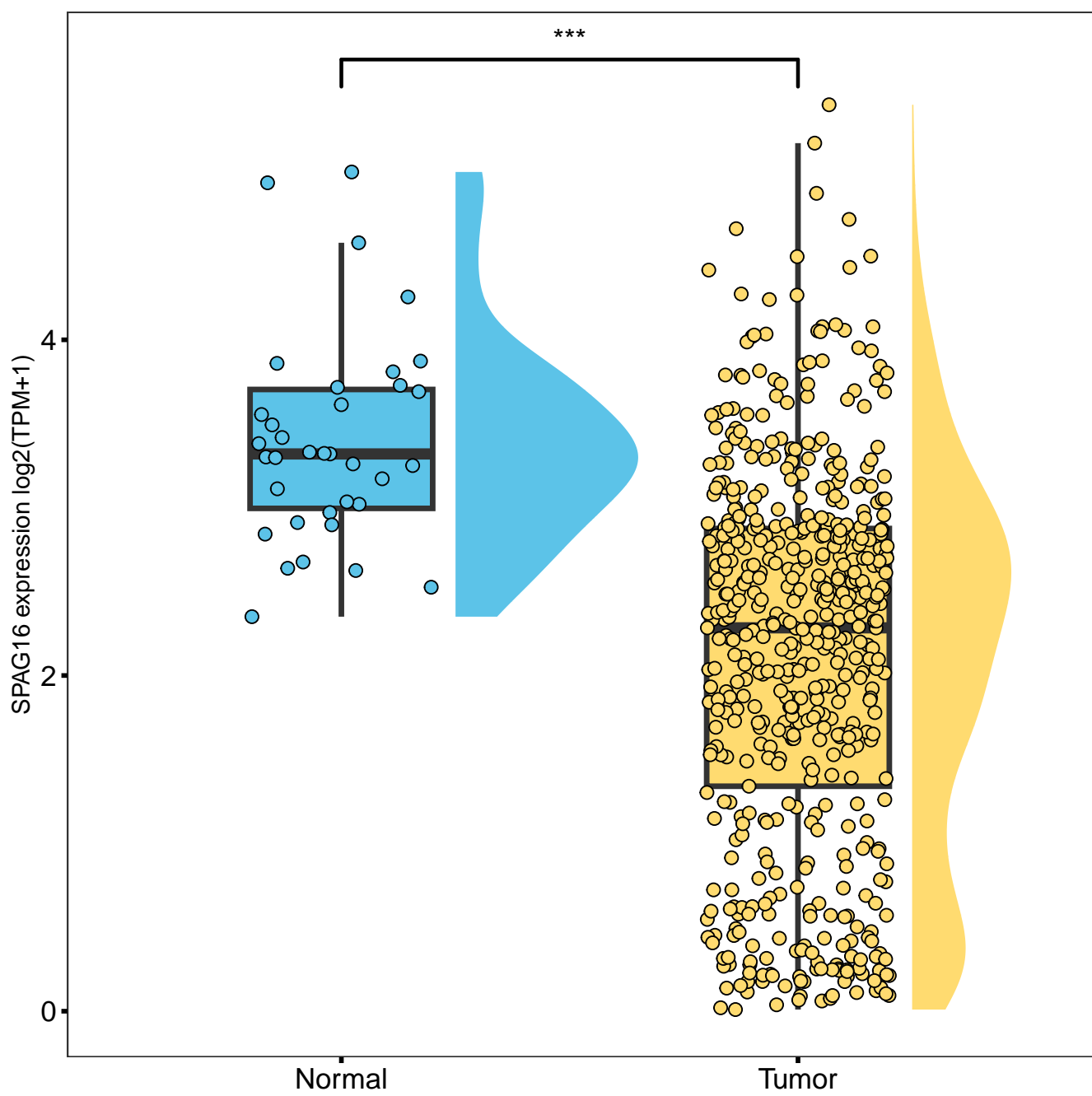

Supplement: Supplementary file 1 [file DataSheet1.zip › supplementary file/supplementary file 1/SPAG16_boxplot.pdf]

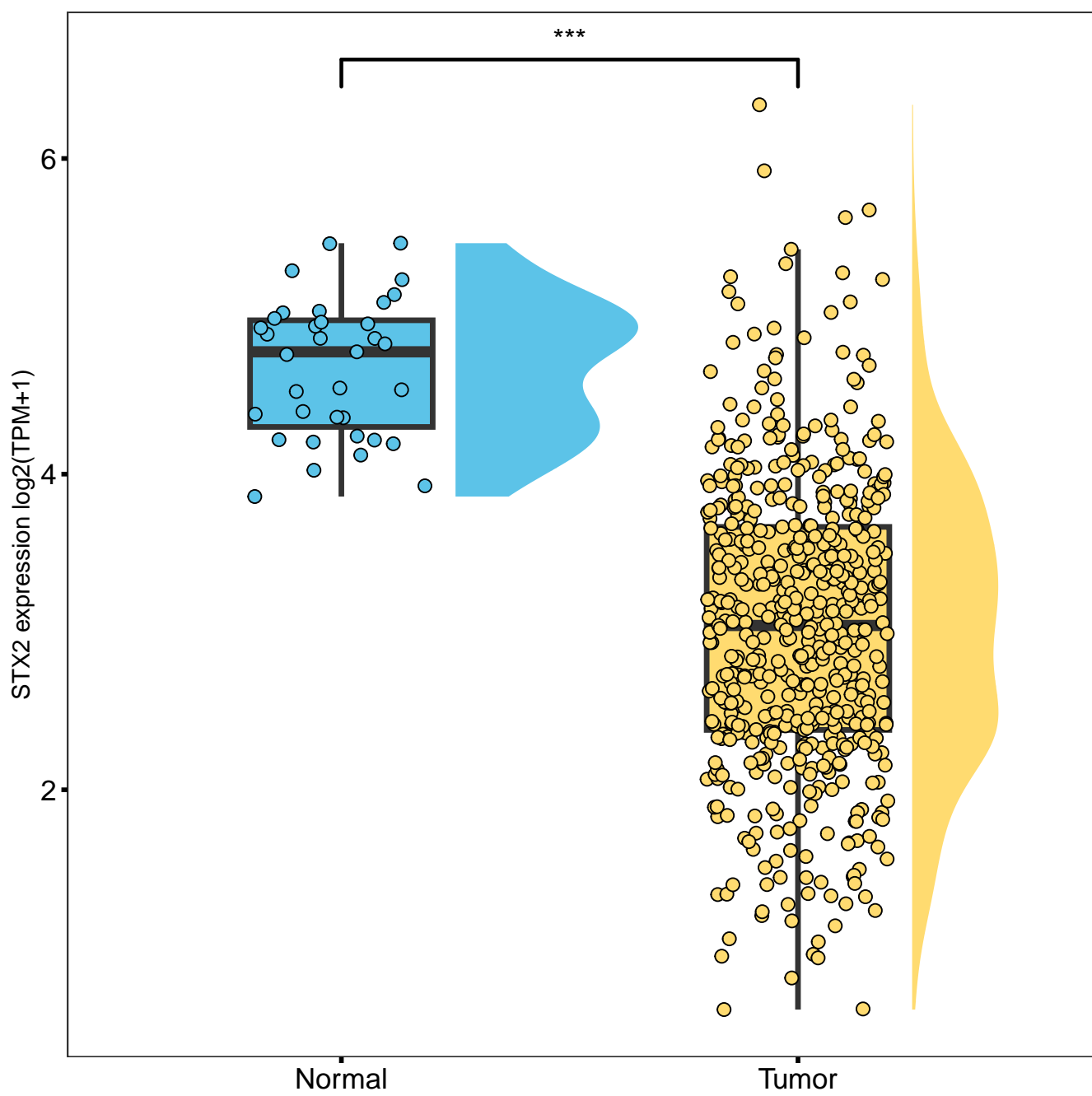

Supplement: Supplementary file 1 [file DataSheet1.zip › supplementary file/supplementary file 1/STX2_boxplot.pdf]

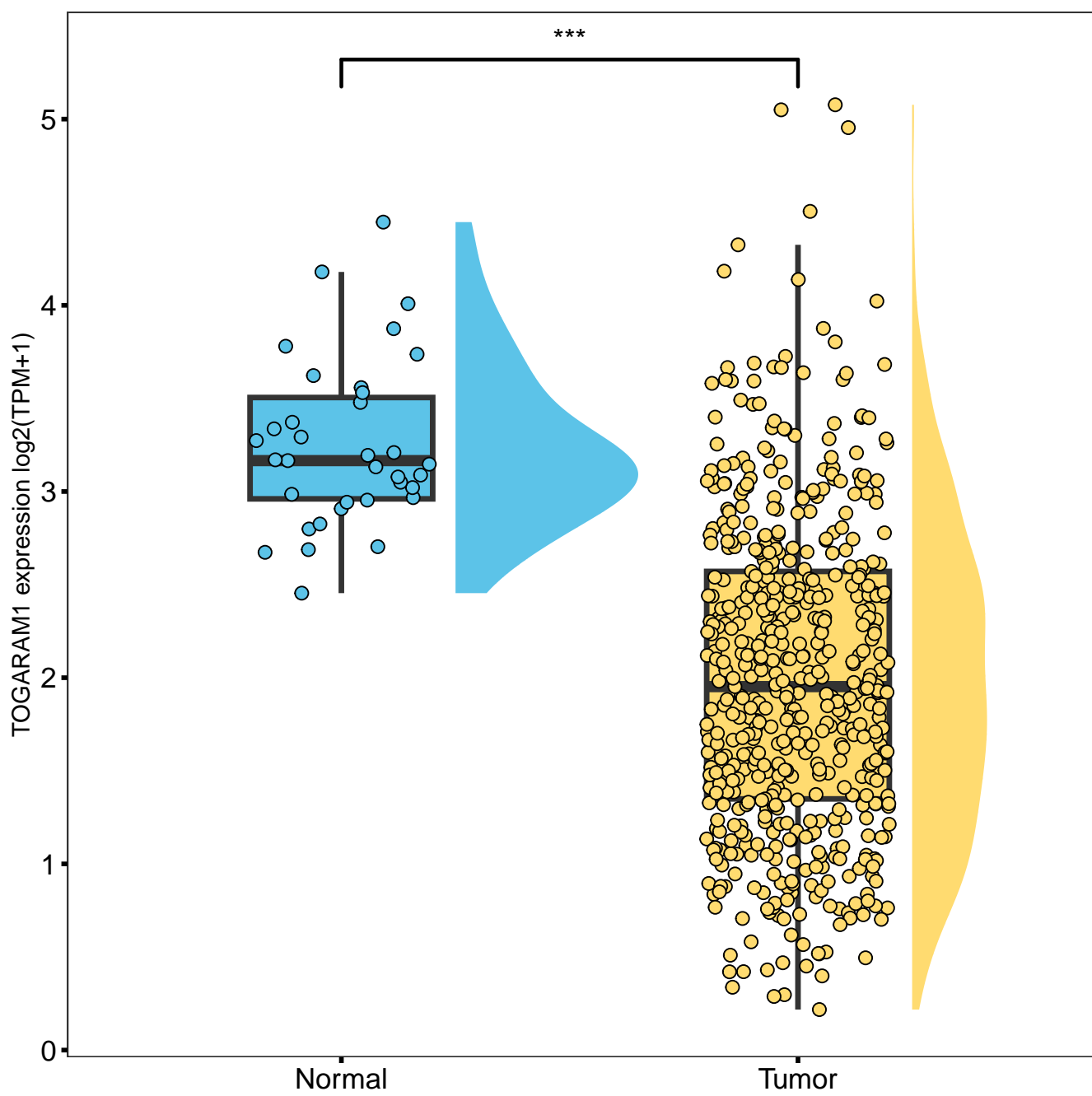

Supplement: Supplementary file 1 [file DataSheet1.zip › supplementary file/supplementary file 1/TOGARAM1_boxplot.pdf]

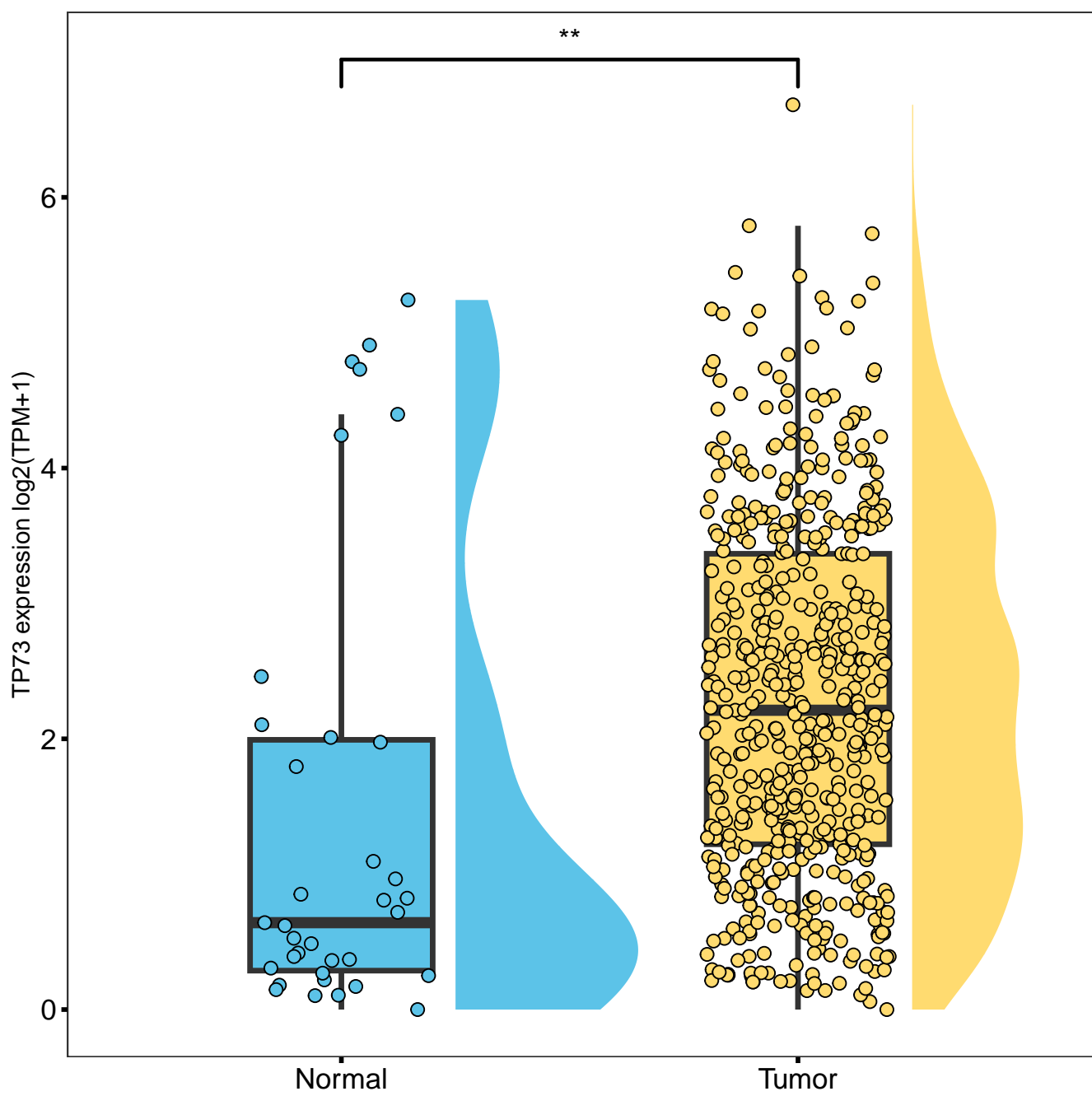

Supplement: Supplementary file 1 [file DataSheet1.zip › supplementary file/supplementary file 1/TP73_boxplot.pdf]

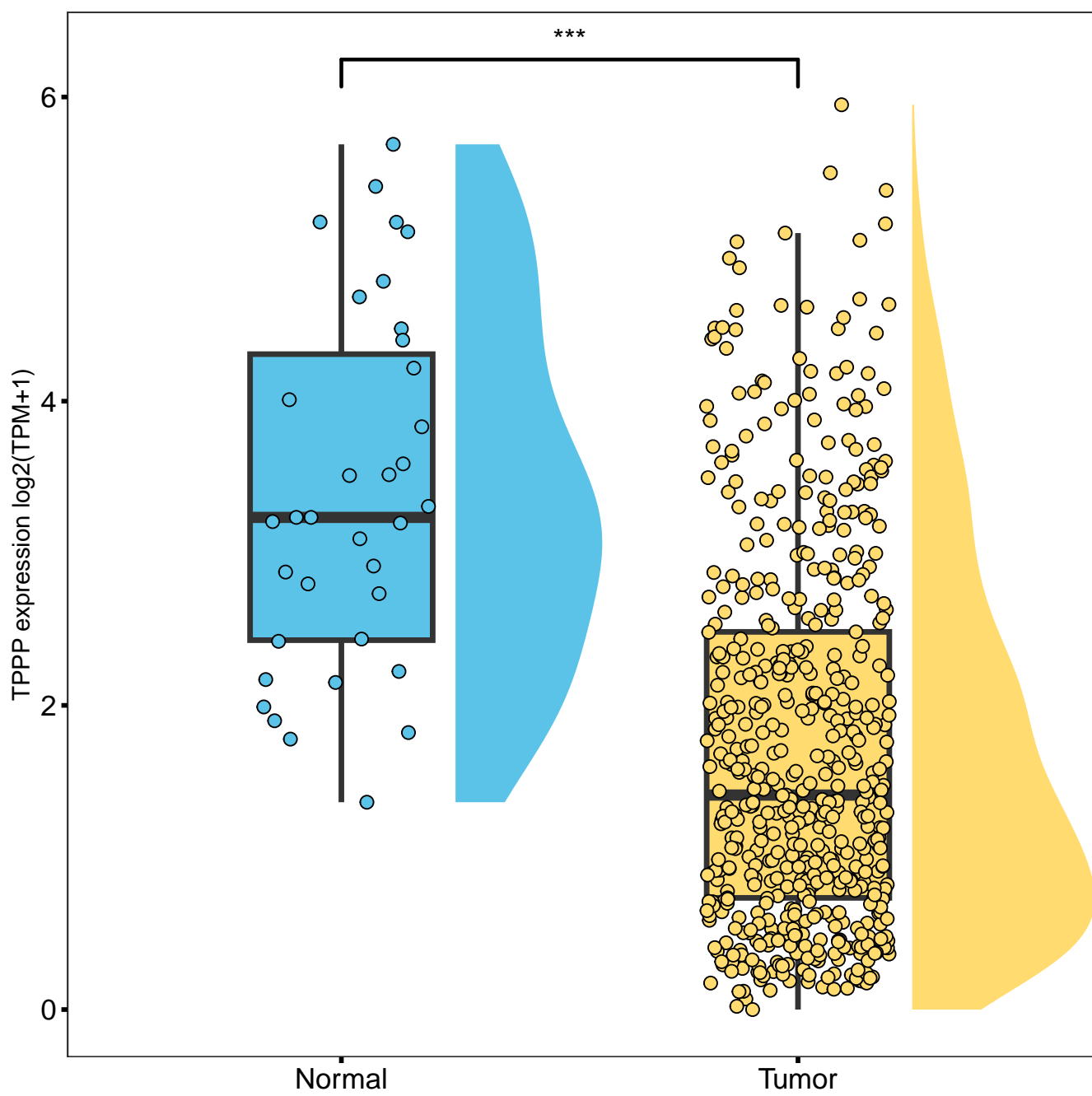

Supplement: Supplementary file 1 [file DataSheet1.zip › supplementary file/supplementary file 1/TPPP_boxplot.pdf]

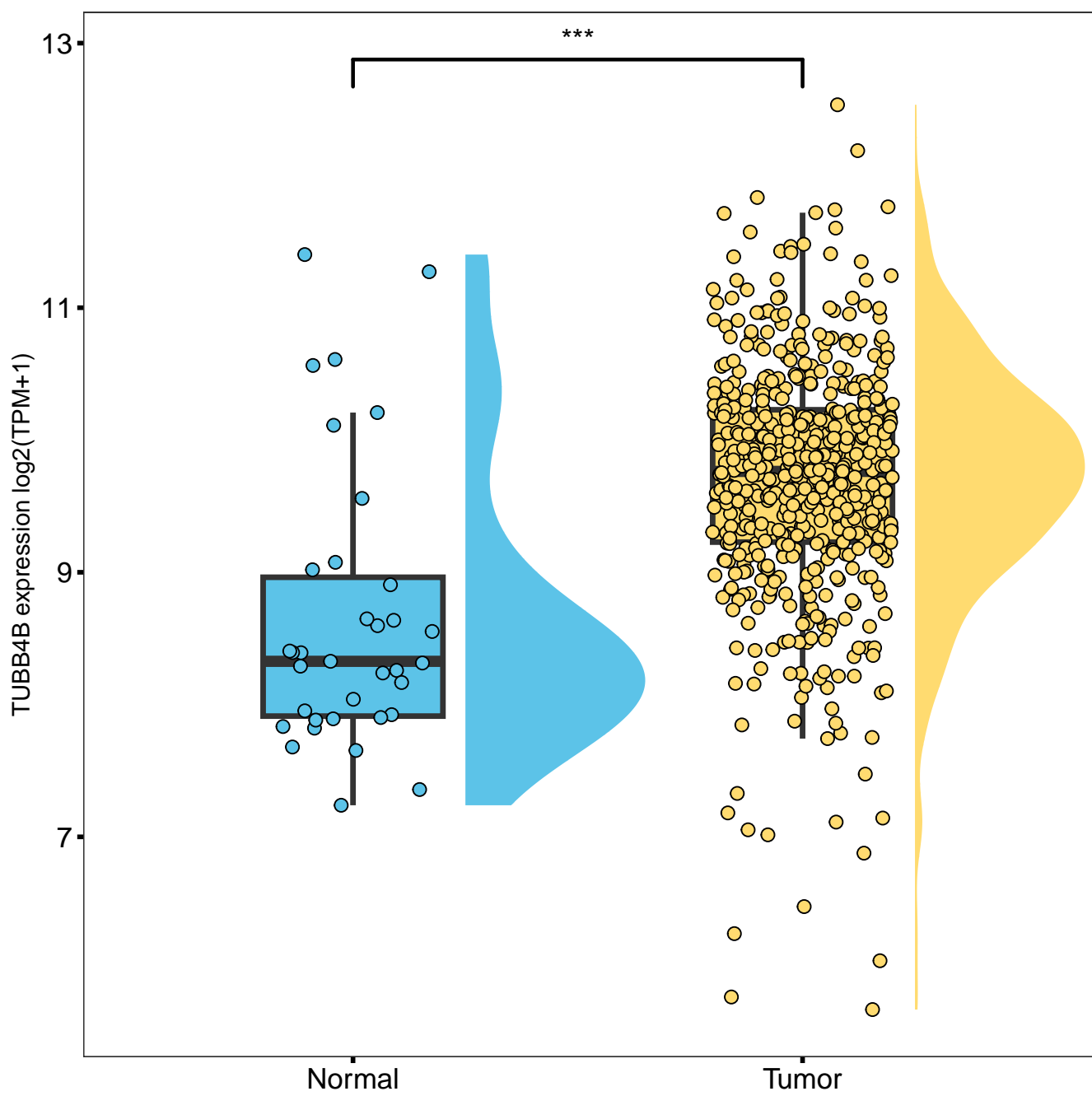

Supplement: Supplementary file 1 [file DataSheet1.zip › supplementary file/supplementary file 1/TUBB4B_boxplot.pdf]

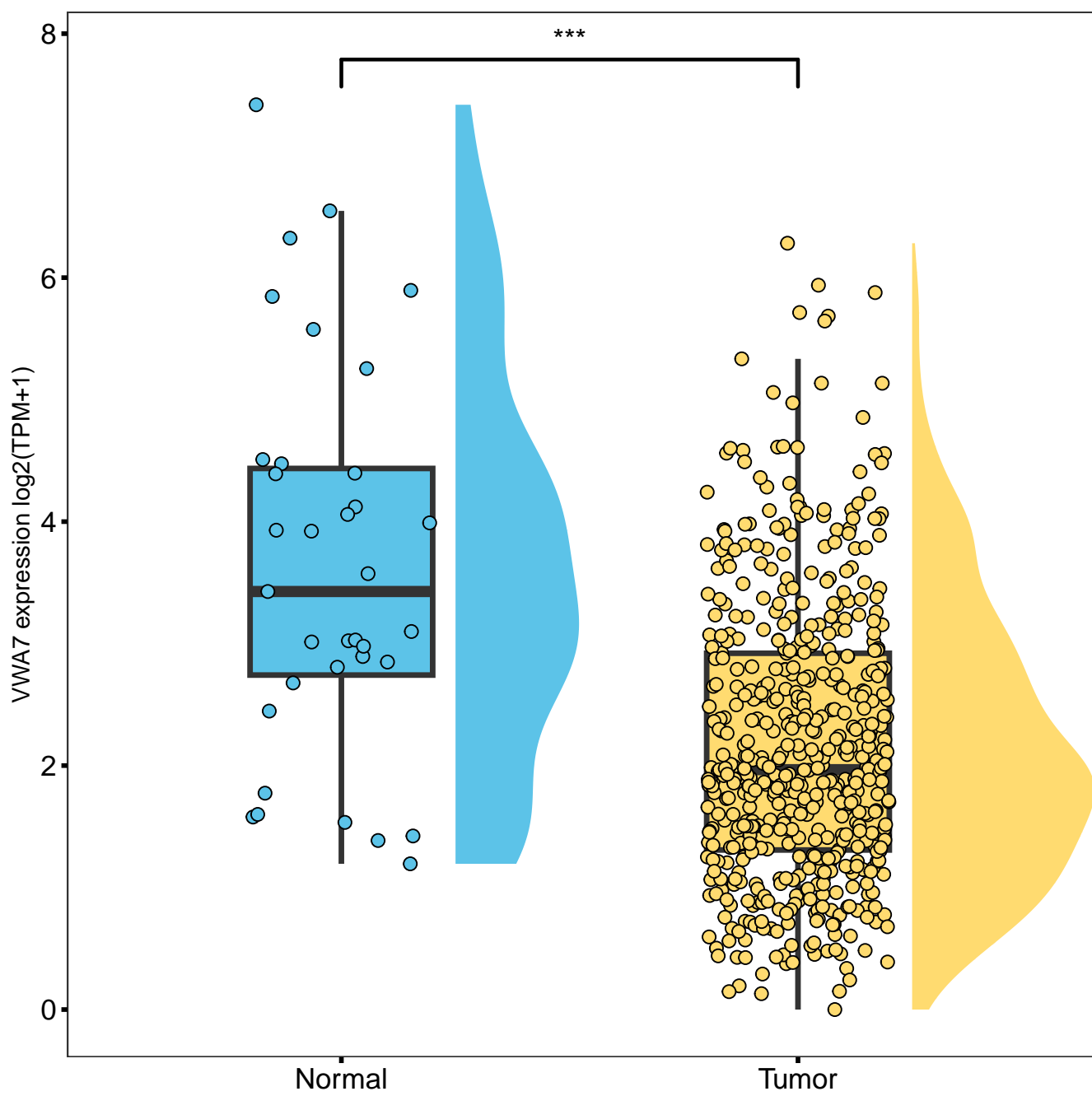

Supplement: Supplementary file 1 [file DataSheet1.zip › supplementary file/supplementary file 1/VWA7_boxplot.pdf]

CCDC138 High Low

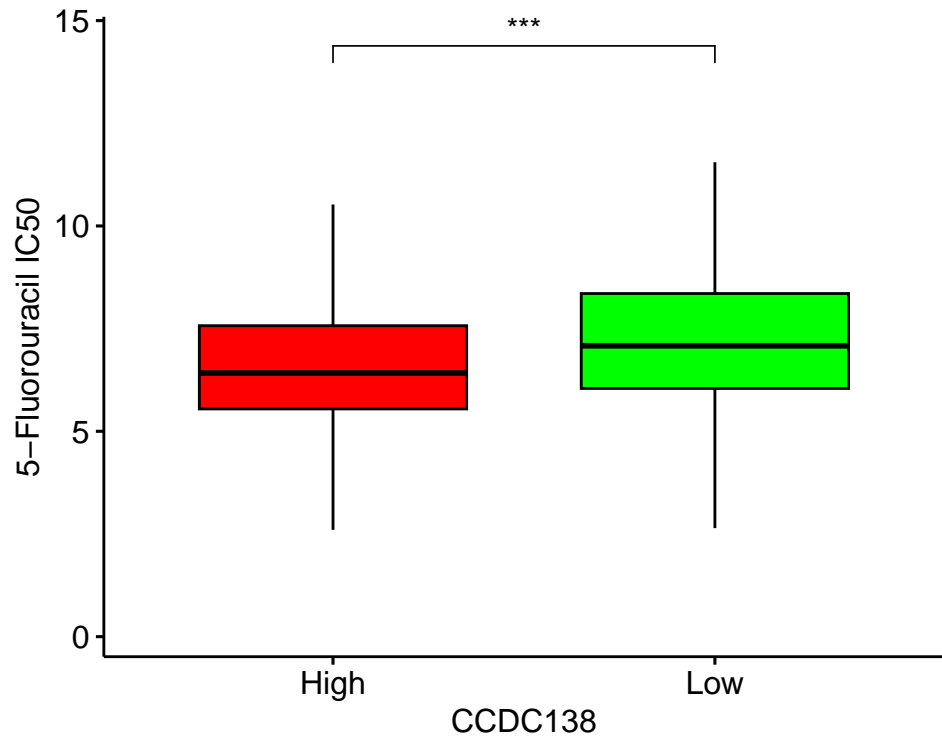

Supplement: Supplementary file 1 [file DataSheet1.zip › supplementary file/supplementary file 2/CCDC138_drugSenstivity.5-Fluorouracil.pdf]

CCDC138 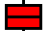 High 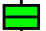 Low

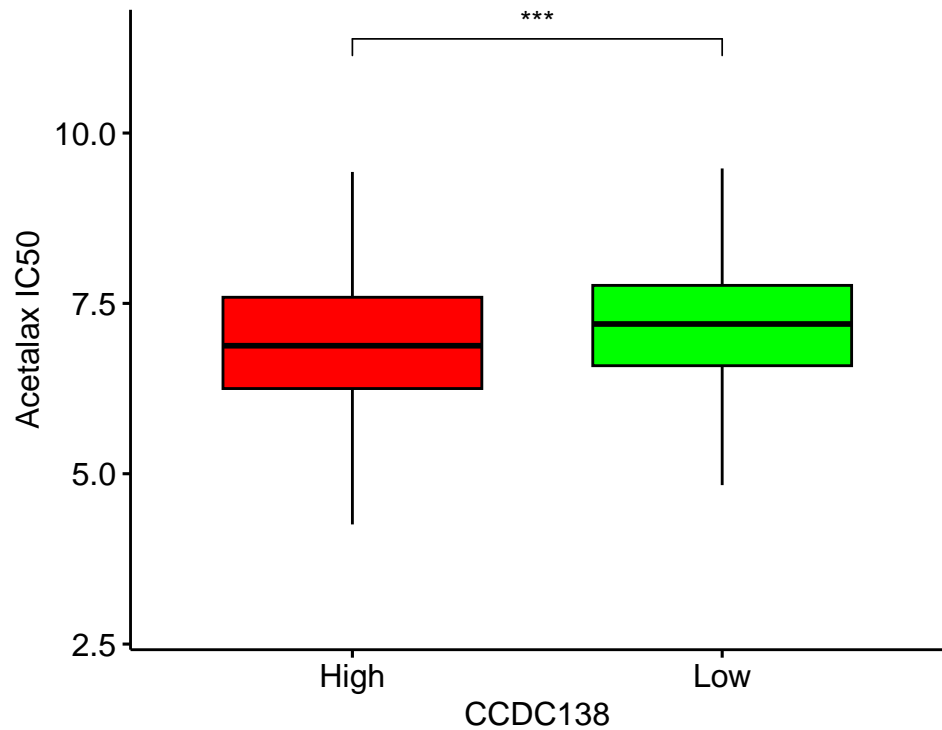

Supplement: Supplementary file 1 [file DataSheet1.zip › supplementary file/supplementary file 2/CCDC138_drugSenstivity.Acetalax.pdf]

CCDC138 High Low

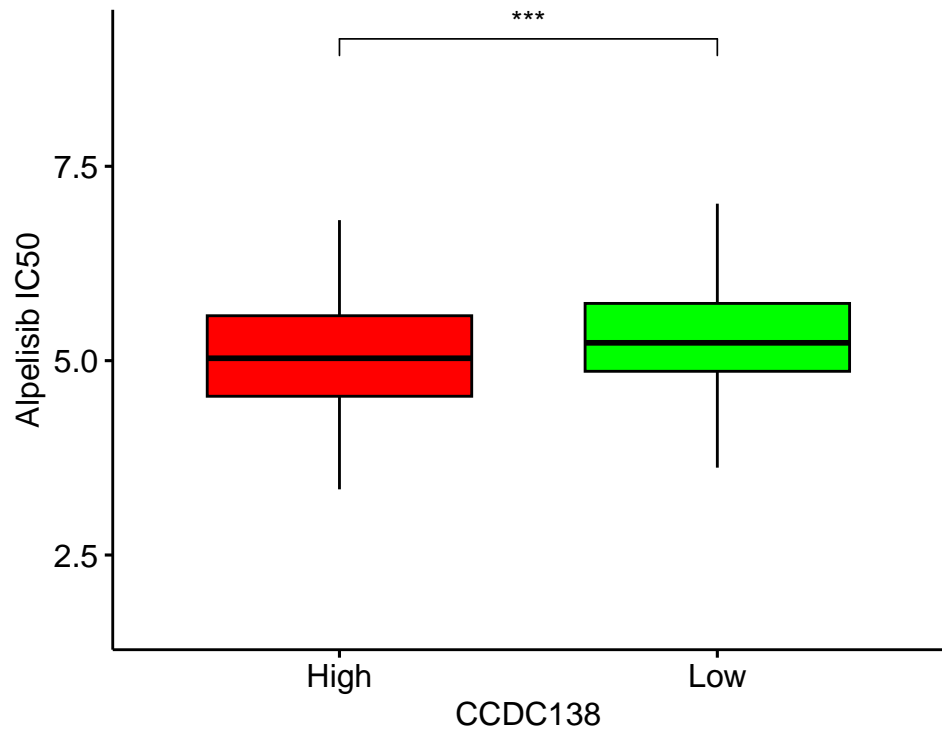

Supplement: Supplementary file 1 [file DataSheet1.zip › supplementary file/supplementary file 2/CCDC138_drugSenstivity.Alpelisib.pdf]

CCDC138 High Low

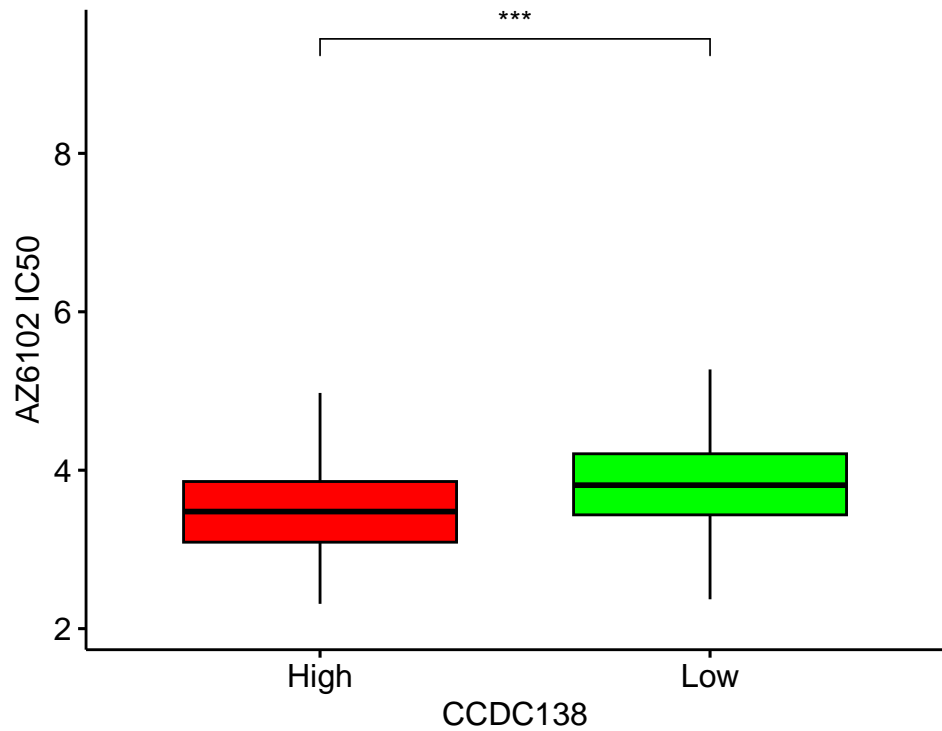

Supplement: Supplementary file 1 [file DataSheet1.zip › supplementary file/supplementary file 2/CCDC138_drugSenstivity.AZ6102.pdf]

CCDC138 High Low

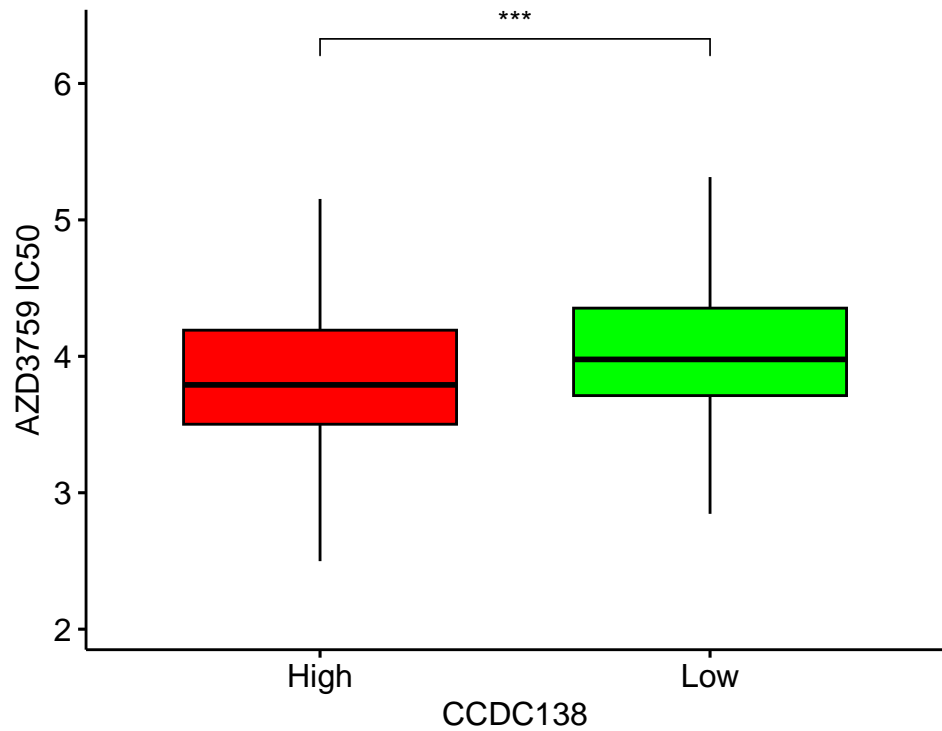

Supplement: Supplementary file 1 [file DataSheet1.zip › supplementary file/supplementary file 2/CCDC138_drugSenstivity.AZD3759.pdf]

CCDC138 High Low

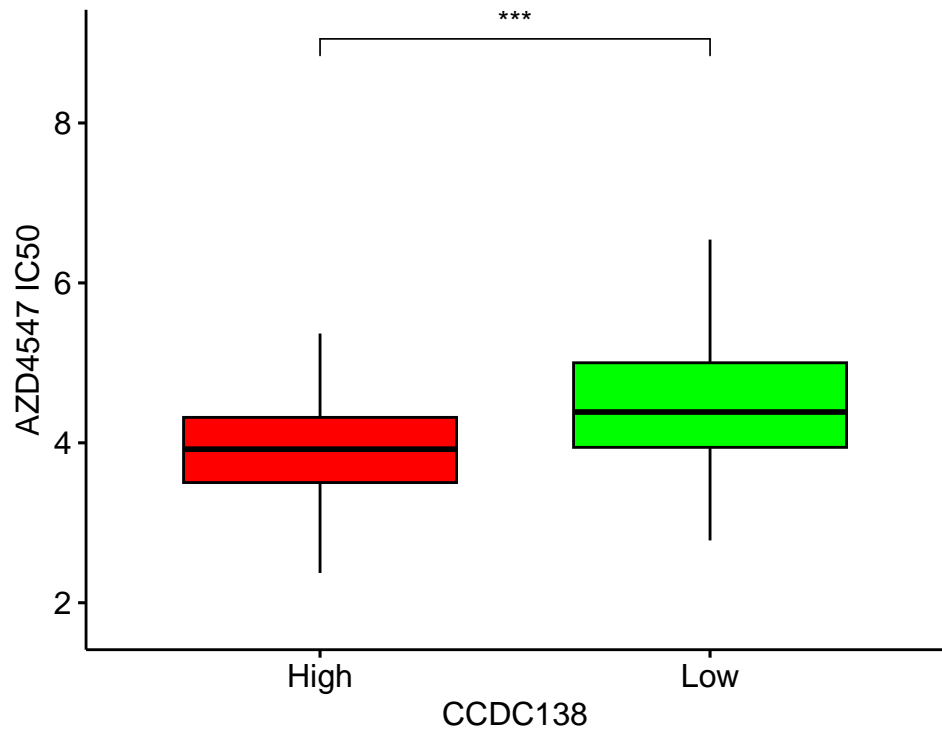

Supplement: Supplementary file 1 [file DataSheet1.zip › supplementary file/supplementary file 2/CCDC138_drugSenstivity.AZD4547.pdf]

CCDC138 High Low

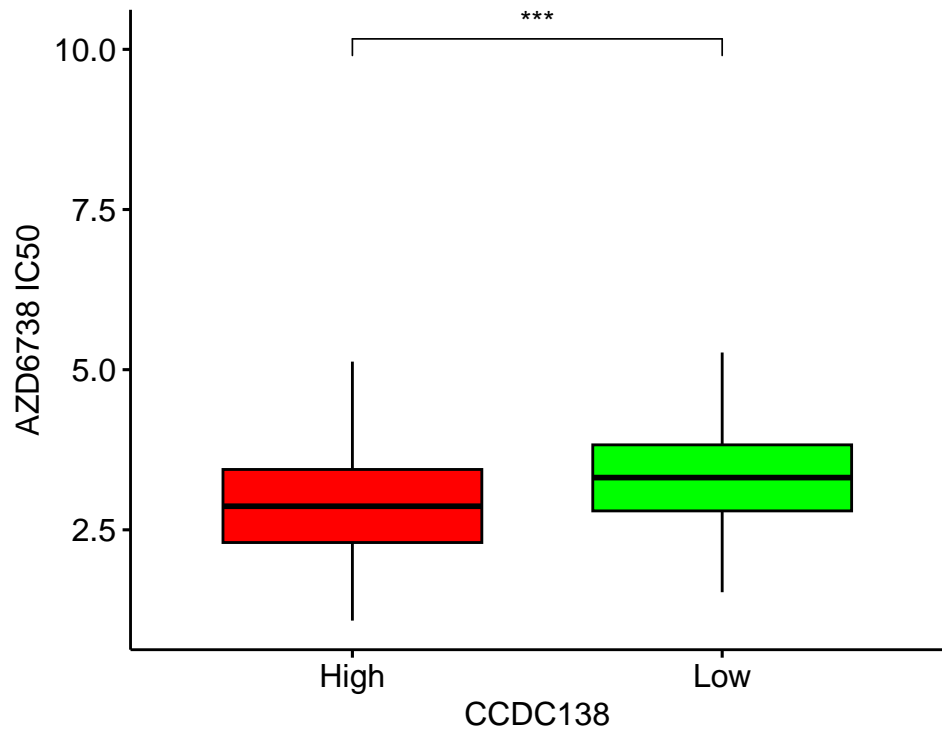

Supplement: Supplementary file 1 [file DataSheet1.zip › supplementary file/supplementary file 2/CCDC138_drugSenstivity.AZD6738.pdf]

CCDC138 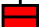 High 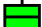 Low

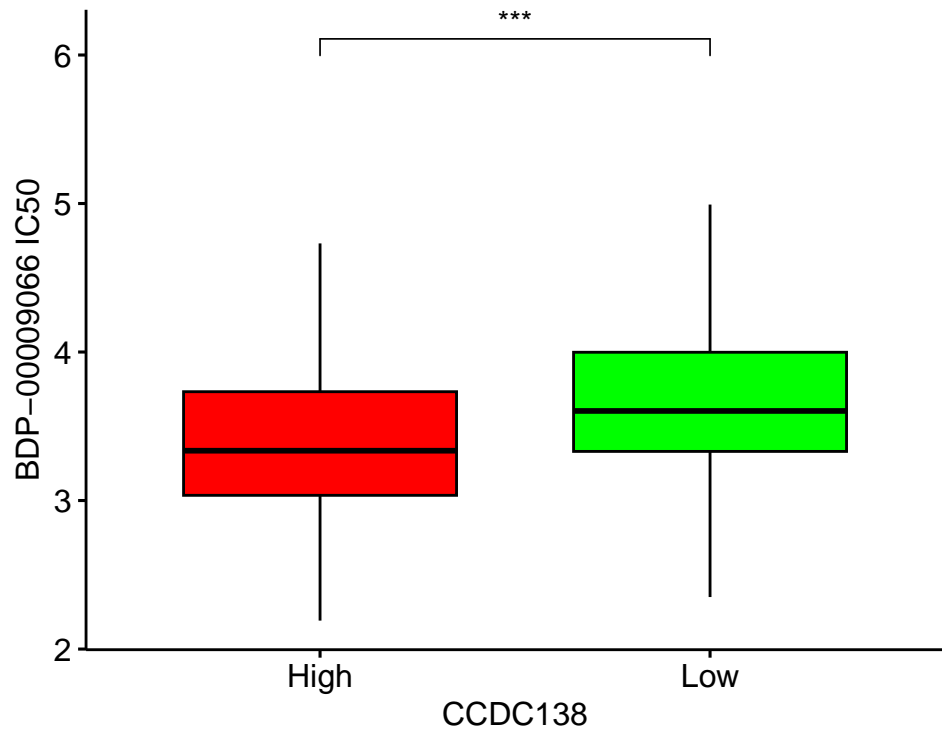

Supplement: Supplementary file 1 [file DataSheet1.zip › supplementary file/supplementary file 2/CCDC138_drugSenstivity.BDP-00009066.pdf]

CCDC138 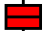 High 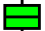 Low

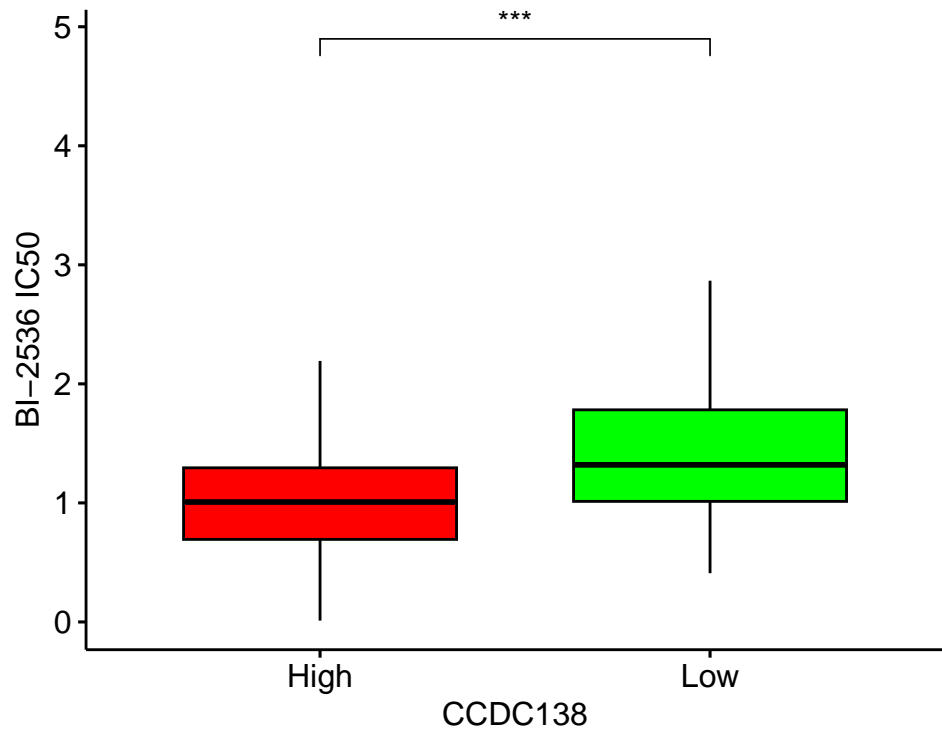

Supplement: Supplementary file 1 [file DataSheet1.zip › supplementary file/supplementary file 2/CCDC138_drugSenstivity.BI-2536.pdf]

CCDC138 High Low

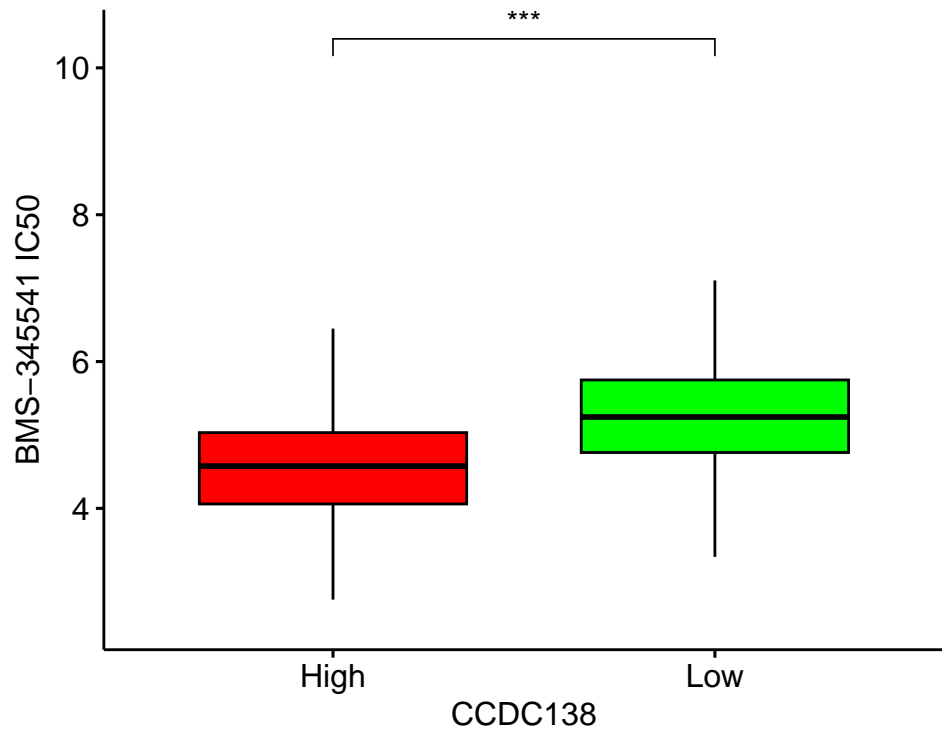

Supplement: Supplementary file 1 [file DataSheet1.zip › supplementary file/supplementary file 2/CCDC138_drugSenstivity.BMS-345541.pdf]

CCDC138 High Low

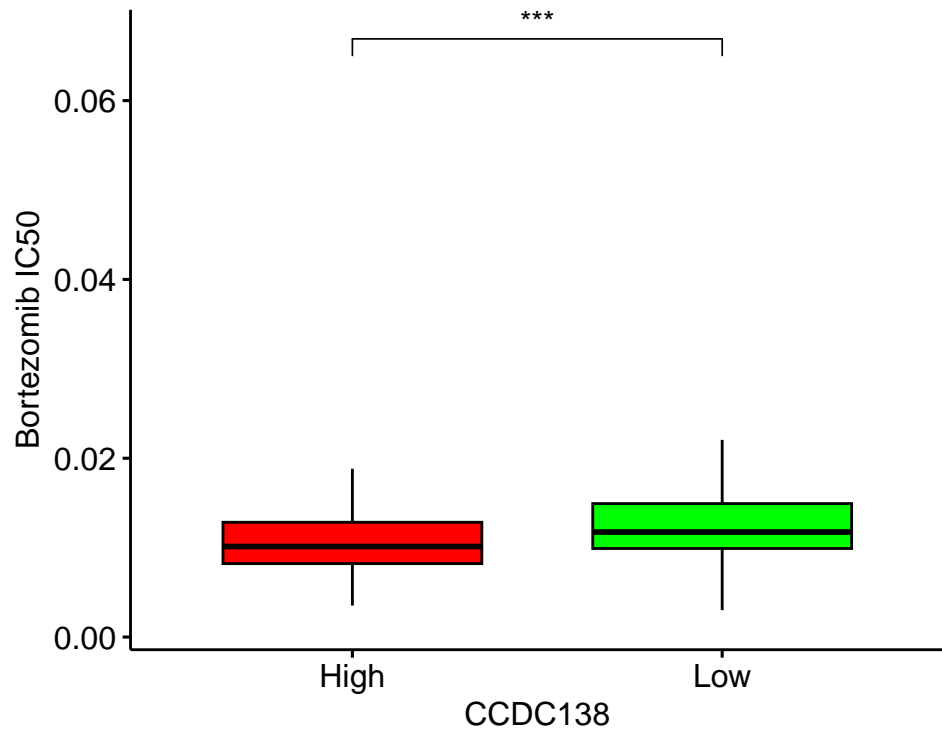

Supplement: Supplementary file 1 [file DataSheet1.zip › supplementary file/supplementary file 2/CCDC138_drugSenstivity.Bortezomib.pdf]

CCDC138 High Low

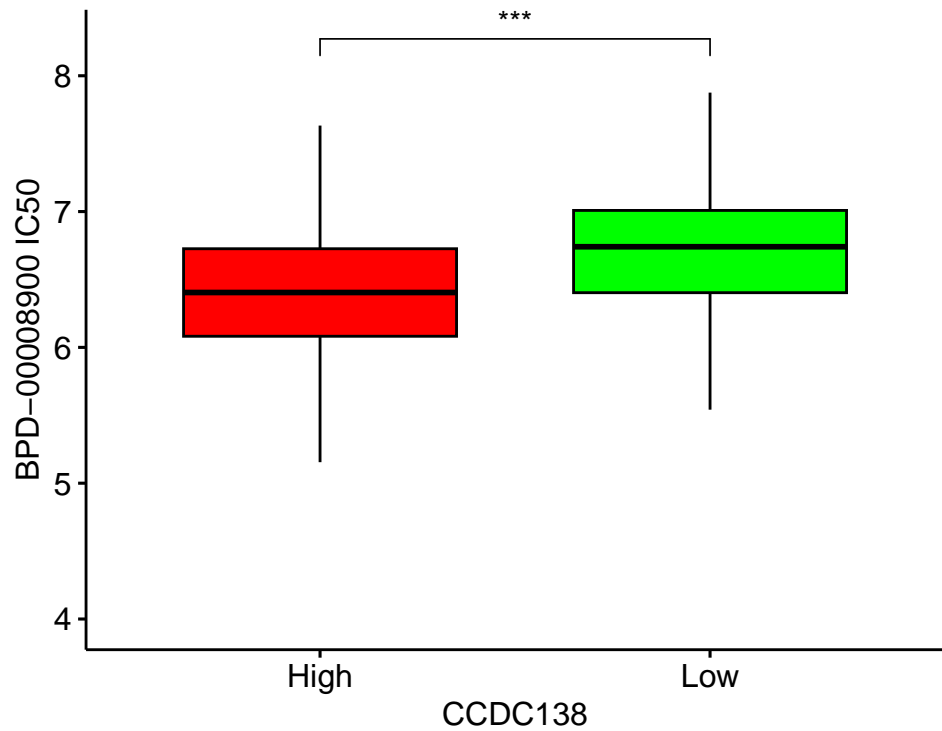

Supplement: Supplementary file 1 [file DataSheet1.zip › supplementary file/supplementary file 2/CCDC138_drugSenstivity.BPD-00008900.pdf]

CCDC138 High Low

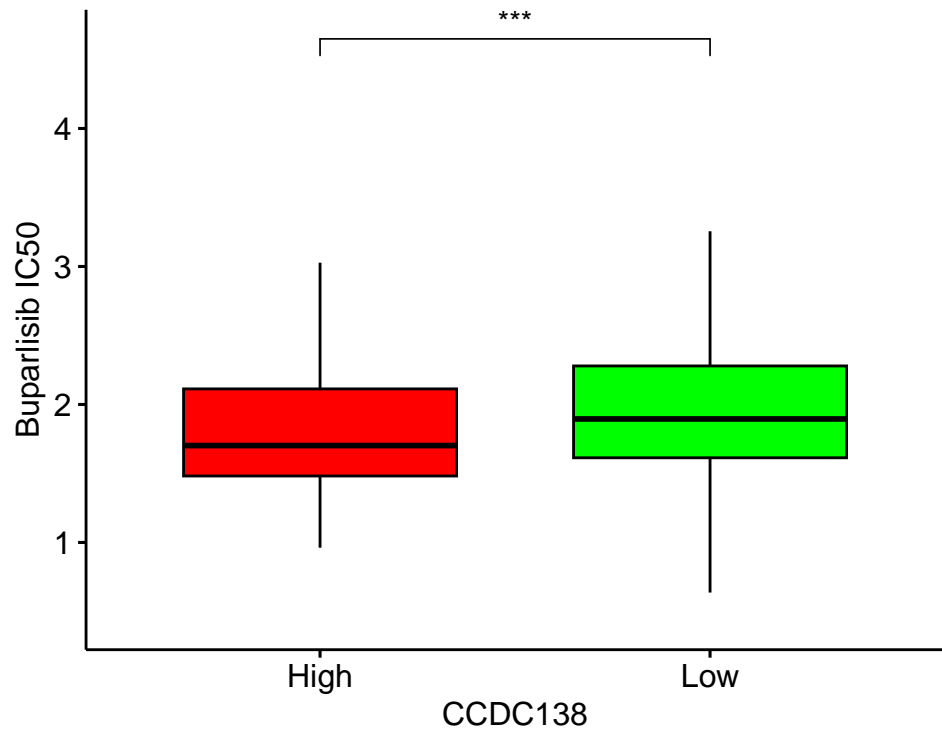

Supplement: Supplementary file 1 [file DataSheet1.zip › supplementary file/supplementary file 2/CCDC138_drugSenstivity.Buparlisib.pdf]

CCDC138 High Low

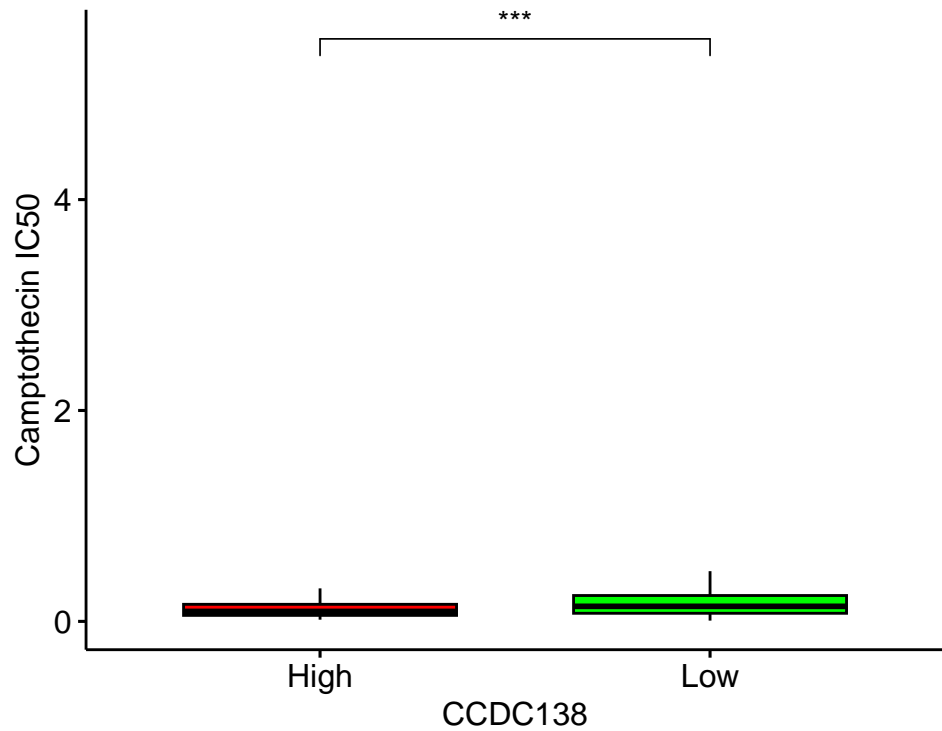

Supplement: Supplementary file 1 [file DataSheet1.zip › supplementary file/supplementary file 2/CCDC138_drugSenstivity.Camptothecin.pdf]

CCDC138 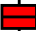 High 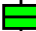 Low

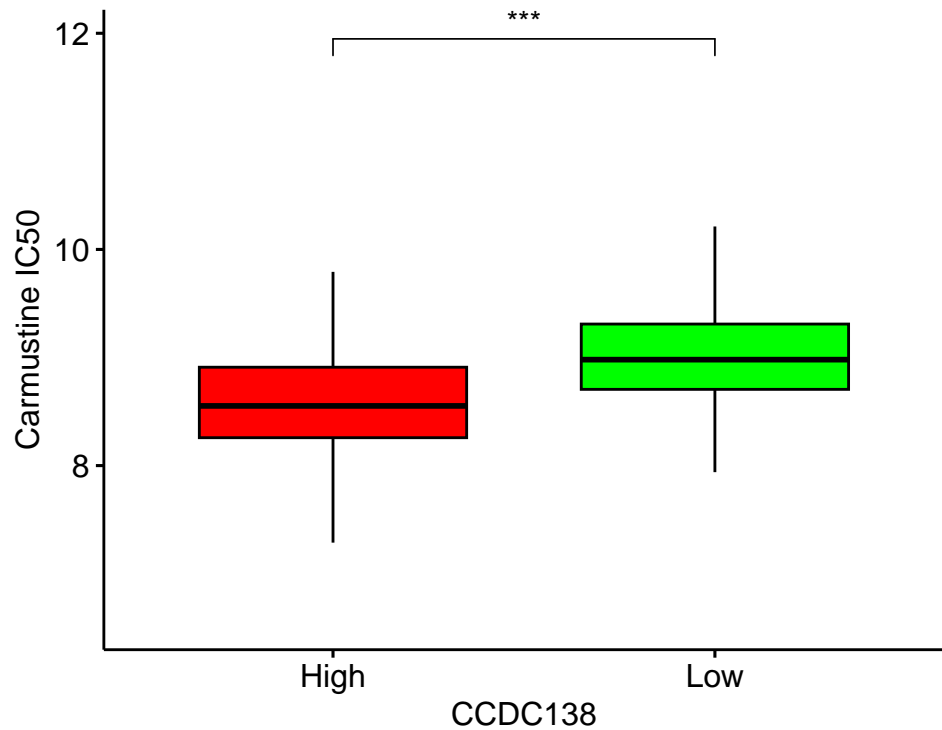

Supplement: Supplementary file 1 [file DataSheet1.zip › supplementary file/supplementary file 2/CCDC138_drugSenstivity.Carmustine.pdf]

CCDC138 High Low

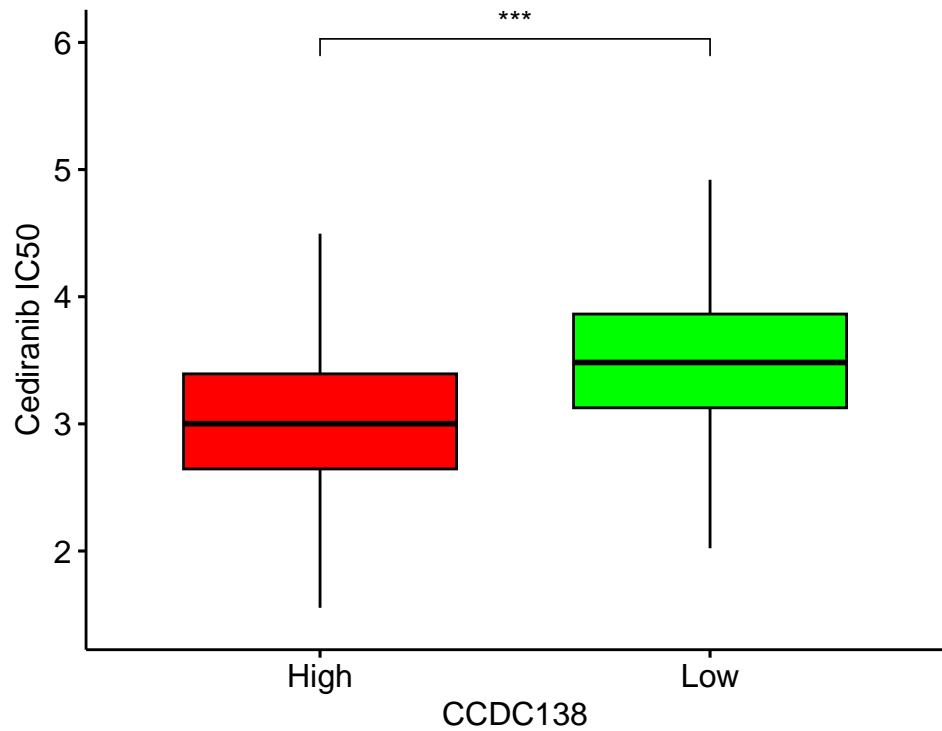

Supplement: Supplementary file 1 [file DataSheet1.zip › supplementary file/supplementary file 2/CCDC138_drugSenstivity.Cediranib.pdf]

CCDC138 High Low

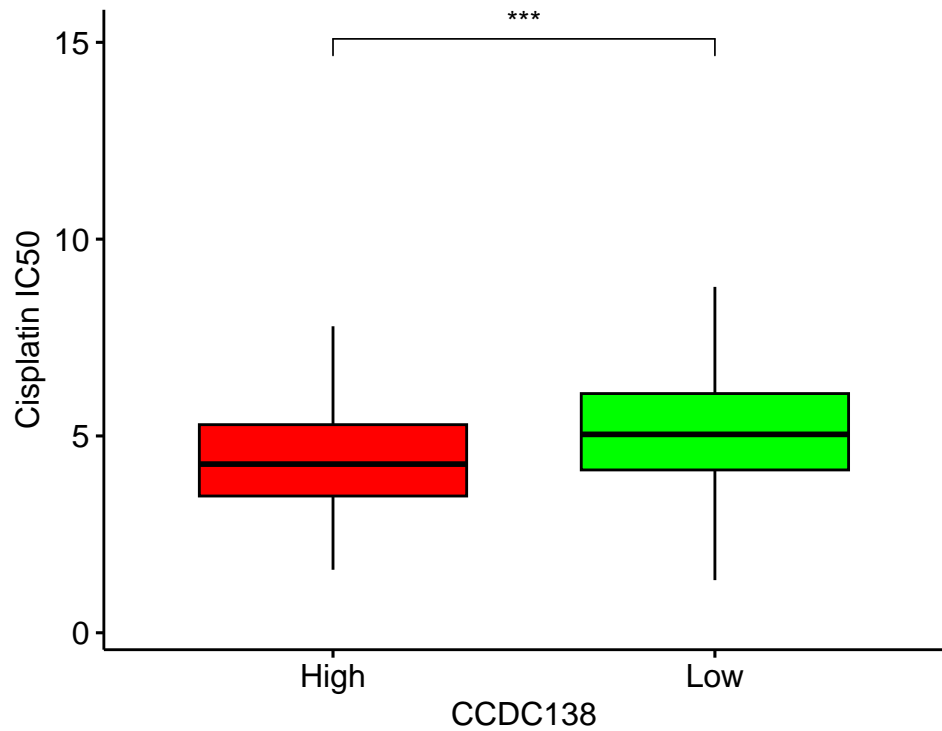

Supplement: Supplementary file 1 [file DataSheet1.zip › supplementary file/supplementary file 2/CCDC138_drugSenstivity.Cisplatin.pdf]

CCDC138 High Low

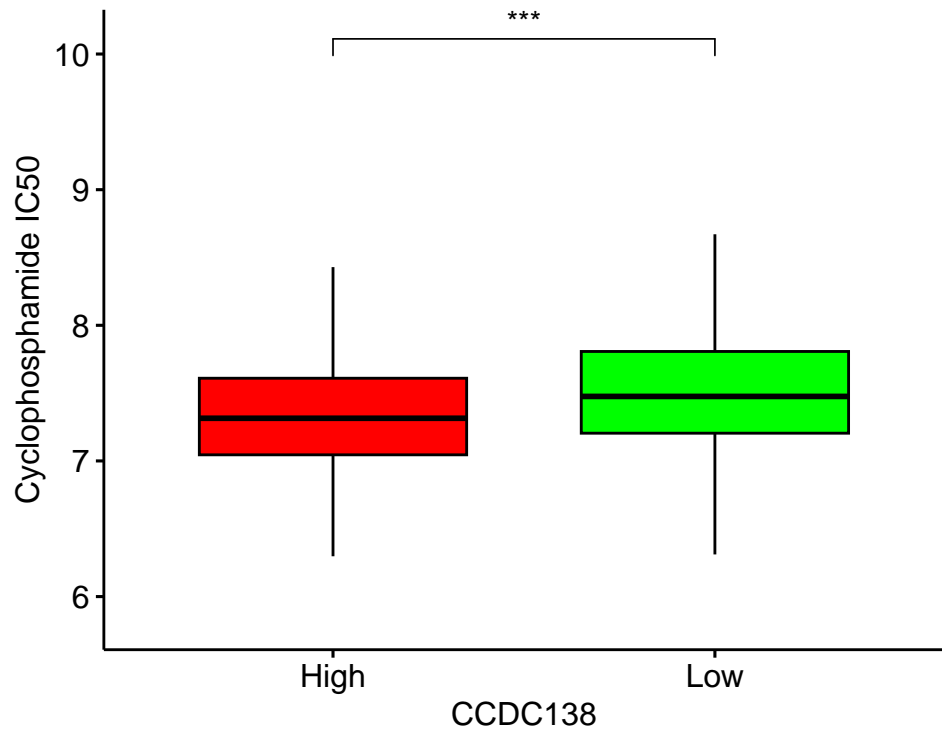

Supplement: Supplementary file 1 [file DataSheet1.zip › supplementary file/supplementary file 2/CCDC138_drugSenstivity.Cyclophosphamide.pdf]

CCDC138 High Low

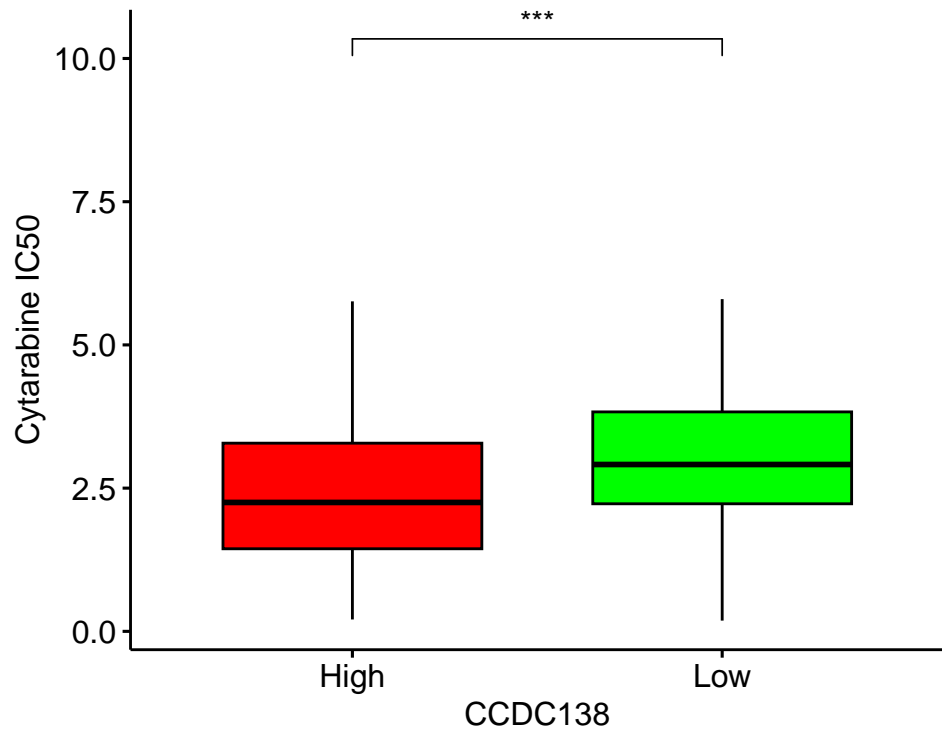

Supplement: Supplementary file 1 [file DataSheet1.zip › supplementary file/supplementary file 2/CCDC138_drugSenstivity.Cytarabine.pdf]

CCDC138 High Low

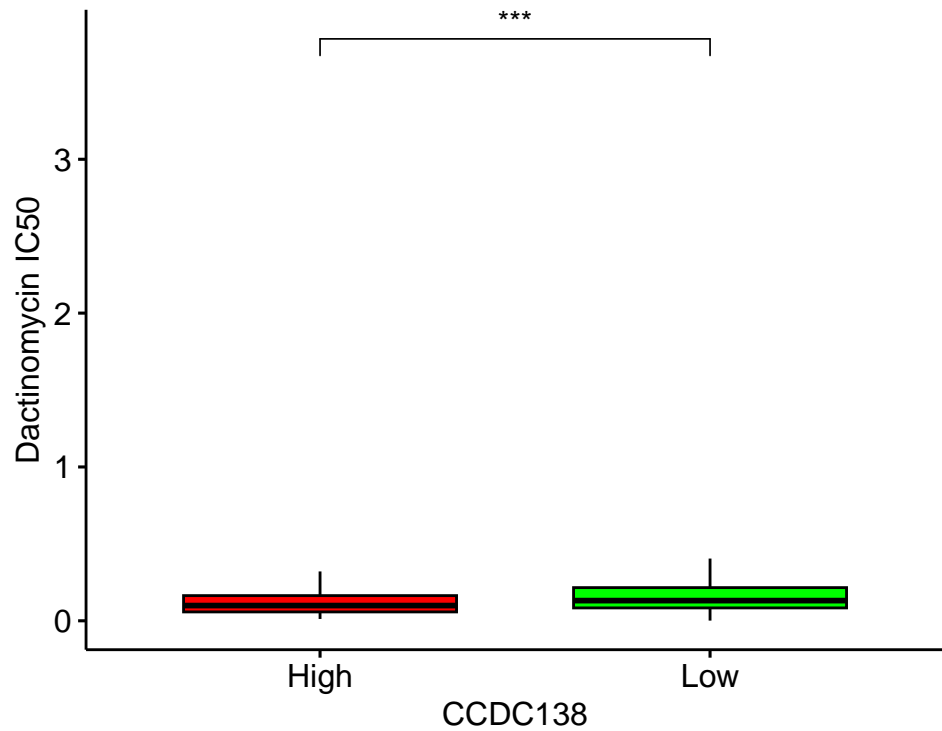

Supplement: Supplementary file 1 [file DataSheet1.zip › supplementary file/supplementary file 2/CCDC138_drugSenstivity.Dactinomycin.pdf]

CCDC138 High Low

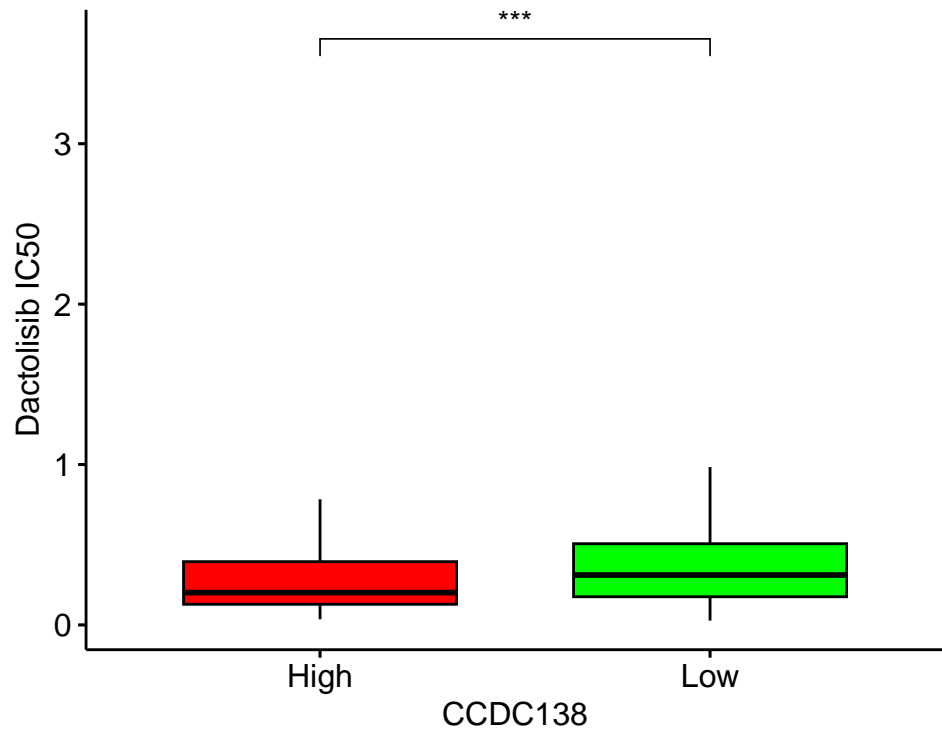

Supplement: Supplementary file 1 [file DataSheet1.zip › supplementary file/supplementary file 2/CCDC138_drugSenstivity.Dactolisib.pdf]

CCDC138 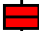 High 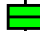 Low

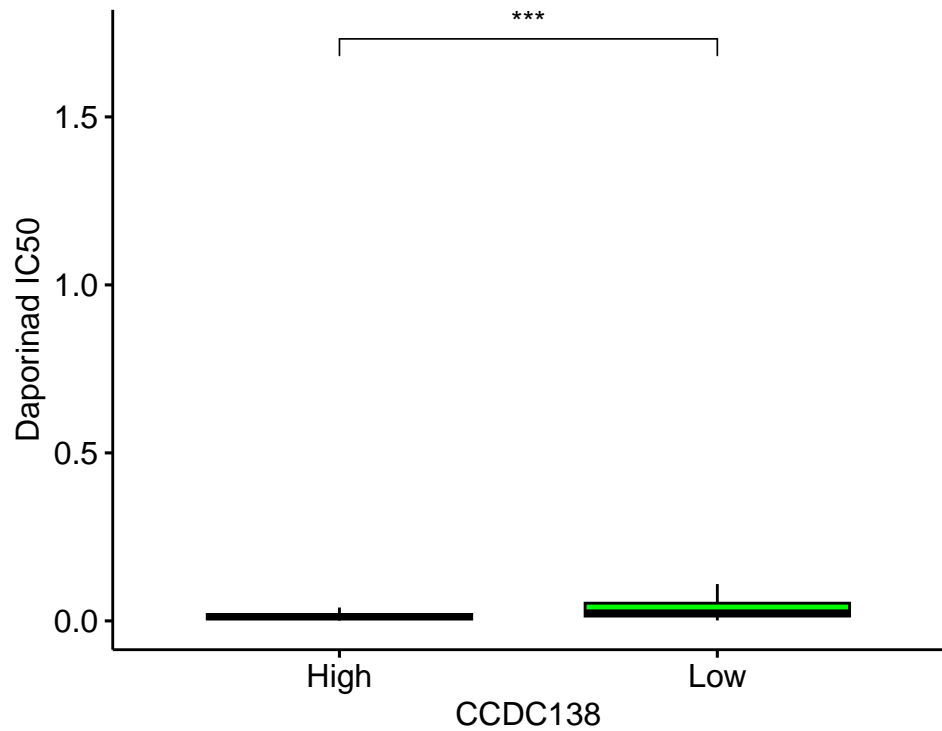

Supplement: Supplementary file 1 [file DataSheet1.zip › supplementary file/supplementary file 2/CCDC138_drugSenstivity.Daporinad.pdf]

CCDC138 High Low

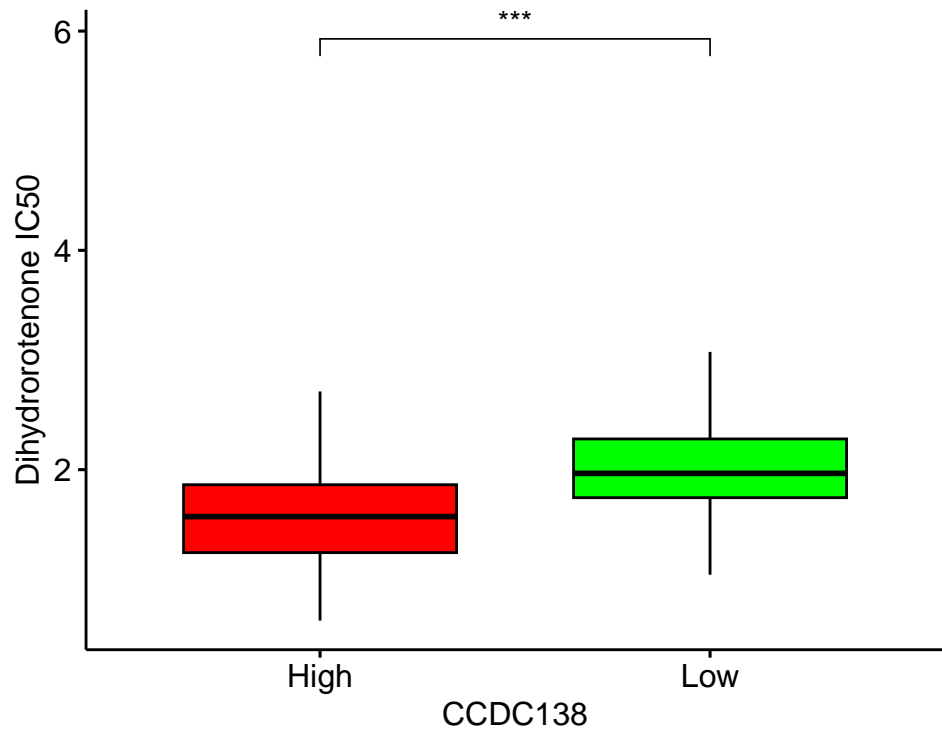

Supplement: Supplementary file 1 [file DataSheet1.zip › supplementary file/supplementary file 2/CCDC138_drugSenstivity.Dihydrorotenone.pdf]

CCDC138 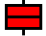 High 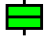 Low

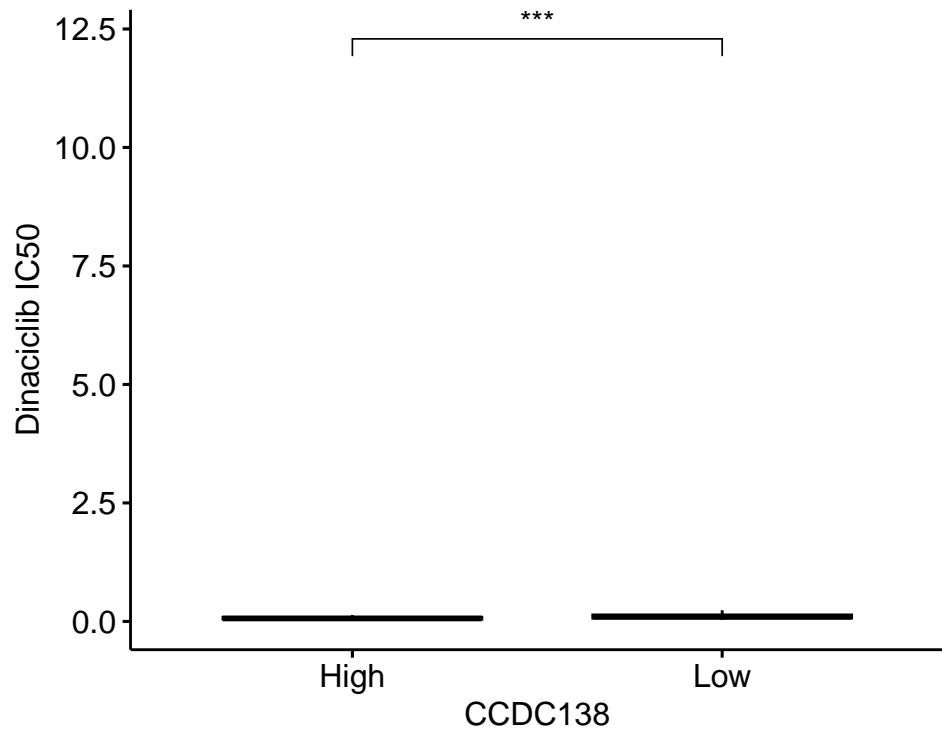

Supplement: Supplementary file 1 [file DataSheet1.zip › supplementary file/supplementary file 2/CCDC138_drugSenstivity.Dinaciclib.pdf]

CCDC138 High Low

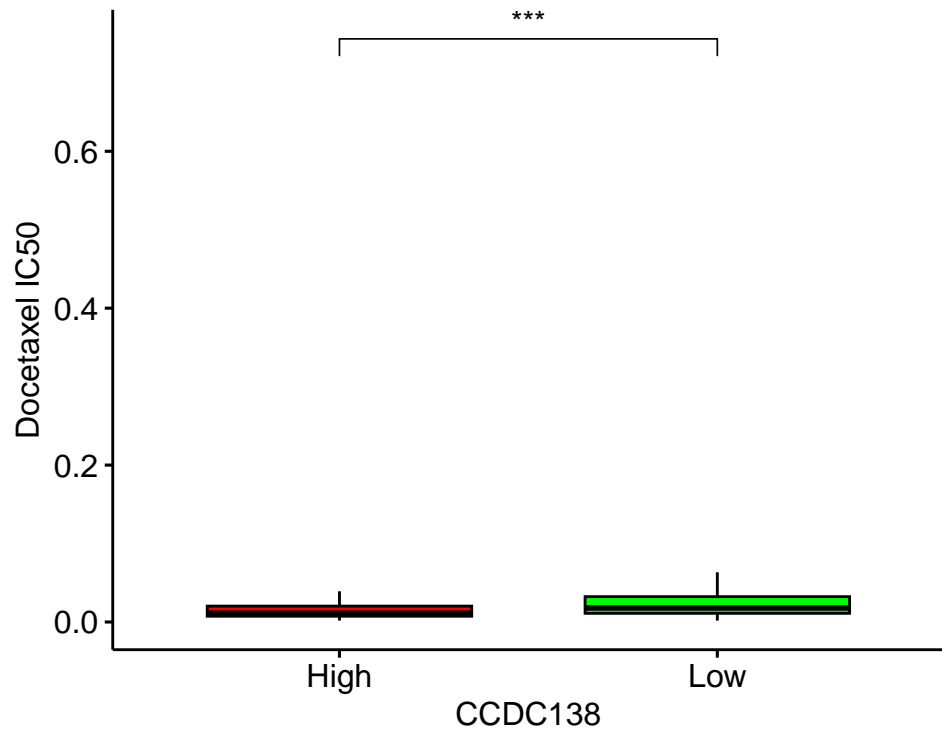

Supplement: Supplementary file 1 [file DataSheet1.zip › supplementary file/supplementary file 2/CCDC138_drugSenstivity.Docetaxel.pdf]

CCDC138 High Low

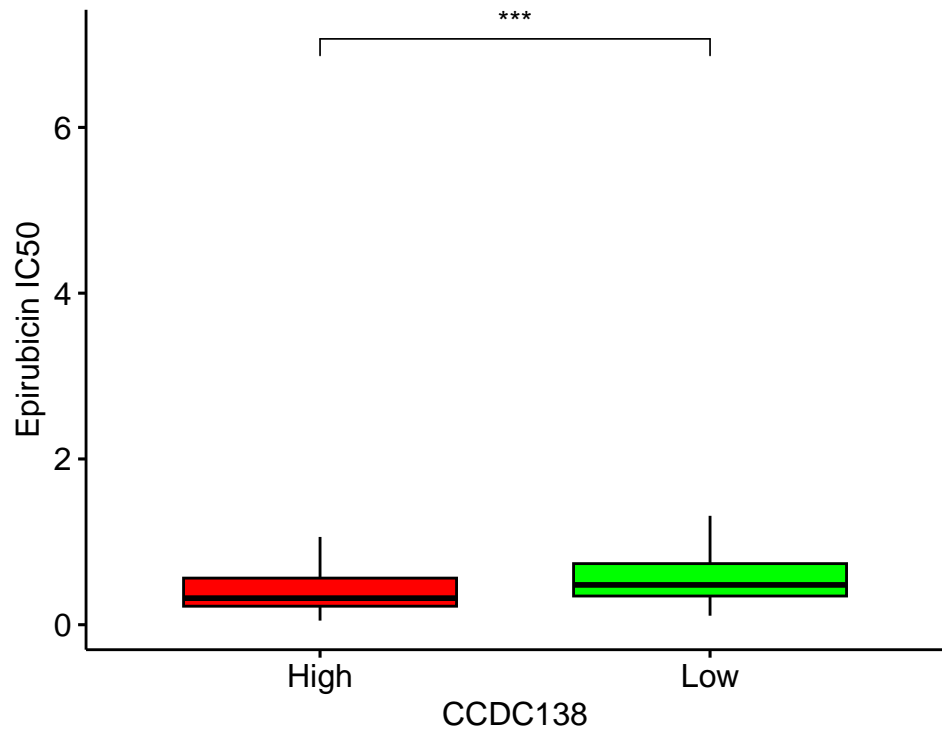

Supplement: Supplementary file 1 [file DataSheet1.zip › supplementary file/supplementary file 2/CCDC138_drugSenstivity.Epirubicin.pdf]

CCDC138 High Low

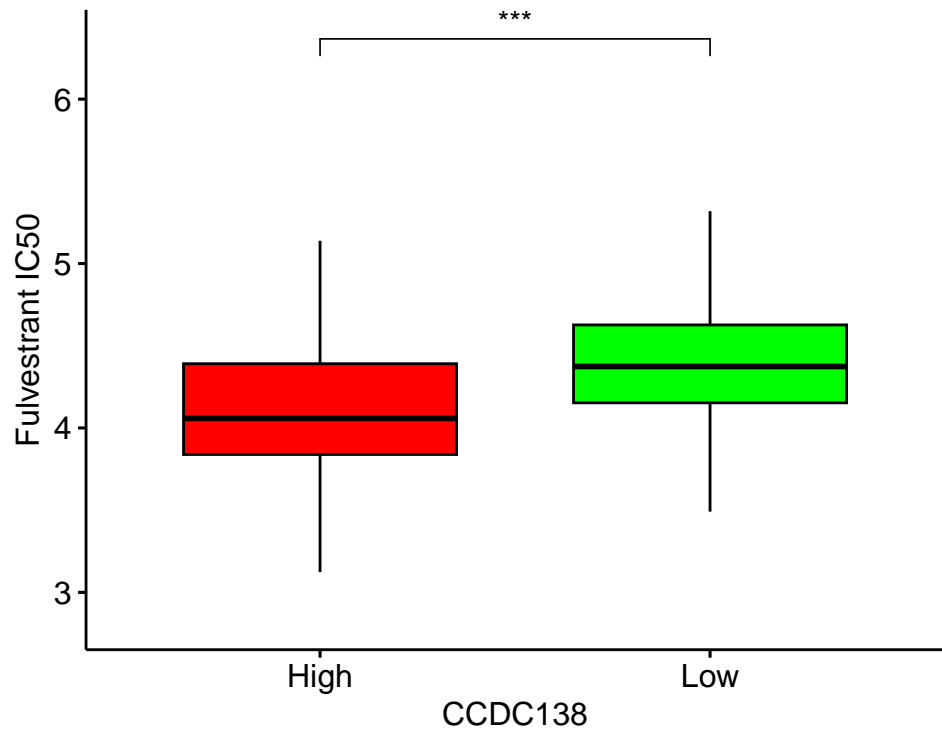

Supplement: Supplementary file 1 [file DataSheet1.zip › supplementary file/supplementary file 2/CCDC138_drugSenstivity.Fulvestrant.pdf]

CCDC138 High Low

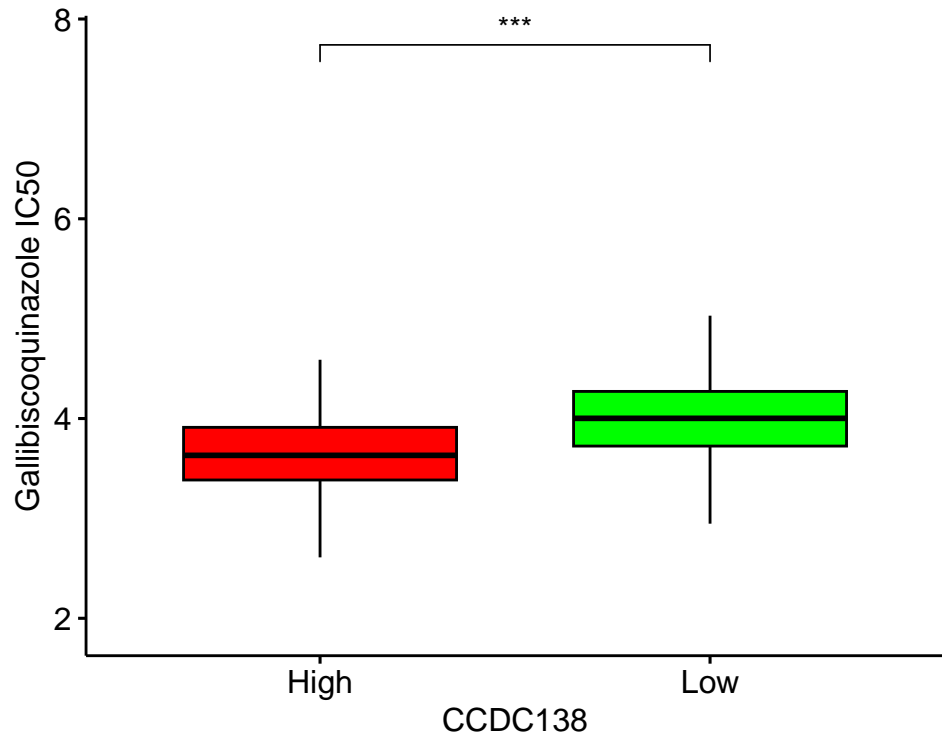

Supplement: Supplementary file 1 [file DataSheet1.zip › supplementary file/supplementary file 2/CCDC138_drugSenstivity.Gallibiscoquinazole.pdf]

CCDC138 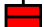 High 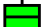 Low

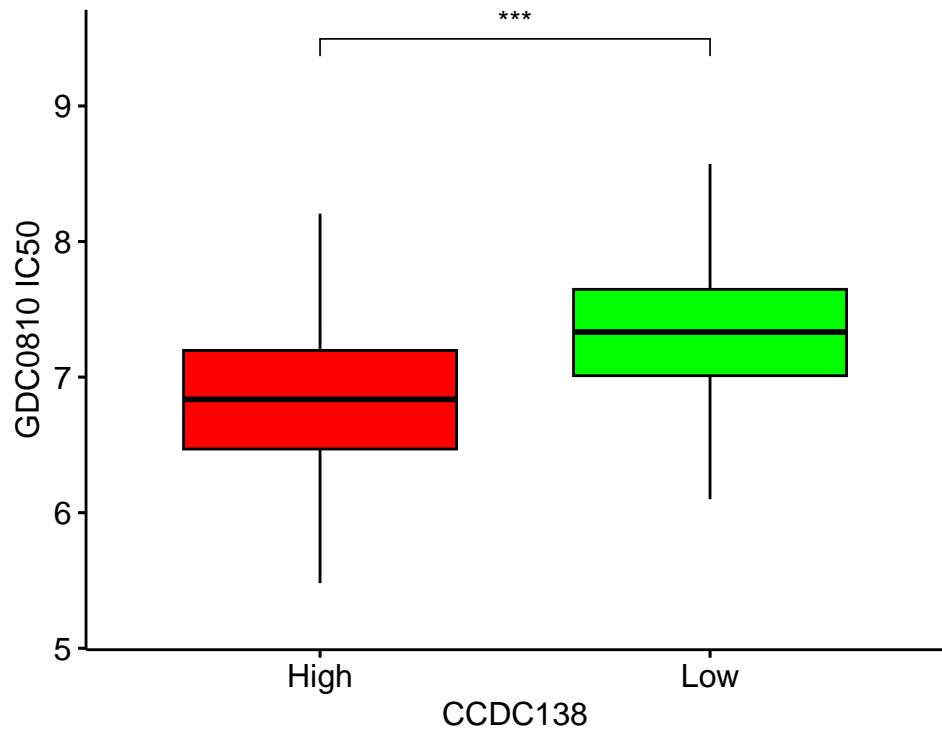

Supplement: Supplementary file 1 [file DataSheet1.zip › supplementary file/supplementary file 2/CCDC138_drugSenstivity.GDC0810.pdf]

CCDC138 High Low

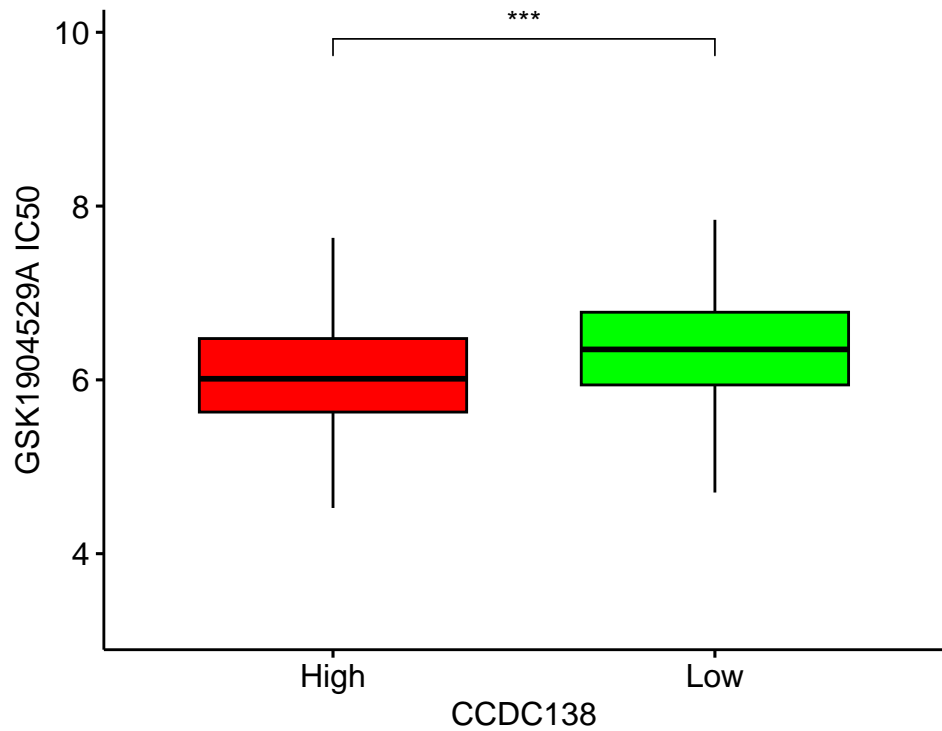

Supplement: Supplementary file 1 [file DataSheet1.zip › supplementary file/supplementary file 2/CCDC138_drugSenstivity.GSK1904529A.pdf]

CCDC138 High Low

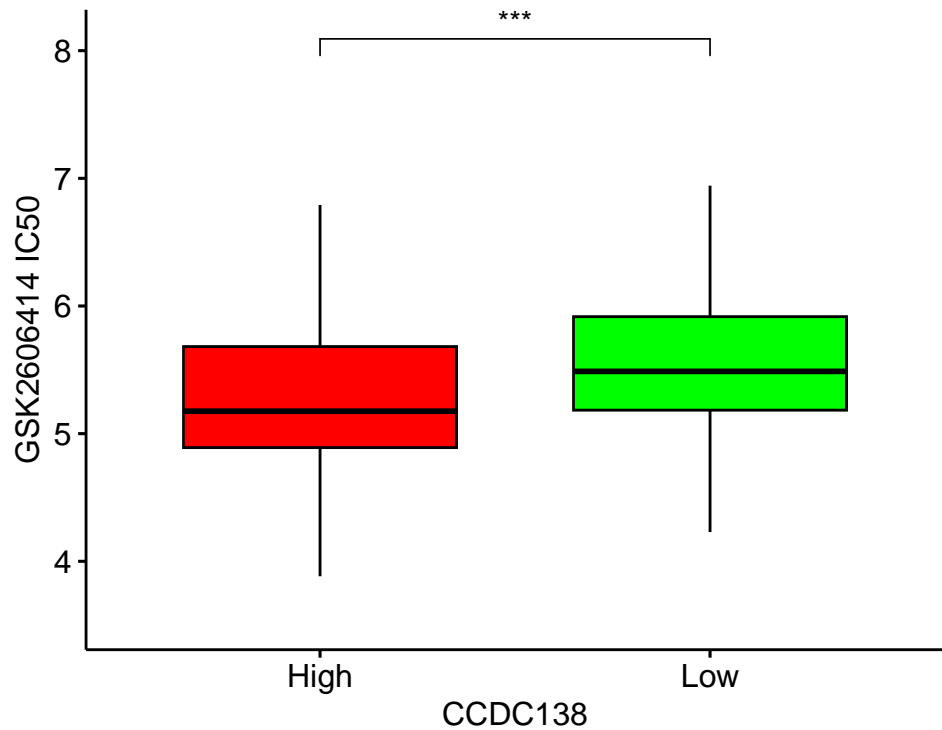

Supplement: Supplementary file 1 [file DataSheet1.zip › supplementary file/supplementary file 2/CCDC138_drugSenstivity.GSK2606414.pdf]

CCDC138 High Low

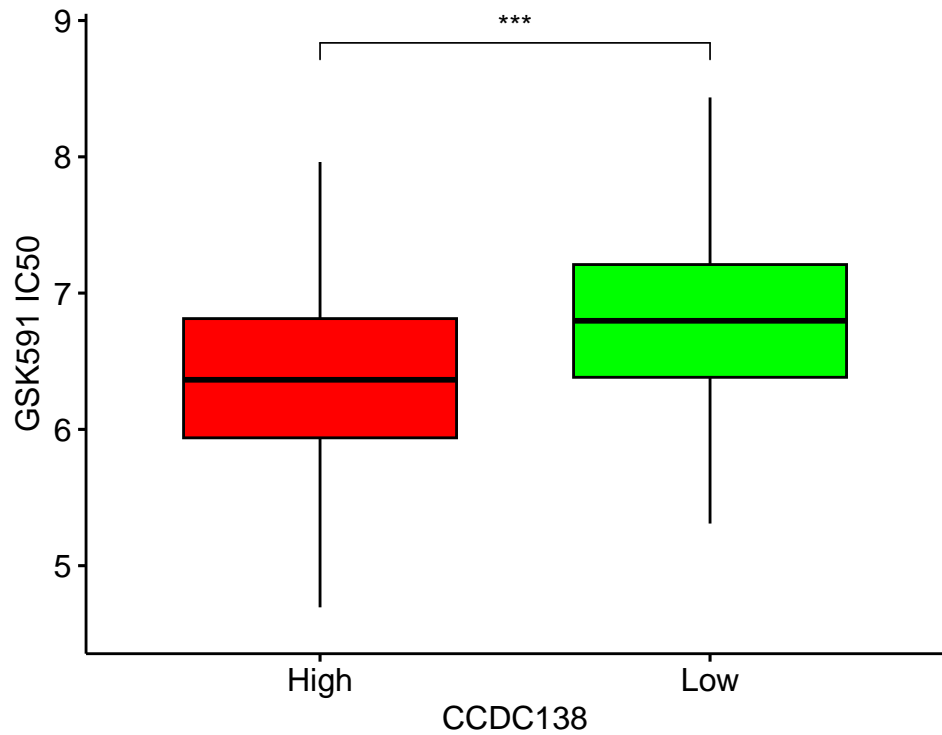

Supplement: Supplementary file 1 [file DataSheet1.zip › supplementary file/supplementary file 2/CCDC138_drugSenstivity.GSK591.pdf]

CCDC138 High Low

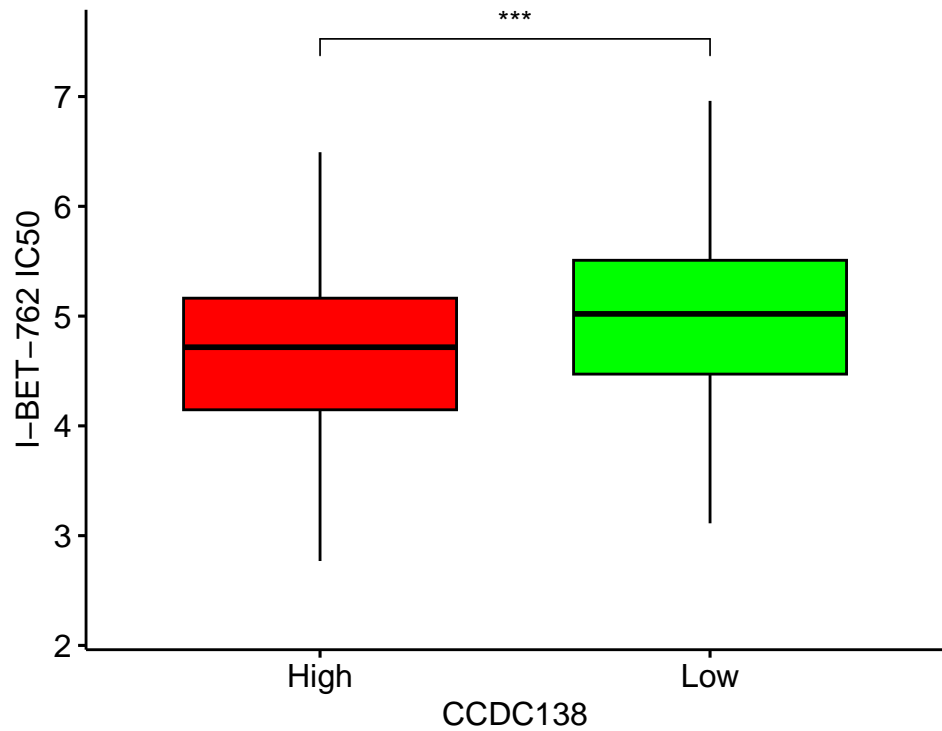

Supplement: Supplementary file 1 [file DataSheet1.zip › supplementary file/supplementary file 2/CCDC138_drugSenstivity.I-BET-762.pdf]

CCDC138 High Low

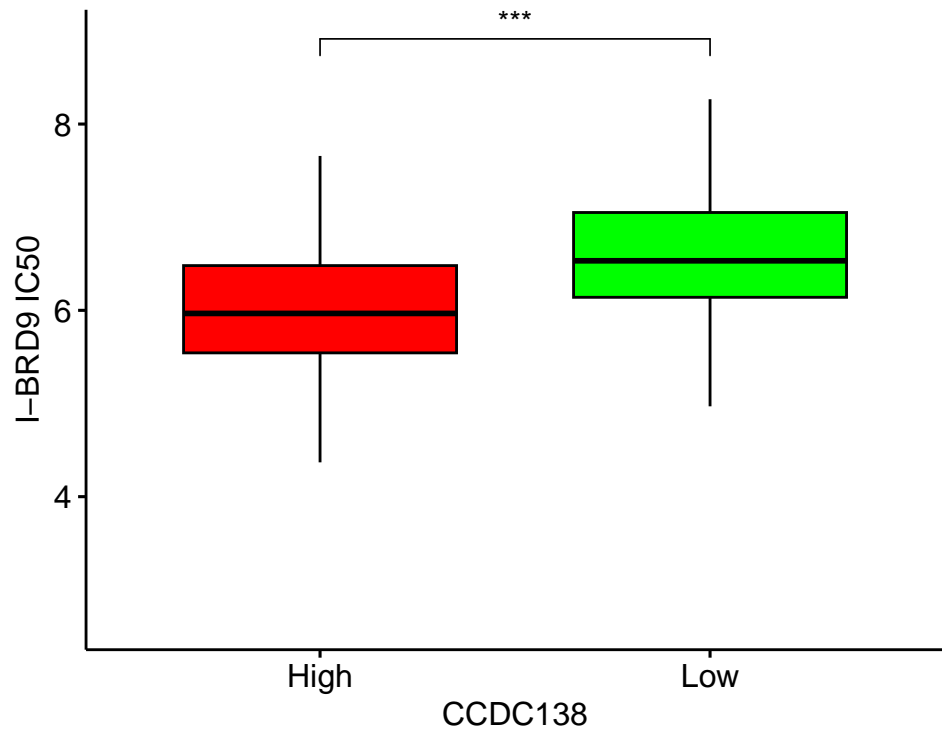

Supplement: Supplementary file 1 [file DataSheet1.zip › supplementary file/supplementary file 2/CCDC138_drugSenstivity.I-BRD9.pdf]

CCDC138 High Low

\*\*\*

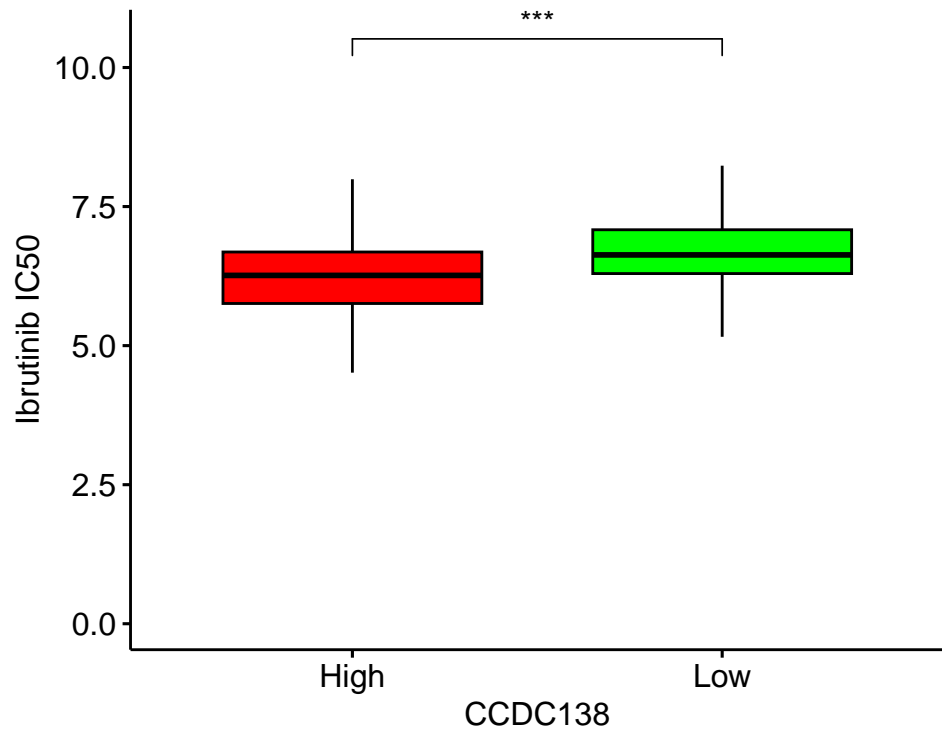

Supplement: Supplementary file 1 [file DataSheet1.zip › supplementary file/supplementary file 2/CCDC138_drugSenstivity.Ibrutinib.pdf]

CCDC138 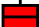 High 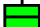 Low

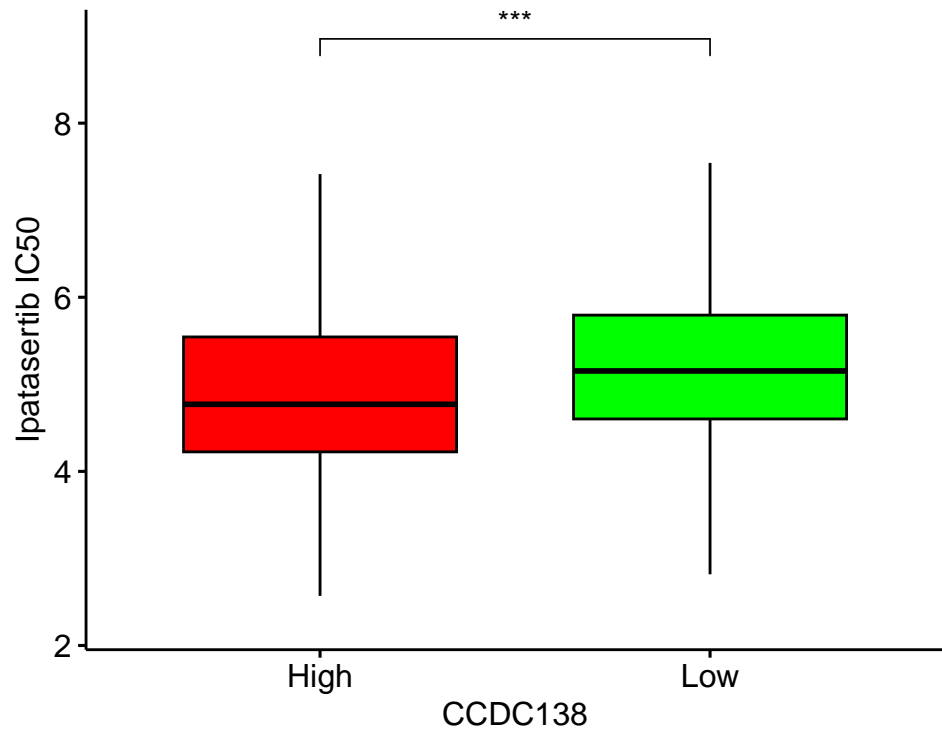

Supplement: Supplementary file 1 [file DataSheet1.zip › supplementary file/supplementary file 2/CCDC138_drugSenstivity.Ipatasertib.pdf]

KRAS (G12C) Inhibitor-12 IC50

CCDC138 High Low

\*\*\*

High

Low

CCDC138

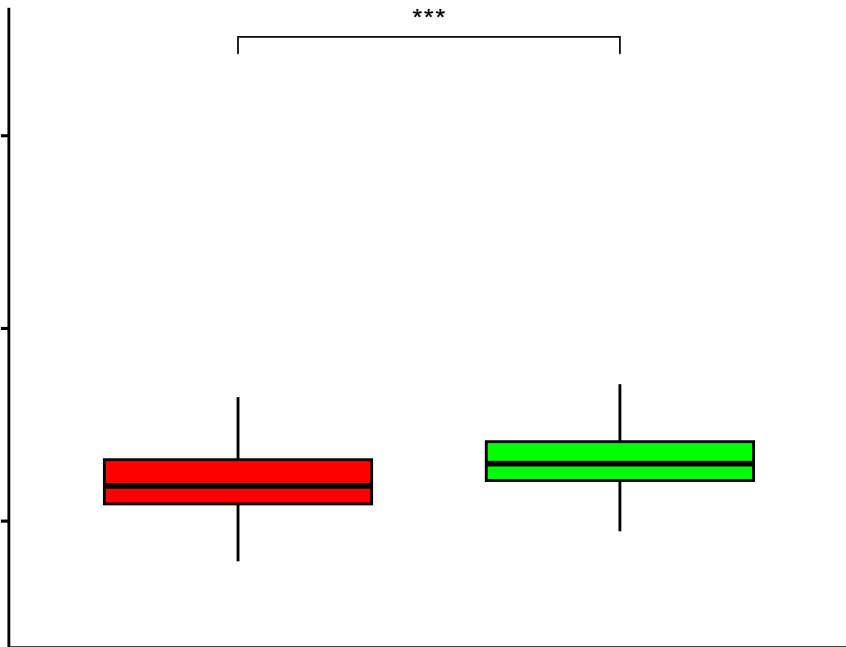

Supplement: Supplementary file 1 [file DataSheet1.zip › supplementary file/supplementary file 2/CCDC138_drugSenstivity.KRAS (G12C) Inhibitor-12.pdf]

CCDC138 High Low

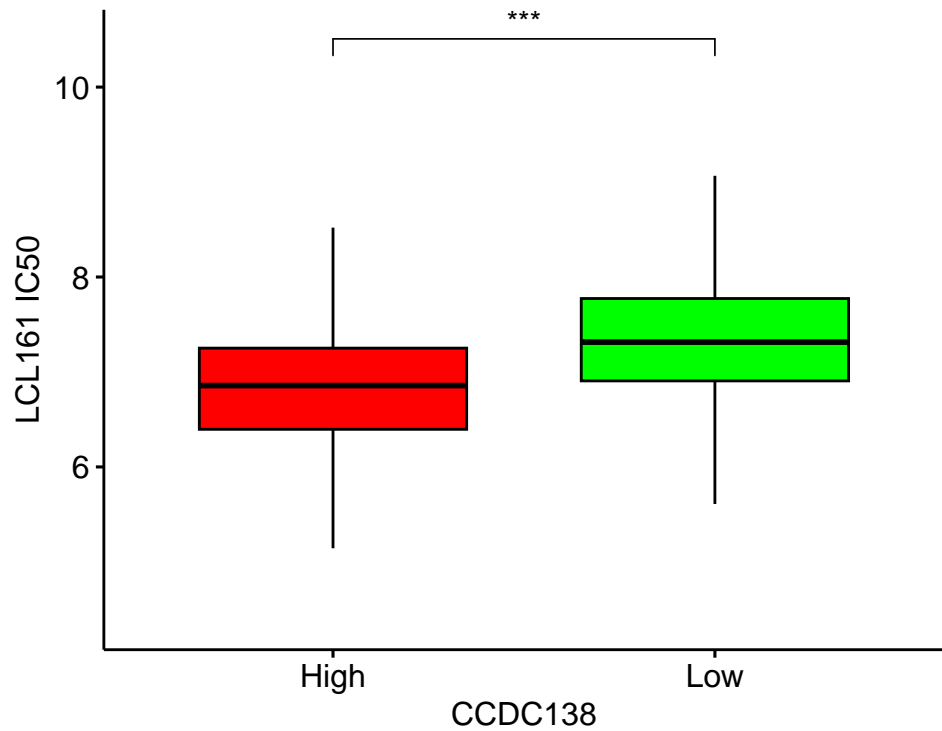

Supplement: Supplementary file 1 [file DataSheet1.zip › supplementary file/supplementary file 2/CCDC138_drugSenstivity.LCL161.pdf]

CCDC138 High Low

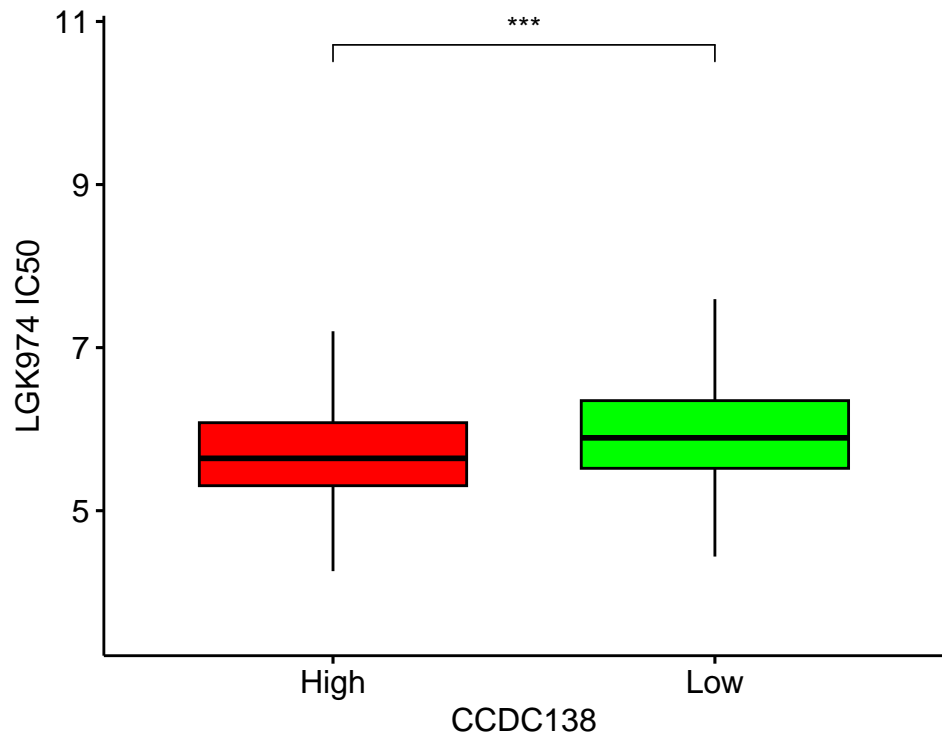

Supplement: Supplementary file 1 [file DataSheet1.zip › supplementary file/supplementary file 2/CCDC138_drugSenstivity.LGK974.pdf]

CCDC138 High Low

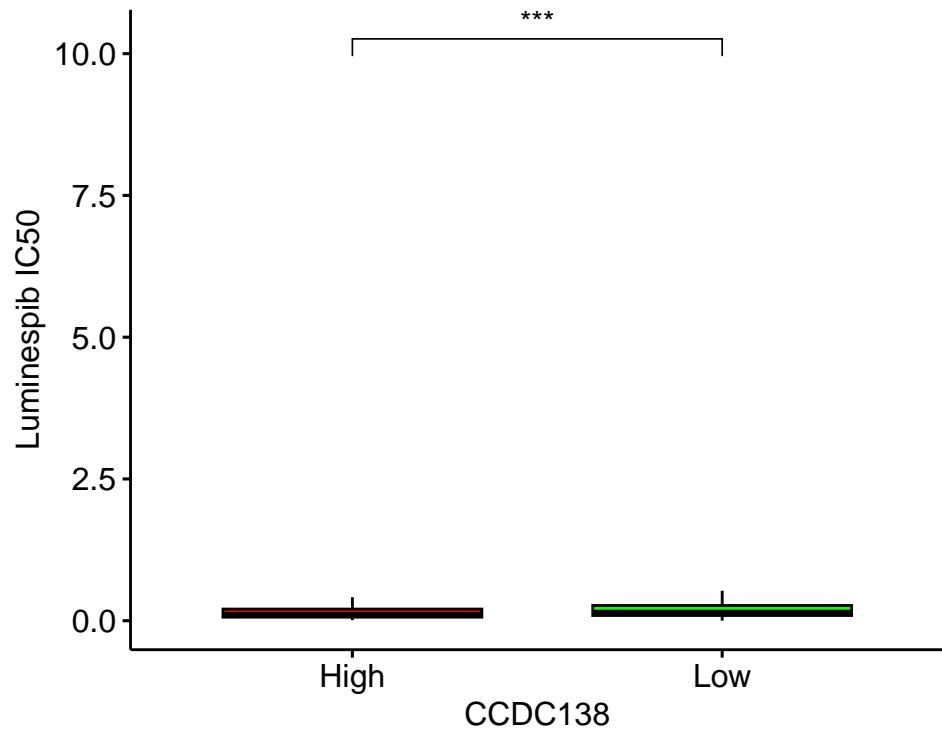

Supplement: Supplementary file 1 [file DataSheet1.zip › supplementary file/supplementary file 2/CCDC138_drugSenstivity.Luminespib.pdf]

CCDC138 High Low

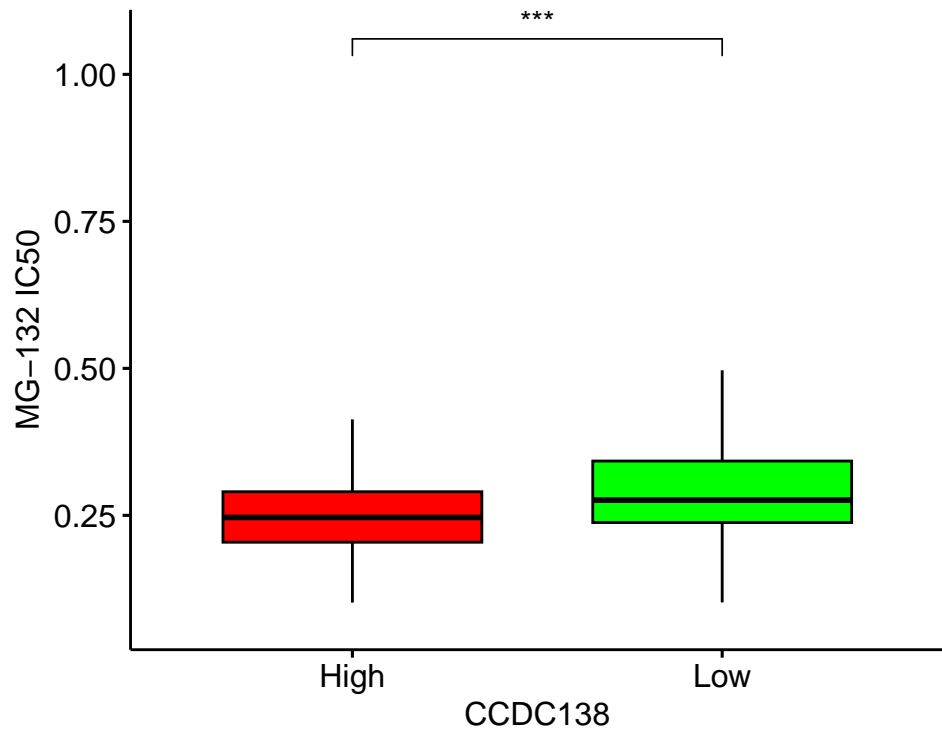

Supplement: Supplementary file 1 [file DataSheet1.zip › supplementary file/supplementary file 2/CCDC138_drugSenstivity.MG-132.pdf]

CCDC138 High Low

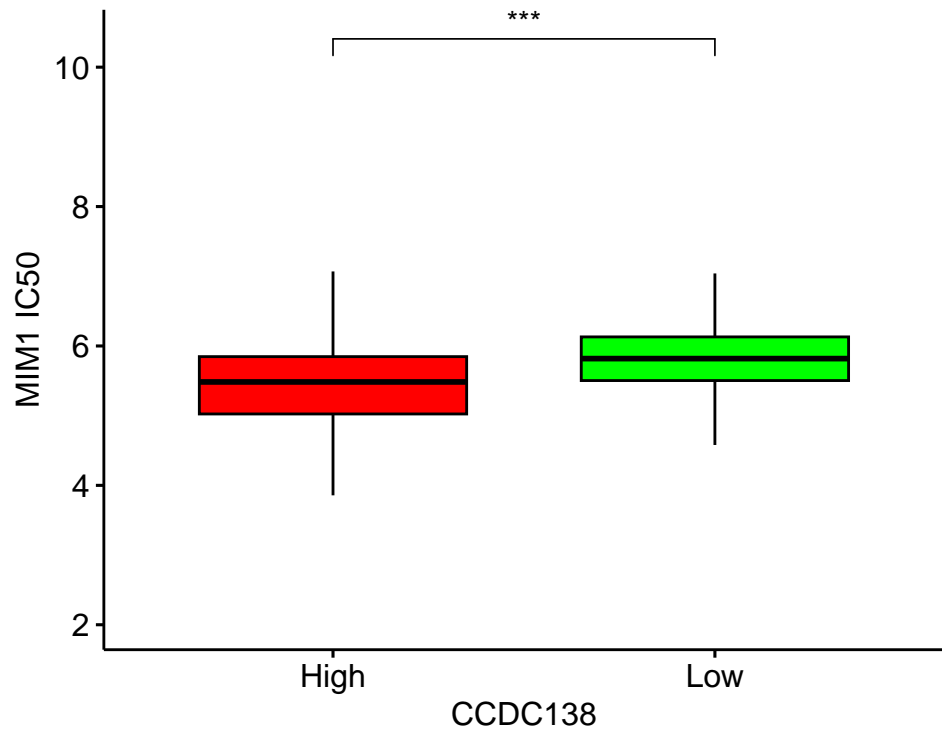

Supplement: Supplementary file 1 [file DataSheet1.zip › supplementary file/supplementary file 2/CCDC138_drugSenstivity.MIM1.pdf]

CCDC138 High Low

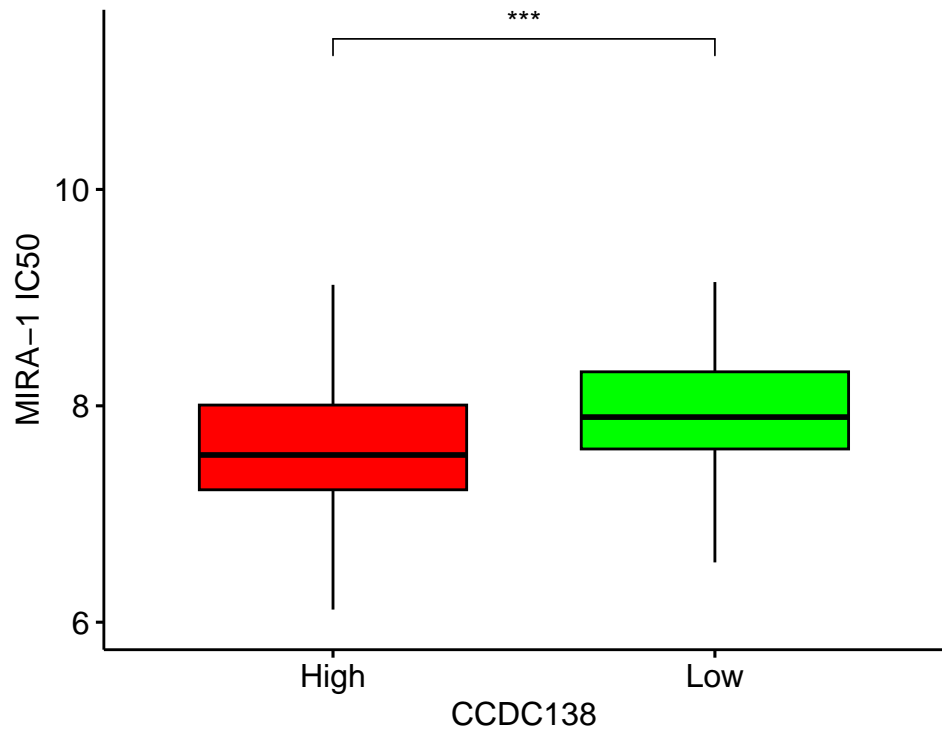

Supplement: Supplementary file 1 [file DataSheet1.zip › supplementary file/supplementary file 2/CCDC138_drugSenstivity.MIRA-1.pdf]

CCDC138 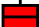 High 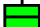 Low

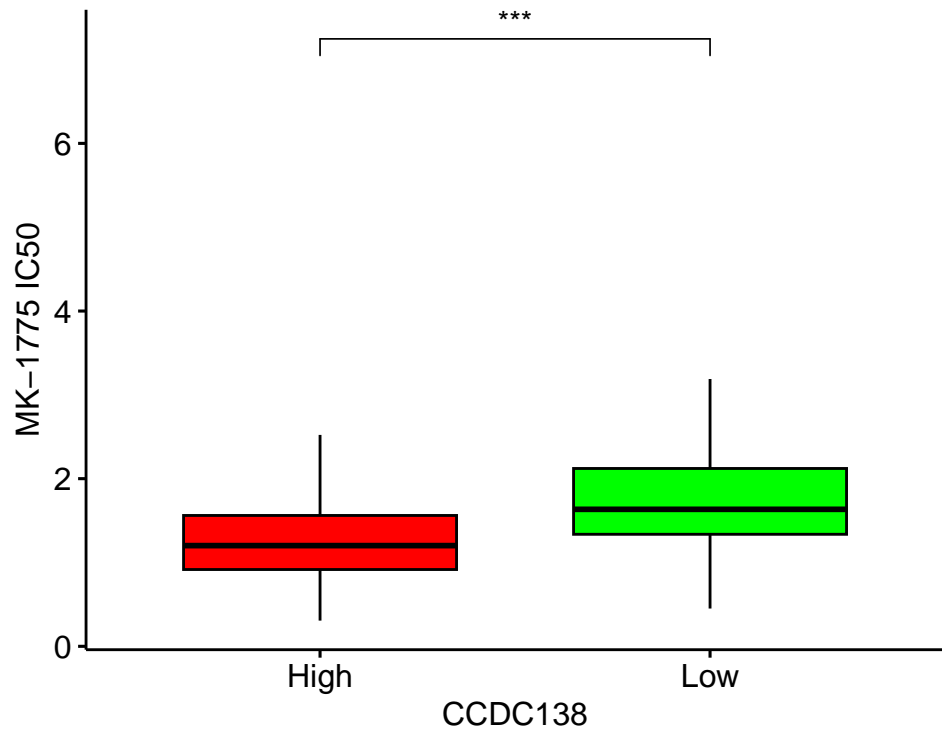

Supplement: Supplementary file 1 [file DataSheet1.zip › supplementary file/supplementary file 2/CCDC138_drugSenstivity.MK-1775.pdf]

CCDC138 High Low

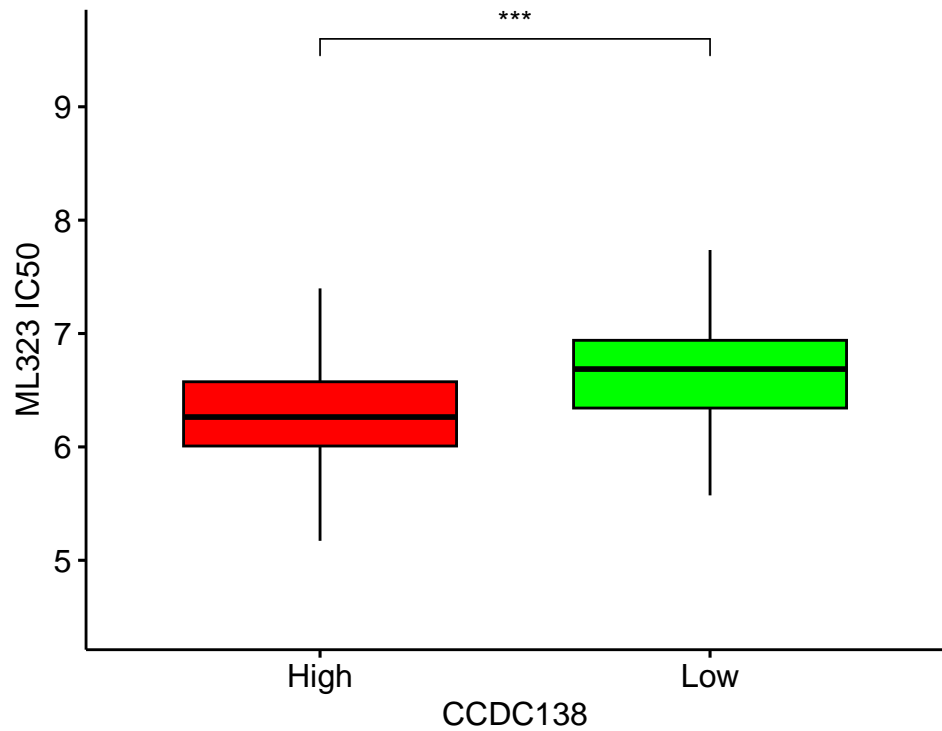

Supplement: Supplementary file 1 [file DataSheet1.zip › supplementary file/supplementary file 2/CCDC138_drugSenstivity.ML323.pdf]

CCDC138 High Low

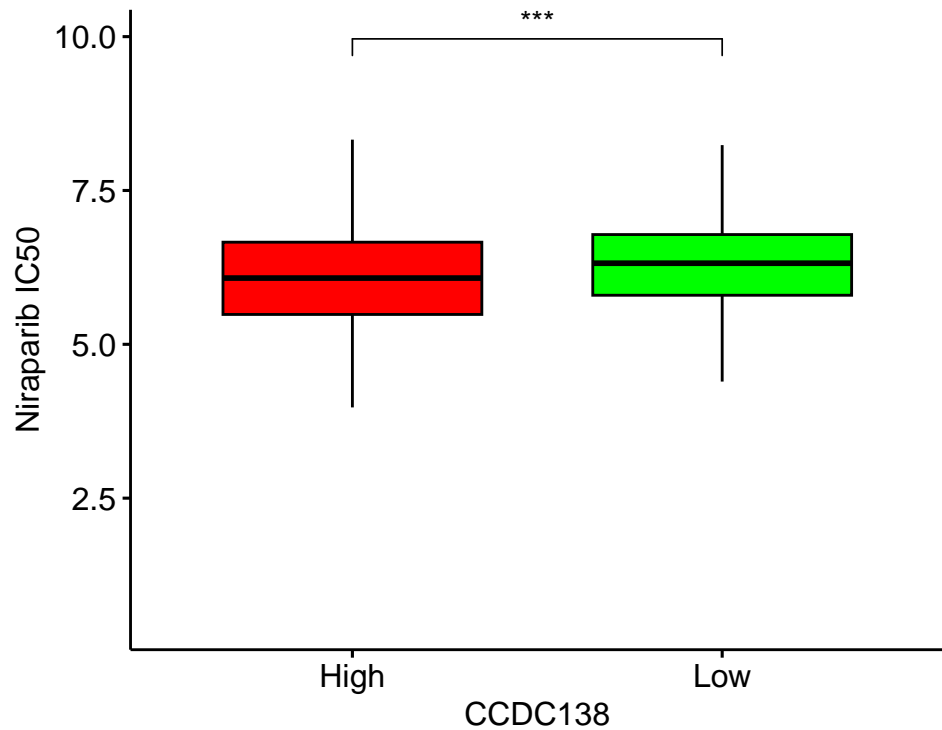

Supplement: Supplementary file 1 [file DataSheet1.zip › supplementary file/supplementary file 2/CCDC138_drugSenstivity.Niraparib.pdf]

CCDC138 High Low

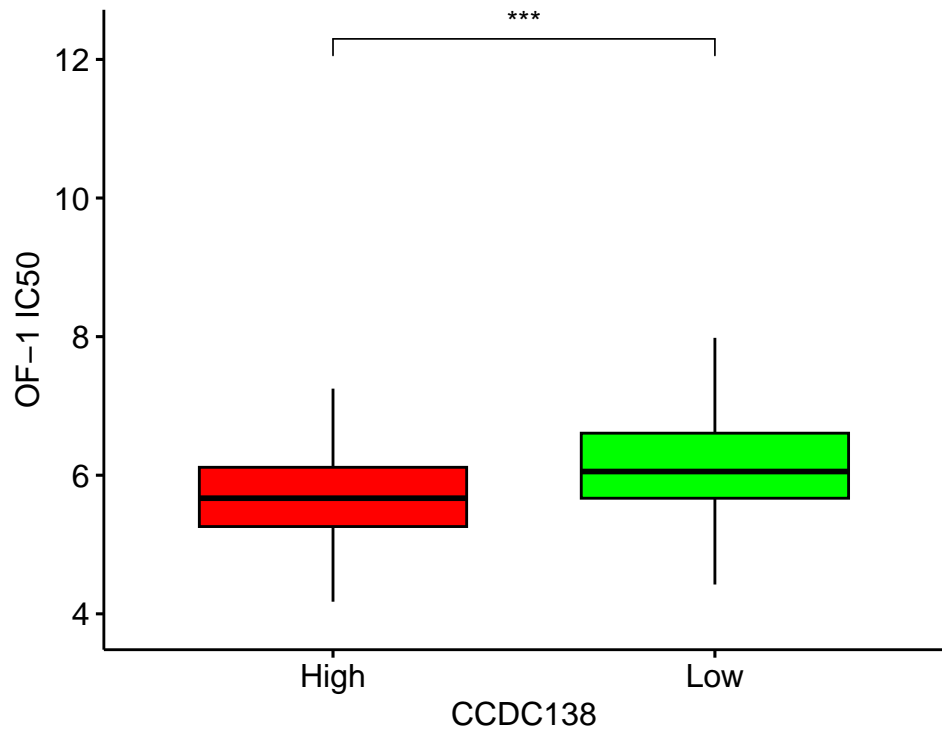

Supplement: Supplementary file 1 [file DataSheet1.zip › supplementary file/supplementary file 2/CCDC138_drugSenstivity.OF-1.pdf]

CCDC138 High Low

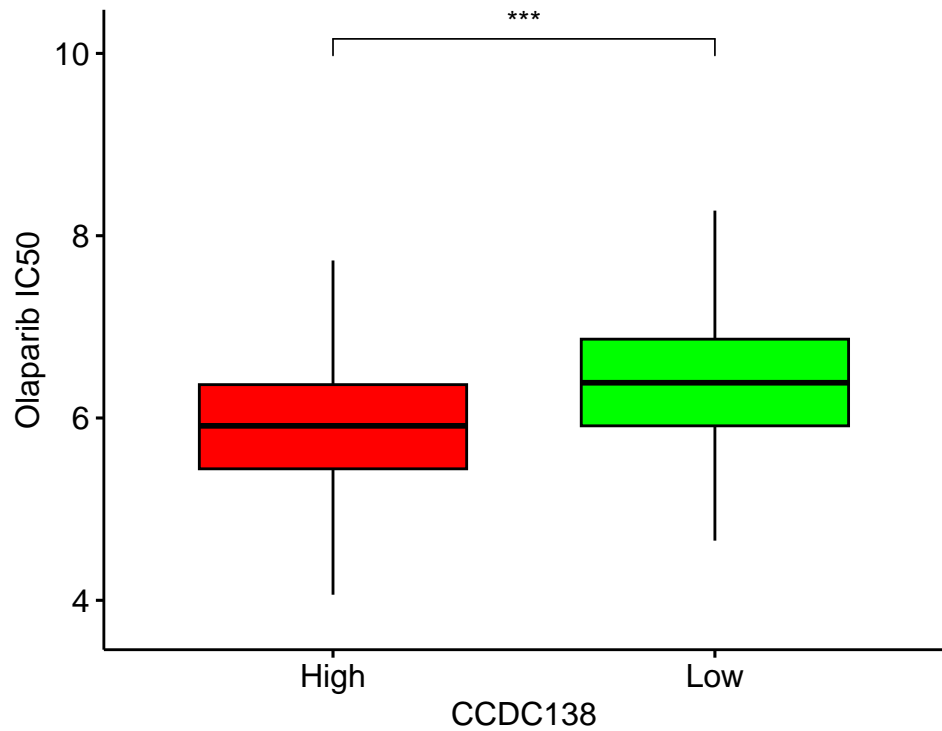

Supplement: Supplementary file 1 [file DataSheet1.zip › supplementary file/supplementary file 2/CCDC138_drugSenstivity.Olaparib.pdf]

CCDC138 High Low

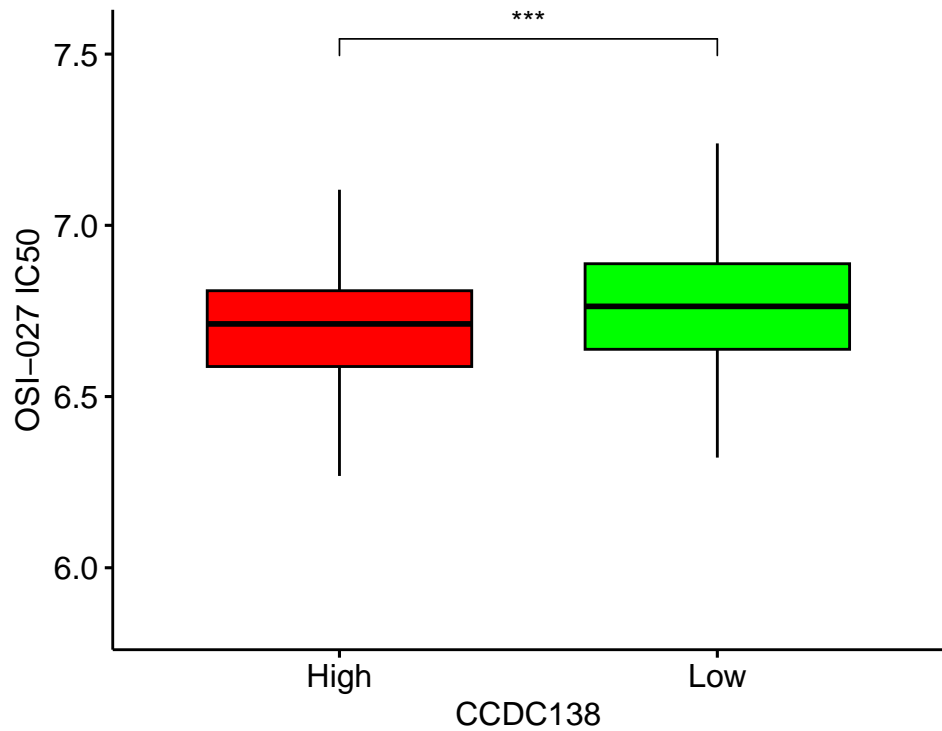

Supplement: Supplementary file 1 [file DataSheet1.zip › supplementary file/supplementary file 2/CCDC138_drugSenstivity.OSI-027.pdf]

CCDC138 High Low

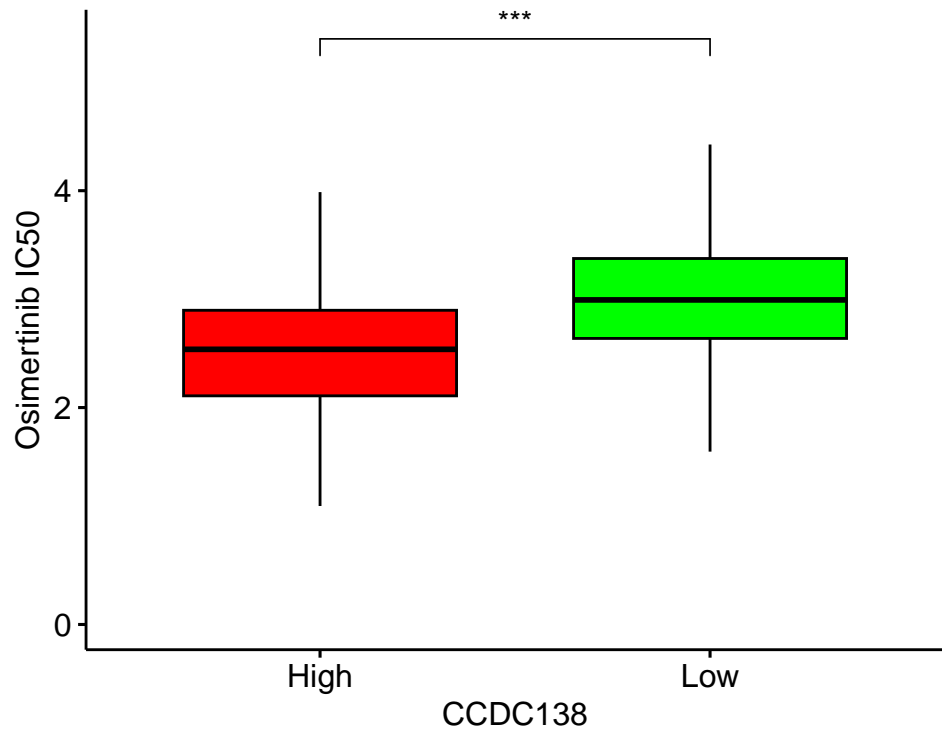

Supplement: Supplementary file 1 [file DataSheet1.zip › supplementary file/supplementary file 2/CCDC138_drugSenstivity.Osimertinib.pdf]

CCDC138 High Low

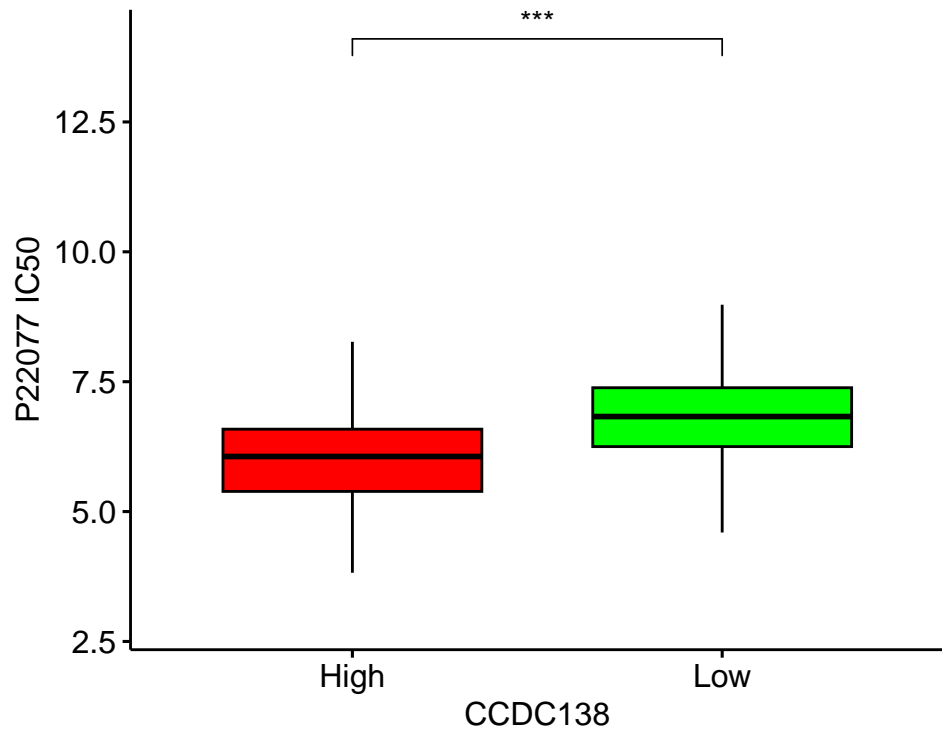

Supplement: Supplementary file 1 [file DataSheet1.zip › supplementary file/supplementary file 2/CCDC138_drugSenstivity.P22077.pdf]

CCDC138 High Low

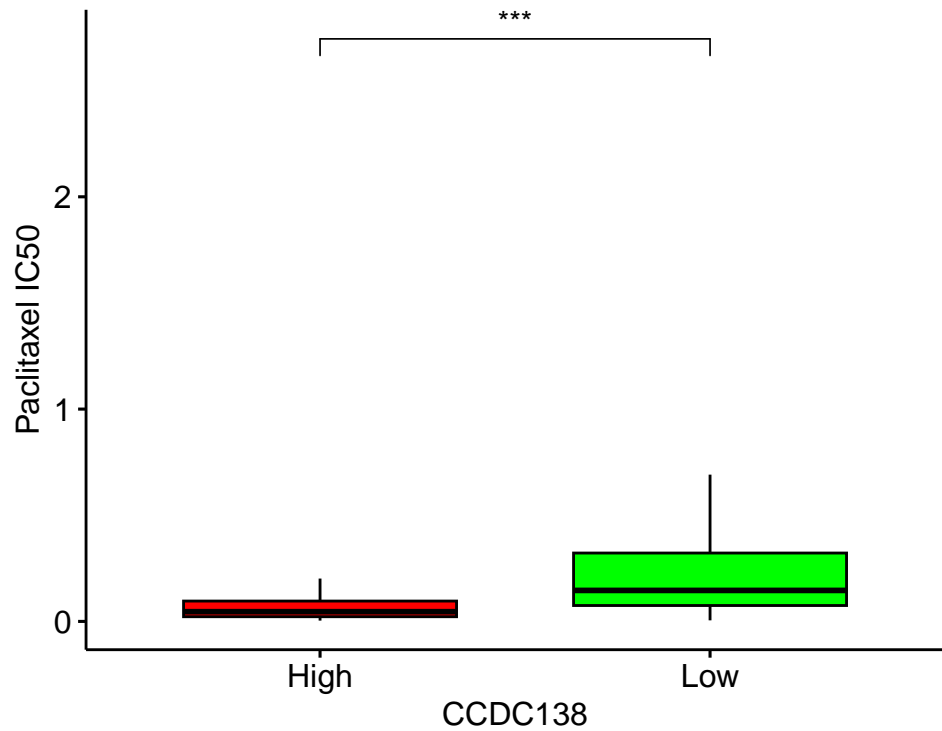

Supplement: Supplementary file 1 [file DataSheet1.zip › supplementary file/supplementary file 2/CCDC138_drugSenstivity.Paclitaxel.pdf]

CCDC138 High Low

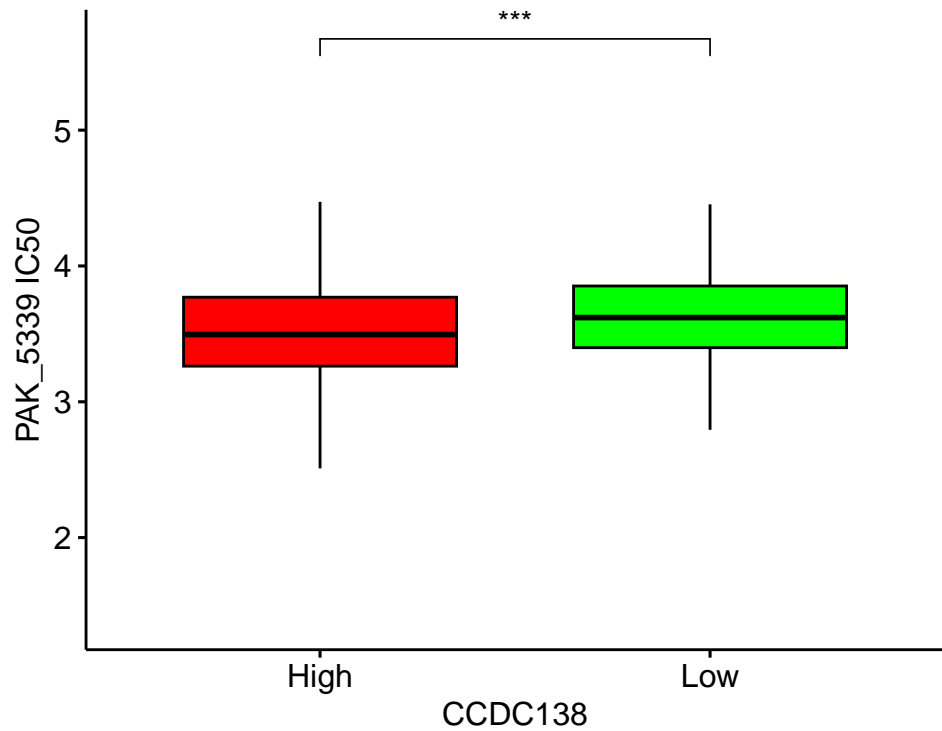

Supplement: Supplementary file 1 [file DataSheet1.zip › supplementary file/supplementary file 2/CCDC138_drugSenstivity.PAK_5339.pdf]

CCDC138 High Low

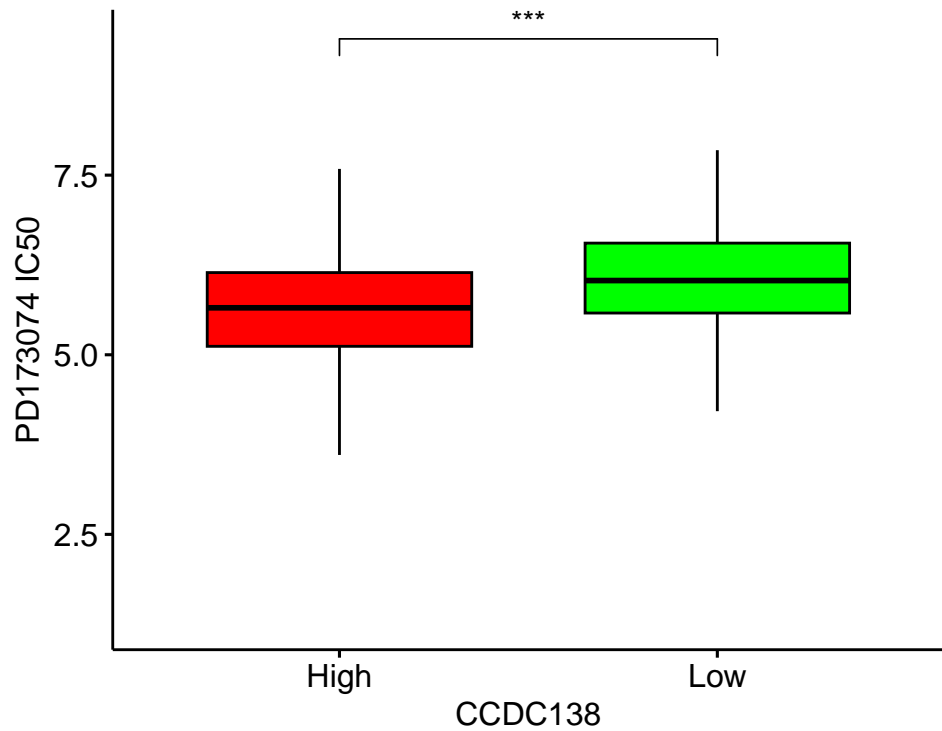

Supplement: Supplementary file 1 [file DataSheet1.zip › supplementary file/supplementary file 2/CCDC138_drugSenstivity.PD173074.pdf]
